# The relationship between single nucleotide polymorphisms and skin cancer susceptibility: A systematic review and network meta-analysis

**DOI:** 10.3389/fonc.2023.1094309

**Published:** 2023-02-15

**Authors:** Lu Zhang, Éva Pozsgai, Yongan Song, John Macharia, Huda Alfatafta, Jia Zheng, Zhaoyi Li, Hongbo Liu, István Kiss

**Affiliations:** ^1^ Department of Health Science, Doctoral School of Health Science, University of Pécs, Pécs, Hungary; ^2^ Department of Public Health Medicine, Doctoral School of Clinical Medicine, University of Pécs Medical School, Pécs, Hungary; ^3^ Department of Clinical Epidemiology, the Fourth Affiliated Hospital of China Medical University, Shenyang, China; ^4^ Faculty of Engineering and Information Technology, University of Pécs, Pécs, Hungary; ^5^ Department of Health Statistics, School of Public Health, China Medical University, Shenyang, China

**Keywords:** skin cancer, single-nucleotide polymorphisms, network meta-analysis, systematic review, melanoma

## Abstract

**Background:**

Single nucleotide polymorphisms (SNPs) interfere with the function of certain genes and thus may influence the probability of skin cancer. The correlation between SNPs and skin cancer (SC) lacks statistical power, however. Therefore, the purpose of this study was to identify the gene polymorphisms involved in skin cancer susceptibility using network meta-analysis and to determine the relationship between SNPs and SC risk.

**Methods:**

PubMed, Embase, and Web of Science were searched for articles including “SNP” and different types of SC as keywords between January 2005 and May 2022. The Newcastle-Ottawa Scale was used to assess bias judgments. The odds ratio (ORs) and their 95% confidence intervals (*CIs*) were determined to estimate heterogeneity within and between studies. Meta-analysis and network meta-analysis were carried out to identify the SNPs associated with SC. The *P*-score of each SNP was compared to obtain the rank of probability. Subgroup analyses were performed by cancer type.

**Results:**

A total of 275 SNPs from 59 studies were included in the study. Two subgroup SNP networks using the allele model and dominant model were analyzed. The alternative alleles of rs2228570 (FokI) and rs13181 (ERCC2) were the first-ranking SNPs in both subgroups one and two of the allele model, respectively. The homozygous dominant genotype and heterozygous genotype of rs475007 in subgroup one and the homozygous recessive genotype of rs238406 in subgroup two were most likely to be associated with skin cancer based on the dominant model.

**Conclusions:**

According to the allele model, SNPs FokI rs2228570 and ERCC2 rs13181 and, according to the dominant model, SNPs MMP1 rs475007 and ERCC2 rs238406 are closely linked to SC risk.

## 1 Introduction

The incidence of skin cancer has increased significantly since the 1970s, mainly due to lifestyle changes, including sun-seeking behavior and the thinning of the ozone layer (1). Skin cancers include cutaneous melanoma (CM) and nonmelanoma skin cancer (NMSC), with growing incidence rates for both cancer types (2). They are all caused by the abnormal growth of skin cells, especially those exposed to the sun. Non-melanoma skin cancer is the most common cancer among white-skinned people, and thus it is a significant cause of morbidity (3). Melanoma is less common; however, its prognosis is poorer, resulting in higher mortality rates (4). Approximately 1.2 million new NMSC (4) and nearly 300,000 new CM cases were diagnosed worldwide with 57,043 deaths from CM in 2020 (4). Timely diagnosis is crucial for reducing mortality from malignant melanoma and also has additional health and economic benefits (5). Since early detection of skin cancer is often limited, identifying suitable markers for its detection is of the utmost importance (6). Therefore, certain new genetic loci were investigated as possible markers for identifying SC risk (7).

Single nucleotide polymorphisms (SNPs) are genetic variations caused by point mutations. The allelic distribution of SNPs may interfere with the function of genes and then influence the probability of certain diseases (8, 9), which has led to SNPs being investigated as possible biological markers. Various SNPs have been shown to be associated with pigmentation, nevi, hair, skin color, and skin cancer. The SNPs of the BRAF and NRAS genes, for example, have been found to be commonly mutated oncogenes in CM (10). Furthermore, similarly to the interactions between genetics and the environment, the number and frequency of SNPs also affect the characteristics of their related genes as well as the development of their related disease phenotypes (11, 12).

Network meta-analysis (NMA), and in particular, Bayesian network meta-analysis, analyzes the direct and indirect evidence from multiple comparisons of tests within and between studies (13), making it possible to investigate the interactions between multiple comparisons of SNP tests.

Therefore, the aim of our study was to identify and compare the single nucleotide polymorphisms predominantly involved in skin cancer susceptibility by conducting a network meta-analysis.

## 2 Method

### 2.1 Search strategy and selection criteria

**Figure 1:**
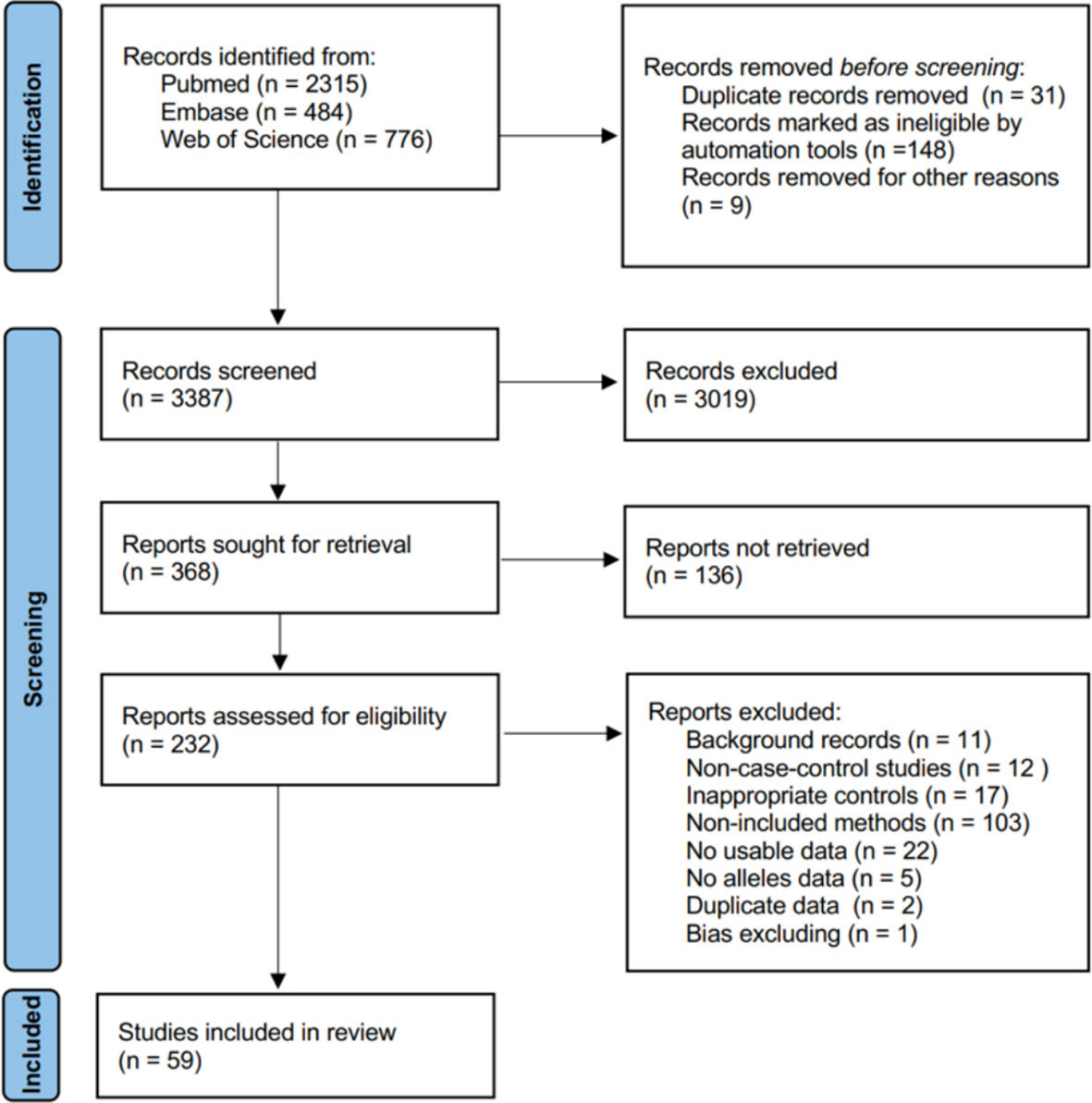
PRISMA flow diagram for network meta-analysis.

We searched the PubMed, Embase, and Web of Science electronic databases from their starting dates to May 2022 to identify relevant studies. The search strategy is shown in detail in **Supplementary Presentation 1**. We required the articles to include the following keywords: case–control, single nucleotide polymorphism (SNP), and study skin cancer (SC), cutaneous melanoma (CM), non-melanoma (NM), squamous cell carcinoma (SCC), or basal cell carcinoma (BCC). Inclusion and exclusion criteria are presented in **Supplementary Presentation 2**. The study was designed and performed in accordance with the PRISMA guidelines (**Figure 1**).

### 2.2 Data abstraction and bias assessment

Two researchers (LZ and YS) independently screened the titles and abstracts of the search results and extracted the following information from the included articles: authors’ names, year of publication, population of country and ethnicity, genotyping method, case and control numbers, control’s source, case–control match, cancer type, gene, SNP, and allele frequency.

**Figure 2:**
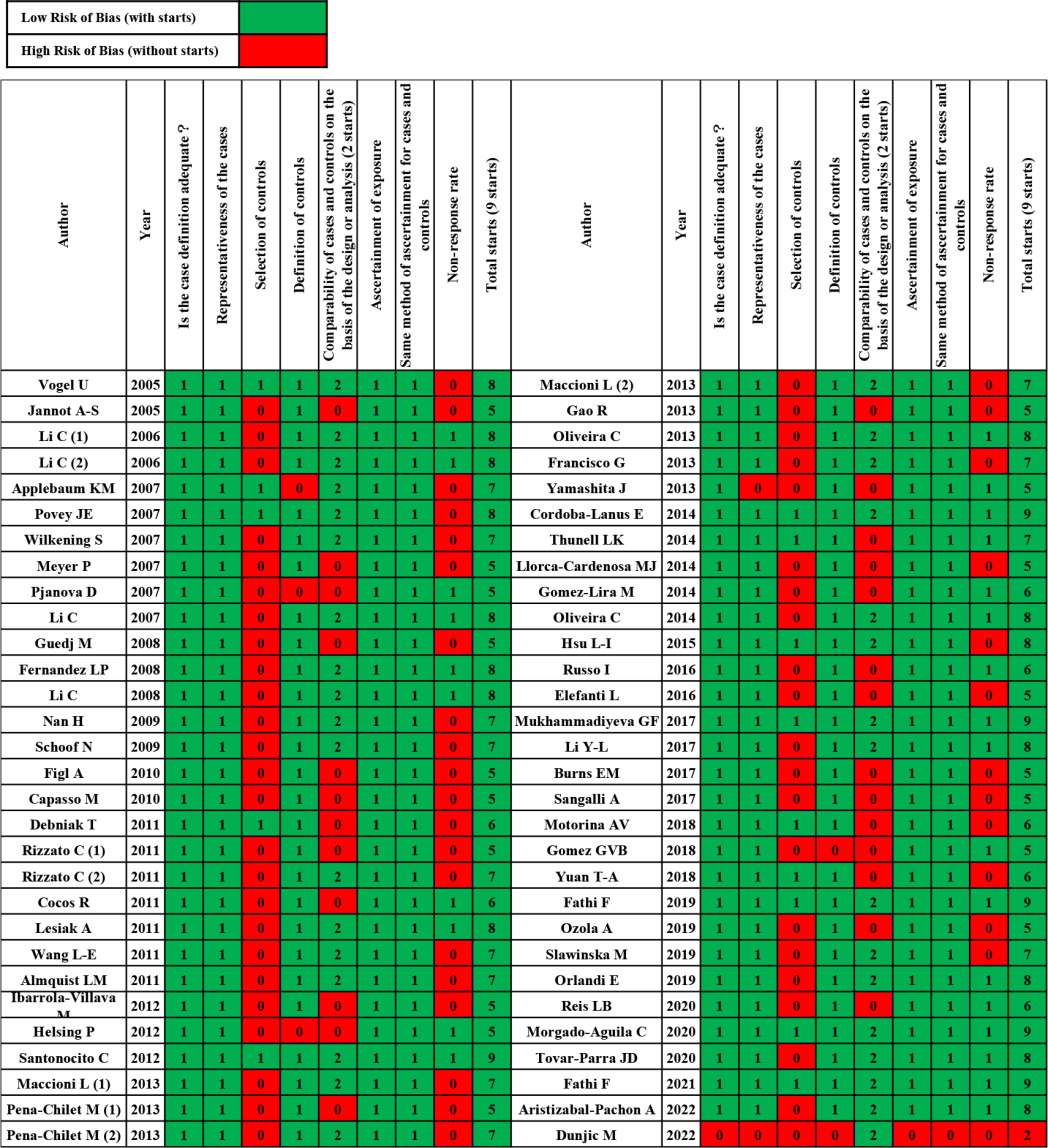
Case–control risks of the bias assessment graph.

Subsequently, we applied the Newcastle-Ottawa Scale (NOS) score for case-control studies to evaluate the quality and risk of bias of the included studies (**Figure 2**) (14). According to the NOS, article quality is assessed through eight questions from the Selection dimension (case definition, case selection, control definition, and control selection), the Comparability dimension (comparability of cases and controls), and the Exposure dimension (exposure ascertainment, case and control ascertainment, and non-response rate). Excepting “Comparability” with two stars, other items can each be given one star. Hence, a study can be awarded a maximum of nine stars and will be excluded if it receives fewer than five stars. Discrepancies were resolved by consensus between the reviewing authors.

### 2.3 Data synthesis and statistical analysis

Alleles are represented differently in different genomes. Therefore, for clarity, all reference alleles were represented by “A” in this study, and the corresponding alternative alleles were represented by “B.” Hence, for genotype, “AA” meant the homozygous dominant genotype, “AB” meant the heterozygote genotype, and “BB” meant the homozygous recessive genotype. The allele model (A vs. B) was employed to explore dominance. Furthermore, the dominant model (AA + AB vs. BB) and the recessive model (AA vs. AB + BB) were used for investigating the association between different genotypes and phenotypes (15).

A bivariate random effect model was performed for the meta-analysis of the comparative studies. Odds ratios (*OR*s) and their 95% confidence intervals (*CIs*) were used for estimating heterogeneity within and between studies. Then, pooled sensitivity (*Se*), specificity (*Sp*), positive likelihood ratio (*LR*), negative likelihood ratio (*LR*), diagnostic OR (DOR), and area under the summary receiver operating characteristic curve (AUROC) were calculated for each genotyping. The pooled AUROC was used as an indicator for testing to examine the diagnostic accuracy of each genotyping. The statistical heterogeneity between each study was assessed using the inconsistency index I-square. Additionally, meta-regression analysis was performed based on cancer type to assess heterogeneity. Cancer types included: CM, NMSC, and SC (including both CM and NMSC).

Next, Bayesian network meta-analysis (NMA) was used to clarify the relationships between the SNPs and skin cancer according to the allele model (A vs. B) and the dominant model (AA + AB vs. BB). The fixed-effects model that had four chains, 1,000 burn-ins, 200,000 iterations, and a thinning interval of 10 was selected for the MCMC simulation (16). The Gelman–Rubin plot and potential scale reduction factor (PSRF) were used for assessing convergence. Net splitting was carried out to check the consistency of the networks, and the effect estimate table was employed for estimating all SNP comparisons. Then, the overall ranks of SNPs were estimated by P-scores that were equivalent to the surface under the cumulative ranking curve (SUCRA) (17). SNPs with the highest P-scores were the most related to skin cancer.

*P <*0.05 was statistically significant. RStudio software and StataSE 16.0 software were used for calculations and plotting. The software packages used in the study are listed in **Supplementary Presentation 2**.

## 3 Results

### 3.1 Literature search results

The literature search initially identified 3,575 studies from PubMed, Embase, and Web of Science. The search ended on 2 May 2022. As **Figure 1** shows, we recorded screening 3,387 studies based on titles and abstracts, and 368 studies were obtained for full-text screening. At last, 59 studies met the inclusion criteria and were included in our network meta-analysis after being excluded from 232 full-text manuscripts due to background, unusable data, and bias reasons. One article was excluded due to bias, as explained below.

### 3.2 Characteristics and bias of enrolled studies

**Table 1** summarizes the main characteristics of 60 studies that were published between 2005 and 2022. Studies investigating Caucasian or Mongoloid ethnicities were included. **Figure 2** shows the quality assessment of enrolled studies using the NOS risk bias tool. Any studies with NOS scores lower than five stars were excluded. Finally, 59 articles were included in the systematic review and meta-analysis.

**Table 1:** Main characteristics of the eligible studies.

Author	Year	Country	Ethnicity	Genotyping method	Case/Control	Control’s Source	Match
Jannot (18)	2005	France	Caucasians	SNaPshot	120/125	HB	N
Vogel (19)	2005	Denmark	Caucasian	RT-PCR	322/322	PB	Y
Li (20)	2006	USA	Caucasian	PCR	602/603	HB	Y
Li (21)	2006	USA	Caucasian	PCR	602/603	HB	Y
Wilkening (22)	2007	Hungary Romania and Slovakia	Caucasian	TaqMan	517/523	HB	Y
Meyer (23)	2007	Germany	Caucasian	Sequencing Kit	632/615	HB	N
Applebaum (24)	2007	USA	Caucasian	Taqman	1540/780	PB	Y
Povey (25)	2007	UK	Caucasian	PCR-RFLP	596/441	PB	Y
Pjanova (26)	2007	Latvia	Caucasian	Sequencing Kit	203/125	HB	N
Li (27)	2007	USA	Caucasian	PCR	602/603	HB	Y
Li (28)	2008	USA	Caucasian	PCR	805/841	HB	Y
Fernandez (29)	2008	Spain	Caucasian	PCR	131/245	HB	Y
Guedj (30)	2008	France	Caucasian	PCR	1019/1466	HB	N
Nan (31)	2009	USA	Caucasian	PCR-RFLP	805/873	HB	Y
Schoof (32)	2009	Germany	Caucasian	TaqMan PCR	165/162	HB	Y
Figl (33)	2010	Germany and Spain	Caucasian	TaqMan	1186/1280	HB	Y
Capasso (34)	2010	Italy	Caucasian	PCR	249/291	HB	N
Debniak (35)	2011	Poland	Caucasian	Taqman	300/300	PB	N
Rizzato (36)	2011	Hungary, Romania, and Slovakia	Caucasian	RT-PCR	507/515	HB	N
Rizzato (37)	2011	Hungary, Romania, and Slovakia	Caucasian	Taqman	529/532	HB	Y
Lesiak (38)	2011	Poland	Caucasian	PCR-RFLP	142/142	HB	Y
Wang (39)	2011	USA	Caucasian	TaqMan	872/873	HB	Y
Almquist (40)	2011	USA	Caucasian	PCR-RFLP	1578/812	HB	Y
Ibarrola-Villava (41)	2012	Spain	Caucasian	TaqMan PCR	562/338	HB	N
Helsing (42)	2012	Norway	Caucasian	Sequencing Kit	388/420	HB	N
Santonocito (43)	2012	Italy	Caucasian	RT-PCR	167/186	PB	Y
Cocos (44)	2012	Romania	Caucasian	PCR-RFLP	174/80	HB	N
Gao (45)	2013	USA	Caucasian	PCR	312/216	HB	N
Oliveira (46)	2013	Brazil	Caucasian	PCR	146/146	HB	Y
Maccioni (47)	2013	Spain	Caucasian	PCR	837/1154	HB	Y
Pena-Chilet (48)	2013	Spain	Caucasian	RT-PCR	538/345	HB	N
Pena-Chilet (49)	2013	Spain	Caucasian	RT-PCR	530/314	HB	Y
Maccioni (50)	2013	Spain	Caucasian	PCR	837/1154	HB	Y
Francisco (51)	2013	Brazil	Caucasian	PCR-RFLP	202/210	HB	Y
Yamashita (52)	2013	Japan	Mongoloid	PCR	50/107	HB	N
Cordoba-Lanus (53)	2014	Spain	Caucasian	Sequencing Kit	509/491	PB	Y
Gomez-Lira (54)	2014	Italy	Caucasian	PCR-RFLP	240/342	HB	N
Oliveira (55)	2014	Brazil	Caucasian	PCR	100/108	HB	Y
Thunell (56)	2014	Sweden	Caucasian	PCR-RFLP	50/799	PB	Y
Llorca-Cardenosa (57)	2014	Spain	Caucasian	KASP PCR	648/381	HB	N
Hsu (58)	2015	China	Mongoloid	PCR-RFLP	70/210	PB	Y
Russo (59)	2016	Italy	Caucasian	RT-PCR	177/158	HB	N
Elefanti (60)	2016	Italy	Caucasian	TaqMan	182/89	HB	N
Mukhammadiyeva (61)	2017	Russia	Caucasian	PCR-RFLP	25/100	PB	Y
Li (62)	2017	China	Mongoloid	TaqMan	660/662	HB	Y
Burns (63)	2017	USA	Caucasian	PCR	97/100	HB	N
Sangalli (64)	2017	Italy	Caucasian	PCR	304/314	HB	N
Motorina (65)	2018	Russia	Caucasian	TaqMan PCR	95/334	PB	N
Gomez (66)	2018	Brazil	Caucasian	RT-PCR	250/250	HB	N
Yuan (67)	2018	USA	Caucasian	PCR	177/172	PB	N
Slawinska (68)	2019	Poland	Caucasian	PCR	254/254	HB	Y
Orlandi (69)	2019	Italy	Caucasian	PCR-RFLP	334/291	HB	Y
Ozola (70)	2019	Latvia	Caucasian	RT-PCR	253/200	HB	N
Fathi (71)	2019	Iranian	Caucasian	PCR-RFLP	210/320	PB	Y
Reis (72)	2020	Brazil	Caucasian	RT-PCR	120/135	HB	Y
Morgado-Aguila (73)	2020	Spain	Caucasian	Taqman	81/73	PB	Y
Tovar-Parra JD (74)	2020	Colombia	Caucasian	PCR	85/170	HB	Y
Fathi (75)	2021	Iranian	Caucasian	PCR-RFLP	210/220	PB	Y
Aristizabal-Pachon (76)	2022	Colombia	Caucasians	PCR-RFLP	120/120	HB	Y
Dunjic (77)^*^	2022	Serbian	Caucasians	RT-PCR	93/95	UN	Y

PCR, polvmerase chain reaction; PCR-RFLP, restriction fragment length polymorphism assay PCR; RT-PCR, real time PCR; KASP PCR, Competitive allele specific PCR; PB, population-based; HB, hospital-based; UN, unknown; Y, yes; N, no; ^*^Due to the low quality caused by bias, Dunjic’s article was excluded from the meta-analysis.

### 3.3 Pairwise meta-analysis

A direct meta-analysis was performed to determine the correlation between 275 SNPs and SC risk (**Supplementary Table 1**). A total of 72 SNPs from 47 studies were closely associated with SC in the studies using the allele model (A vs. B), while a significant association was found for 52 SNPs from 31 studies using the dominant model (AA + AB vs. BB). Furthermore, based on the recessive model (AA vs. AB + BB), 77 SNPs from 35 studies were related to SC. As depicted in **Supplementary Image 1**, the detected SNPs were analyzed further for diagnostic accuracy.

**Table 2** shows the evaluation of the diagnostic performance of the pooled SNPs for SC. According to SUCRA, the allele model can be employed for exploring dominance. Then, we chose the dominant model as the genotyping model for diagnosing SC.

**Table 2:** SNPs’ diagnostic performance evaluation in skin cancer.

	Alleles model (A vs. B)	Dominant model (AA + AB vs. BB)	Recessive model (AA vs. AB + BB)
Studies number	47	31	35
SNPs number	72	52	77
Pretest Prob	0.48	0.46	0.48
AUROC	0.50 [0.45, 0.54]	0.61 [0.57, 0.65]	0.53 [0.49, 0.57]
Sensitivity	0.79 [0.75, 0.83]	0.93 [0.91, 0.95]	0.64 [0.58, 0.69]
Specificity	0.22 [0.19, 0.26]	0.14 [0.11, 0.18]	0.42 [0.37, 0.47]
Positive LR	1.00 [1.00, 1.00]	1.1 [1.00, 1.10]	1.10 [1.00, 1.20]
Negative LR	0.94 [0.87, 1.02]	0.48 [0.33, 0.70]	0.85 [0.77,0.95]
Diagnostic OR	1 [1, 1]	2 [2, 3]	1 [1, 2]

“A” stands for the reference alleles; “B” stands for the corresponding alternative alleles; the numbers inside the “[,],” mean the range of 95% CI.

### 3.4 The allele model (A vs. B)

The associations between the 72 SNPs and SC susceptibility are shown in **Supplementary Table 2**. In the allele model, the reference alleles of rs16891982 (G vs. C, combined OR [cOR] = 2.74, 95% CI [2.20, 3.40]), rs885479 (G vs. A, cOR = 1.46, 95% CI [1.06, 2.01]), rs1544410 (G vs. A, cOR = 1.19, 95% CI [1.06, 1.34]), rs731236 (T vs. C, cOR = 1.11, 95% CI [1.00, 1.23]), and the alternative alleles of rs25487 (G vs. A, cOR = 0.92, 95% CI [0.85, 0.99]), rs4911414 (G vs. T, cOR = 0.85, 95% CI [0.75, 0.96]), rs1695 (W vs. M, cOR = 0.79, 95% CI [0.65, 0.95]), and rs2228570 (wild-type allele vs. mutant allele, cOR = 0.79, 95% CI [0.71, 0.88]) were related significantly to SC in at least two of the studies. The pooled *P*-value for all SNPs was less than 0.05.

### 3.5 The dominant model (AA + AB vs. BB)

**Supplementary Table 3** summarizes the 52 SNPs’ cOR for SC according to the dominant model. The results show that those who were homozygous dominant and heterozygous genotypes: rs16891982 (GG + GC vs. CC, cOR = 3.72, 95% CI [1.66, 8.35]), rs494379 (TT + TC vs. CC, cOR = 2.62, 95% CI [1.96, 3.49]), rs514921 (AA + AG vs. GG, cOR = 2.14, 95% CI [1.67, 2.75]), rs1144393 (AA + AG vs. GG, cOR = 1.48, 95% CI [1.19, 1.84]), rs11615 (AA + AG vs. GG, cOR = 1.41, 95% CI [1.02, 1.95]), and rs498186 (TT + TG vs. GG, cOR = 1.35, 95% CI [1.10, 1.65]) had a higher risk for developing SC, than those who were homozygous recessive genotypes. In contrast, individuals with the homozygous recessive genotypes of rs25487 (GG + GA vs. AA, cOR = 0.85, 95% CI [0.72, 1.00]) and rs1805007 (CC + CT vs. TT, cOR = 0.42, 95% CI [0.19, 0.91]) were significantly associated with increased susceptibility to SC.

### 3.6 Subgroup analysis

A covariate regression analysis was performed for each of the three genotypes. The results showed that there was no statistical difference (all *P*-values > 0.05) among CM, NMSC, and SC (including both CM and NMSC) (**Table 3**).

**Table 3:** Subgroup analyses according to the cancer type.

	Study number	Sensitivity [95% CI]	*p*	Specificity [95% CI]	*p*	LRTChi^2^	*p*
Allele model	121	0.85 [0.78, 0.91]	0.15	0.17 [0.11, 0.25]	0.28	3.31	0.19
Dominant model	76	0.95 [0.90, 0.97]	0.44	0.19 [0.11, 0.31]	0.28	3.16	0.21
Recessive model	132	0.77 [0.65, 0.86]	0.05	0.35 [0.25, 0.47]	0.29	5.88	0.05

LRTChi^2^, Likelihood ratio test in joint model.

### 3.7 Network evidence

#### 3.7.1 The allele model

**Figure 3:**
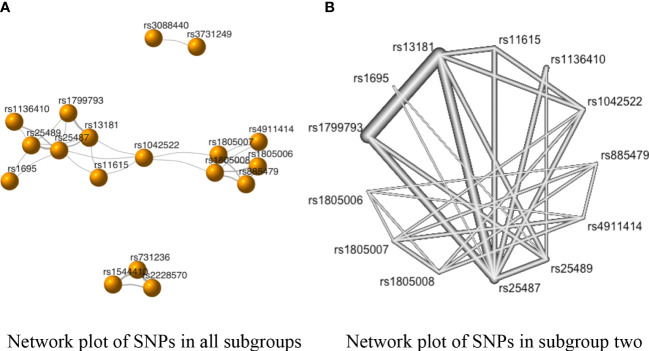
The network evidence plots of single nucleotide polymorphisms (SNPs) in the allele model (**A** vs. **B**). **(A)** Network plot of SNPs in all subgroups in the allele model; **(B)** Network plot of SNPs in subgroup two of the allele model.

The network plot depicts the rough comparison of each pair of SNPs (**Figure 3**). A node indicates an SNP, and its size represents the number of studies. The connections between the nodes represent a pair of comparisons, and their thickness represents the number of direct comparisons. As is evident from **Figure 3A**, there were three subgroups without any connections. Also, to avoid redundancy, the network of SNPs from one study was deleted from our study. Thus, the NMA of the allele model was divided into two groups: subgroup one (including rs1544410, rs2228570, and rs731236), and subgroup two (including rs1042522, rs1136410, rs11615, rs13181, rs1695, rs1799793, rs1805006, rs1805007, rs1805008, rs25487, rs25489, rs4911414, and rs885479) (**Figure 3B**).

The SNPs rs731236 vs. rs2228570 had the strongest negative correlation with SC risk in subgroup one (standardized mean differences (SMD) of OR = −0.08, 95% CI [−0.18, 0.02]) (**Supplementary Table 4**). However, the *P*-values of the correlations between the SNPs in subgroup one were above 0.05 (**Supplementary Table 4**). Similarly, as shown in **Figure 3** and **Supplementary Table 4**, the comparison with the highest direct pooled effect size in subgroup two was rs4911414 vs rs1805006 (SMD of OR = −2.94, 95% CI [−2.48, −3.40]), followed by comparison rs13181 vs. rs25489 (SMD of OR = −2.35, 95% CI [−2.54, −2.16]).

Additionally, in subgroup two, the direct and indirect evidence showed negative correlations in the comparisons of rs1042522 vs. rs25487, rs1136410 vs. rs25489, rs11615 vs. rs13181, rs11615 vs. rs25487, rs13181 vs. rs1799793, rs13181 vs. rs25487, rs1805007 vs. rs1805006, and rs1805007 vs. rs885479 (**Table 2** in **Supplementary Table 4**). However, since the indirect evidence proportion of each comparison (i.e., the mean path length of each estimated comparison) was less than 2 (78), each of the above-mentioned comparisons followed the direction of direct evidence (**Supplementary Image 2**).

To select the SNPs with the highest chance of a significant association with skin cancer, the *P*-scores were ranked, as shown in **Table 4**. The SNP rs2228570 (*P*-score = 0.85) ranked first in subgroup one in the allele model, and the SNP rs13181 had the highest *P*-score in subgroup two (*P*-score = 0.94).

**Table 4:** The rank of the *P*-score of the SNPs in each subgroup in the allele model.

Rank	Subgroup 1	*P*-score	Subgroup 2	*P*-score
1	rs2228570	0.85	rs13181	0.94
2	rs1544410	0.47	rs1799793	0.90
3	rs731236	0.18	rs25487	0.88
4			rs11615	0.77
5			rs1042522	0.64
6			rs1695	0.57
7			rs4911414	0.54
8			rs1136410	0.41
9			rs1805007	0.33
10			rs1805008	0.24
11			rs25489	0.19
12			rs885479	0.08
13			rs1805006	0.00

#### 3.7.2 The dominant model

**Figure 4:**
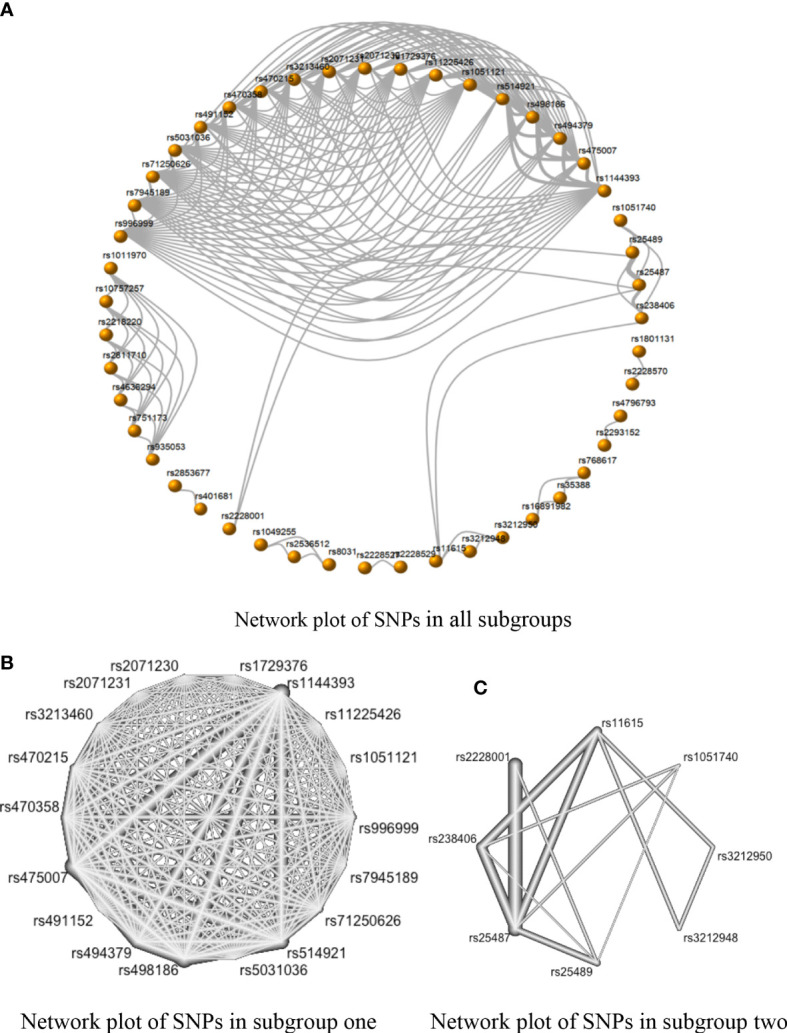
The network evidence plots of single nucleotide polymorphisms (SNPs) in the dominant model (AA + AB vs. BB). **(A)** Network map with 47 SNPs of all subgroups in the dominant model; **(B)** Network map of subgroup one with 18 SNPs in the dominant model; and **(C)** Network map of Subgroup two with eight SNPs in the dominant model.

In **Figure 4A**, only two subgroups met the requirements for the NMA. Subgroup one included rs1051121, rs11225426, rs1144393, rs1729376, rs2071230, rs2071231, rs3213460, rs470215, rs470358, rs475007, rs491152, rs494379, rs498186, rs5031036, rs514921, rs71250626, rs7945189, and rs996999 (**Figure 4B**), while subgroup two included rs1051740, rs11615, rs2228001, rs238406, rs25487, rs25489, rs3212948, and rs3212950 (**Figure 4C**).

There was no inconsistency between the direct and indirect evidence in Group 1. The strongest positive correlations in this subgroup were the comparison of rs475007 vs. rs1729376 and the comparison of rs475007 vs. rs2071231 (both SMDs of network OR = 4.23, 95% CI [2.19, 6.25]). These were followed by the rs475007 vs rs491152 comparison, which SNPs were negatively correlated with SC risk (SMD of network OR = −4.21, 95% CI [−6.24, −2.18]). The comparison of rs494379 and rs514921 showed the strongest indirect correlation (SMD of indirect OR = 11.52, 95% CI [−9.40, 32.44]) (**Supplementary Image 3–5**).

**Figure 5:**
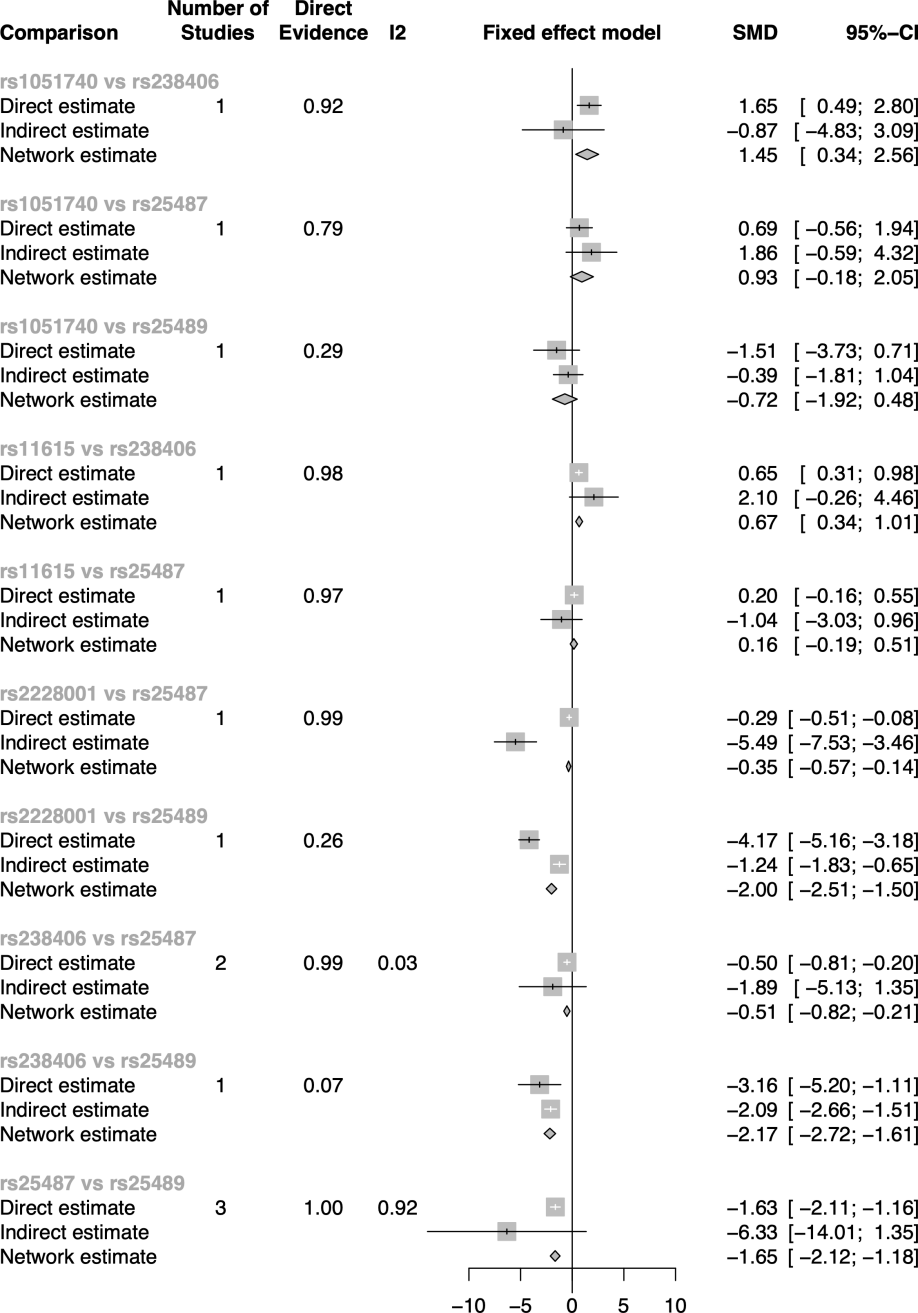
The direct and indirect evidence forest plot of subgroup two in the dominant model.

Inconsistencies between direct and indirect evidence were found in the comparison of rs2228001 vs. rs25487 and the comparison of rs2228001 vs. rs25489 in subgroup two (**Figure 4** in **Figure 5**). Both rs25487 and rs25489 were negatively correlated with rs2228001 after performing network analysis (**Figure 5**). **Figure 5** also demonstrates that the rs238406 vs. rs25489 comparison had the strongest association (SMD of network OR = −2.17, 95% CI [−2.72, −1.61]).

As shown in **Table 5**, rs475007 has the highest P-score (0.97) in subgroup one, and rs238406 has the highest P-score (0.97) in subgroup two. Therefore, the top five SNPs most likely associated with skin cancer are, in descending order, in subgroup one: rs475007, rs470358, rs498186, rs1144393, and rs470215, and in subgroup two: rs238406, rs2228001, rs25487, rs11615, and rs3212950.

**Table 5:** The rank of the *P*-score of the SNPs in each subgroup in the dominant model.

Rank	Subgroup 1	*P*-score	Subgroup 2	*P*-score
1	rs475007	0.97	rs238406	0.97
2	rs470358	0.92	rs2228001	0.87
3	rs498186	0.89	rs25487	0.62
4	rs1144393	0.84	rs11615	0.50
5	rs470215	0.79	rs3212950	0.41
6	rs514921	0.68	rs3212948	0.41
7	rs71250626	0.62	rs1051740	0.21
8	rs494379	0.59	rs25489	0.02
9	rs996999	0.58		
10	rs3213460	0.42		
11	rs2071230	0.27		
12	rs7945189	0.27		
13	rs11225426	0.26		
14	rs5031036	0.26		
15	rs1051121	0.17		
16	rs491152	0.16		
17	rs1729376	0.16		
18	rs2071231	0.16		

## 4 Discussion

Based on direct comparisons from pairwise meta-analysis and added indirect comparisons, our study employed network meta-analysis to compare the associations between single-nucleotide polymorphisms and skin cancer using the allele model and the dominant model. Our network meta-analysis identified two subgroups in each genetic model, respectively. We ranked SNPs based on their *P*-scores to select the most appropriate SNPs. Our results showed that the minor alleles (T) of rs2228570 (FokI) and (C) of rs13181 (ERCC2) were the highest-ranking SNPs in both subgroups one and two of the allele model. On the other hand, using the dominant model, the homozygous dominant genotype and heterozygote genotype (AA + AT) of rs475007 in subgroup one and the homozygous dominant genotype (AA) of rs238406 in subgroup two were most likely to be associated with skin cancer.

The single-nucleotide polymorphism rs2228570 (FokI) is in the vitamin D receptor (VDR) gene. It is one of the common human VDR SNPs along with rs1544410 (BsmI), rs7975232 (ApaI), and rs731236 (TaqI). Vitamin D is metabolized to vitamin D: 1,25(OH)2D3.1 in response to ultraviolet B (UVB) radiation. This metabolite is the ligand of the VDR, which in turn initiates a series of biological responses in bone metabolism, immunity, cell proliferation, and differentiation by binding to vitamin D response elements in DNA (79). Hence, rs2228570 has not only been associated with various skin diseases, such as chronic spontaneous urticaria (CSU) (80), atopic dermatitis (AD) (81), and leprosy (82), but has also been linked to an increased incidence risk and worse prognosis of different cancers, such as breast cancer (83), ovarian cancer (84), gastric cancer (85), hepatocellular carcinoma (86), papillary thyroid cancer (87), pancreatic cancer (88), and melanoma. Our results are consistent with previous studies using assay methods (89). For instance, the study results of Zeljic et al., who used the assay method, showed that the homozygous recessive genotype of rs2228570 was related to increased melanoma risk compared to the homozygous dominant genotype in the Caucasian population (89). However, no association was observed between rs2228570 and melanoma in this investigation using the biosystem assay method (90).

SNPs rs13181 and rs238406 ranked first and second in subgroups two in both the allele and dominant models. Both SNPs are in the ERCC2 (formerly called XPD) gene. The ERCC2 polymorphisms have ATP-dependent DNA helicase activity, which may impact DNA repair functions. Deficiency of ERCC2 has been reported to lead to xeroderma pigmentosum (XP), trichothiodystrophy (TTD), and Cockayne’s syndrome (CS) (91). This observation may explain why rs13181 and rs238406 were found to be linked to cancers such as lung cancer (92), cervical cancer (93), breast cancer, squamous cell carcinomas of the head and neck (94), and bladder cancer (95). In line with these findings, our results showed that the alternative allele (C) of rs13181 and the homozygous recessive genotype (AA) of rs238406 were significantly associated with SC risk. The study by Kertatbs et al. reported a high frequency of the wild-type allele of rs13181 in advanced melanoma (96). However, an investigation using the microarray chip method, including 1,391 NMSC cases and 2,586 cancer-free controls, did not find significantly increased risks of NMSC for the homozygous dominant genotype of rs13181 (97). Furthermore, a meta-analysis found that the homozygous recessive genotype (AA) of rs238406 was positively associated with an increased risk of cancer of the nervous system, the digestive tract, the genito-urinary system, and the respiratory system, but not basal cell cancer (98).

Matrix metalloproteinases (MMPs) are a family of proteolytic enzymes that are involved in cell mobility, proliferation, differentiation, and apoptosis by degrading extracellular proteins (99). MMP1, a secreted enzyme that cleaves fibrillar collagen, has been linked to cancer by promoting cancer cell proliferation, tumor angiogenesis, and vasculogenesis (100). In the dominant model of our research, all the SNPs in subgroup one were located in the MMP1 gene. The SNP most likely to be associated with SC was rs475007. Furthermore, the homozygous recessive genotype of rs475007 was found to decrease the risk of skin cancer. Similar results were found in Liu’s study, which reported that patients with homozygous dominant genotype and heterozygote genotype for the reference allele of rs475007 were more likely to have larger skin tumors (101).

## 5 Limitation

Due to technical differences and differences in sensitivity, our analysis only included studies that used the PCR genotypic detection method and excluded microarray detection or genome-wide association studies (GWAS). However, GWAS allows for much larger sample sizes than PCR studies. Additionally, due to the limitations of the RStudio and StataSE software and the complexity of multi-arm studies, SNPs only reported in one single article were not included in the final network meta-analysis.

## 6 Future prospective

Our article indicated that people with mutations in the genes FokI (rs2228570), ERCC2 (rs13181), MMP1 (rs475007), and ERCC2 (rs238406) were more likely to have skin cancer. Dysplastic nevi (also called atypical moles) are precursors and risk factors for malignant melanoma (102). However, it is difficult to distinguish them from melanomas because of overlapping features and a lack of predictive markers (103). Thus, our results may provide a possibility for the early detection of asymptomatic skin cancer if routine genetic screening is implemented in the general population in the future. Additionally, the results of our study may also provide valuable information for decision-making when determining the best mode of therapy for SC in a patient. For instance, since FokI is a vitamin D receptor gene and vitamin D is considered to be a protective factor in certain cancers, such as skin cancer (104, 105), supplementation with vitamin D may be used as adjuvant therapy in cancer patients. Therefore, identification of SC patients with FokI gene (rs2228570) mutations is important, since these patients would not benefit from adjuvant Vitamin D therapy.

In addition, we obtained direct and indirect evidence between the SNP pairs through network analysis, which proposed the possibility of hitherto unexplored relationships between certain gene mutations. For example, ERCC2 gene mutations have been shown to indirectly increase the risk of SC (106, 107), and the Melanocortin receptor 1 (MC1R), which encodes melanocyte-stimulating hormone (MSH) receptors, has also been shown to be a risk factor for skin cancer (108). However, surprisingly, indirect evidence from our network meta-analysis showed that ERCC2 (rs13181) was negatively related to MC1R (1805006, 1805007, 1805008, and rs885479) (**Supplementary Table 4**). Therefore, the relationship between ERCC2 and MC1R necessitates further research to determine their roles in SC development.

Finally, as an added scientific value, we applied an innovative research design by performing a network analysis of case–control studies, thus providing a fresh perspective on the NMA method. Our analysis implies that all studies involving genetically-related diseases, whether cohort or case–control studies, can be used to build a network in the meta-analysis, which may then provide valuable information for the diseases’ early detection, diagnosis, staging, treatment, and prognosis.

## Data availability statement

The raw data supporting the conclusions of this article will be made available by the authors, without undue reservation.

## Author contributions

Conceptualization: LZ, HL, and IK. Methodology: LZ and HL. Data curation: LZ, YS, HA, and JZ. Statistical analysis: LZ and HL. Writing (original draft preparation): LZ and IK. Review and editing: ÉP, JM, YS, HA, JZ, HL, and IK. Visualization: ZL. Supervision: HL and IK. Project administration: LZ and IK. All authors listed have made a substantial, direct, and intellectual contribution to the work and approved it for publication.
