# Supplementary material for: The relationship between single nucleotide polymorphisms and skin cancer susceptibility: A systematic review and network meta-analysis
Source: Front Oncol. 2023 Feb 15;13:1094309. doi: 10.3389/fonc.2023.1094309 (PMC9975575; doi:10.3389/fonc.2023.1094309)
Supplement: Supplementary file 1 [file Table_1.docx]

Table 1 Crude associations between the SNPs and skin cancer risk

| SNP | SNP-ID | Author | Study-Code | Alleles model (A vs. B) | | | | Dominant model (AA+AB vs. BB) | | | | Recessive model (AA vs. AB+BB) | | | |
| --- | --- | --- | --- | --- | --- | --- | --- | --- | --- | --- | --- | --- | --- | --- | --- |
|  |  |  |  | OR | 95% CI | | W (%) | OR | 95% CI | | W (%) | OR | 95% CI | | W (%) |
|  |  |  |  |  | L | H |  |  | L | H |  |  | L | H |  |
| rs1015362 | 1 | Helsing P | 22 | 1.02 | 0.80 | 1.29 | 0.21 | 0.90 | 0.46 | 1.78 | 0.12 | 1.04 | 0.78 | 1.39 | 0.25 |
|  |  | Maccioni L | 23 | 1.02 | 0.88 | 1.19 | 0.52 | 1.21 | 0.82 | 1.77 | 0.32 | 0.99 | 0.81 | 1.20 | 0.60 |
| rs1042522 | 2 | Thunell LK | 10 | 0.92 | 0.74 | 1.16 | 0.25 | 0.83 | 0.49 | 1.40 | 0.20 | 0.93 | 0.70 | 1.23 | 0.29 |
|  |  | Ozola A | 15 | 1.27 | 0.97 | 1.67 | 0.14 | 1.43 | 0.79 | 2.61 | 0.12 | 1.33 | 0.92 | 1.91 | 0.15 |
|  |  | Rizzato C | 27 | 1.01 | 0.83 | 1.23 | 0.31 | 1.16 | 0.74 | 1.80 | 0.25 | 0.97 | 0.76 | 1.25 | 0.37 |
|  |  | Povey JE | 30 | 1.11 | 0.90 | 1.36 | 0.28 | 1.07 | 0.68 | 1.69 | 0.24 | 1.15 | 0.89 | 1.48 | 0.31 |
|  |  | Oliveira C | 33 | 1.48 | 1.03 | 2.13 | 0.08 | 1.30 | 0.57 | 2.97 | 0.07 | 1.74 | 1.09 | 2.77 | 0.08 |
|  |  | Nan H | 34 | 0.85 | 0.73 | 1.00 | 0.52 | 0.77 | 0.51 | 1.15 | 0.36 | 0.83 | 0.68 | 1.01 | 0.62 |
|  |  | Oliveira C | 35 | 1.70 | 1.29 | 2.26 | 0.12 | 1.11 | 0.58 | 2.10 | 0.12 | 2.48 | 1.71 | 3.60 | 0.10 |
|  |  | Francisco G | 36 | 1.15 | 0.86 | 1.53 | 0.14 | 1.28 | 0.73 | 2.24 | 0.15 | 1.14 | 0.76 | 1.69 | 0.13 |
|  |  | Capasso M | 48 | 0.96 | 0.75 | 1.23 | 0.21 | 0.95 | 0.62 | 1.46 | 0.29 | 0.95 | 0.67 | 1.36 | 0.18 |
|  |  | Almquist LM | 54 | 1.00 | 0.86 | 1.15 | 0.59 | 0.95 | 0.66 | 1.36 | 0.41 | 1.01 | 0.84 | 1.20 | 0.70 |
| rs1052133 | 3 | Hsu L-I | 7 | 0.82 | 0.55 | 1.22 | 0.08 | 0.82 | 0.36 | 1.86 | 0.08 | 0.74 | 0.41 | 1.31 | 0.08 |
|  |  | Santonocito C | 24 | 0.77 | 0.50 | 1.18 | 0.08 | 0.14 | 0.02 | 1.13 | 0.05 | 0.90 | 0.54 | 1.50 | 0.09 |
|  |  | Povey JE | 30 | 0.92 | 0.72 | 1.19 | 0.19 | 1.52 | 0.69 | 3.32 | 0.07 | 0.84 | 0.62 | 1.14 | 0.26 |
| rs2228570 | 4 | Pena-Chilet M | 21 | 0.98 | 0.79 | 1.21 | 0.27 | 1.10 | 0.71 | 1.70 | 0.26 | 0.93 | 0.70 | 1.23 | 0.28 |
|  |  | Lesiak A | 46 | 0.29 | 0.20 | 0.41 | 0.19 | 0.29 | 0.16 | 0.54 | 0.27 | 0.18 | 0.10 | 0.30 | 0.19 |
|  |  | Li C | 55 | 0.91 | 0.79 | 1.05 | 0.64 | 1.07 | 0.79 | 1.45 | 0.55 | 0.80 | 0.66 | 0.98 | 0.62 |
|  |  | Aristizabal-Pachon A | 59 | 0.50 | 0.35 | 0.73 | 0.12 | 1.42 | 0.62 | 3.22 | 0.06 | 0.20 | 0.11 | 0.36 | 0.15 |
| rs1110400 | 5 | Ozola A | 15 | 1.41 | 0.69 | 2.89 | 0.02 | 1.14 | 0.07 | 18.32 | 0.01 | 1.53 | 0.73 | 3.23 | 0.03 |
|  |  | Helsing P | 22 | 0.50 | 0.19 | 1.36 | 0.02 | 0.93 | 0.06 | 14.86 | 0.01 | 0.50 | 0.18 | 1.36 | 0.03 |
| rs1126809 | 6 | Reis LB | 2 | 1.04 | 0.68 | 1.58 | 0.07 | 1.81 | 0.44 | 7.42 | 0.02 | 0.97 | 0.59 | 1.60 | 0.09 |
|  |  | Helsing P | 22 | 0.92 | 0.74 | 1.14 | 0.27 | 1.03 | 0.60 | 1.77 | 0.17 | 0.86 | 0.65 | 1.14 | 0.31 |
|  |  | Fernandez LP | 29 | 1.00 | 0.71 | 1.41 | 0.10 | 1.01 | 0.44 | 2.34 | 0.07 | 1.01 | 0.66 | 1.55 | 0.12 |
| rs1130409 | 7 | Figl A | 14 | 1.05 | 0.94 | 1.17 | 0.95 | 1.15 | 0.94 | 1.39 | 1.26 | 1.00 | 0.84 | 1.20 | 0.69 |
|  | 7 | Santonocito C | 24 | 1.30 | 0.91 | 1.86 | 0.08 | 1.13 | 0.60 | 2.15 | 0.12 | 1.66 | 0.96 | 2.85 | 0.06 |
| rs1136410 | 8 | Santonocito C | 24 | 0.41 | 0.24 | 0.69 | 0.08 | 0.20 | 0.03 | 1.63 | 0.04 | 0.40 | 0.23 | 0.71 | 0.11 |
|  | 8 | Li C | 32 | 1.16 | 0.93 | 1.44 | 0.24 | 0.94 | 0.48 | 1.84 | 0.12 | 1.22 | 0.95 | 1.56 | 0.33 |
| rs1144393 | 9 | Debniak T | 9 | 0.99 | 0.79 | 1.26 | 0.22 | 1.97 | 1.34 | 2.91 | 0.25 | 1.24 | 0.88 | 1.75 | 0.17 |
|  | 9 | Wang L-E | 47 | 1.13 | 0.99 | 1.30 | 0.61 | 1.29 | 0.99 | 1.68 | 0.64 | 1.12 | 0.92 | 1.36 | 0.55 |
| rs11515 | 10 | Maccioni L | 11 | 1.01 | 0.85 | 1.20 | 0.40 | 1.05 | 0.59 | 1.85 | 0.16 | 1.00 | 0.82 | 1.22 | 0.56 |
|  | 10 | Francisco G | 36 | 0.94 | 0.69 | 1.28 | 0.13 | 0.62 | 0.23 | 1.65 | 0.07 | 0.98 | 0.66 | 1.45 | 0.14 |
|  | 10 | Pjanova D | 56 | 0.96 | 0.56 | 1.66 | 0.04 | 1.23 | 0.11 | 13.75 | 0.01 | 0.94 | 0.52 | 1.70 | 0.07 |
| rs11615 | 11 | Gao R | 25 | 1.69 | 1.19 | 2.42 | 0.07 | 2.19 | 1.11 | 4.33 | 0.07 | 1.91 | 1.14 | 3.21 | 0.06 |
|  | 11 | Povey JE | 30 | 0.99 | 0.82 | 1.21 | 0.33 | 1.25 | 0.86 | 1.80 | 0.34 | 0.89 | 0.69 | 1.15 | 0.36 |
| rs13181 | 12 | Applebaum KM | 8 | 1.07 | 0.96 | 1.19 | 1.03 | 1.04 | 0.88 | 1.24 | 1.67 | 1.12 | 0.95 | 1.32 | 0.81 |
|  | 12 | Figl A | 14 | 0.88 | 0.79 | 0.99 | 0.96 | 0.96 | 0.75 | 1.22 | 0.92 | 0.80 | 0.68 | 0.94 | 0.95 |
|  | 12 | Povey JE | 30 | 1.00 | 0.83 | 1.20 | 0.35 | 1.00 | 0.69 | 1.45 | 0.38 | 1.00 | 0.78 | 1.28 | 0.36 |
|  | 12 | Li C | 31 | 0.86 | 0.73 | 1.01 | 0.48 | 0.89 | 0.64 | 1.24 | 0.50 | 0.78 | 0.62 | 0.98 | 0.47 |
| rs1544410 | 13 | Morgado-Aguila C | 38 | 1.22 | 0.77 | 1.93 | 0.05 | 0.93 | 0.39 | 2.22 | 0.07 | 1.59 | 0.82 | 3.11 | 0.04 |
|  | 13 | Burns EM | 42 | 0.65 | 0.43 | 0.97 | 0.09 | 0.64 | 0.31 | 1.33 | 0.12 | 0.54 | 0.30 | 0.97 | 0.09 |
|  | 13 | Lesiak A | 46 | 1.80 | 1.34 | 2.42 | 0.11 | 1.00 | 0.59 | 1.68 | 0.19 | 4.82 | 2.95 | 7.88 | 0.04 |
|  | 13 | Li C | 55 | 1.17 | 1.02 | 1.34 | 0.59 | 1.08 | 0.83 | 1.39 | 0.75 | 1.33 | 1.08 | 1.63 | 0.46 |
| rs16891982 | 14 | Reis LB | 2 | 2.94 | 1.67 | 5.19 | 0.02 | 9.52 | 1.20 | 75.51 | 0.01 | 2.74 | 1.46 | 5.13 | 0.04 |
|  | 14 | Guedj M | 28 | 2.76 | 2.12 | 3.58 | 0.13 | 2.73 | 1.02 | 7.30 | 0.04 | 2.93 | 2.22 | 3.87 | 0.20 |
|  | 14 | Fernandez LP | 29 | 2.49 | 1.45 | 4.26 | 0.03 | 3.82 | 0.47 | 31.42 | 0.01 | 2.63 | 1.47 | 4.70 | 0.05 |
| rs1695 | 15 | Hsu L-I | 7 | 0.95 | 0.58 | 1.56 | 0.05 | 2.77 | 0.34 | 22.56 | 0.01 | 0.79 | 0.45 | 1.40 | 0.08 |
|  | 15 | Ibarrola-Villava M | 12 | 0.76 | 0.62 | 0.94 | 0.32 | 0.64 | 0.40 | 1.05 | 0.29 | 0.72 | 0.55 | 0.95 | 0.35 |
| rs17655 | 16 | Figl A | 14 | 0.93 | 0.82 | 1.06 | 0.74 | 0.94 | 0.68 | 1.30 | 0.51 | 0.91 | 0.77 | 1.07 | 0.90 |
|  | 16 | Povey JE | 30 | 1.19 | 0.96 | 1.48 | 0.24 | 1.31 | 0.75 | 2.31 | 0.14 | 1.22 | 0.94 | 1.58 | 0.29 |
|  | 16 | Li C | 31 | 1.04 | 0.85 | 1.26 | 0.31 | 1.18 | 0.67 | 2.08 | 0.15 | 1.03 | 0.81 | 1.29 | 0.40 |
| rs179008 | 17 | Russo I | 43 | 1.03 | 0.61 | 1.73 | 0.04 | 0.22 | 0.04 | 1.08 | 0.05 | 1.41 | 0.75 | 2.66 | 0.05 |
|  | 17 | Elefanti L | 53 | 0.71 | 0.42 | 1.21 | 0.05 | 0.54 | 0.11 | 2.62 | 0.03 | 0.69 | 0.37 | 1.28 | 0.07 |
| rs1799793 | 18 | Applebaum KM | 8 | 1.08 | 0.97 | 1.21 | 0.98 | 1.05 | 0.88 | 1.26 | 1.60 | 1.15 | 0.97 | 1.35 | 0.75 |
|  | 18 | Figl A | 14 | 1.00 | 0.89 | 1.12 | 0.94 | 1.00 | 0.79 | 1.26 | 0.96 | 1.00 | 0.85 | 1.17 | 0.88 |
|  | 18 | Li Y-L | 18 | 0.81 | 0.68 | 0.96 | 0.46 | 0.72 | 0.49 | 1.06 | 0.41 | 0.79 | 0.64 | 0.98 | 0.53 |
|  | 18 | Li C | 31 | 0.90 | 0.76 | 1.06 | 0.46 | 1.01 | 0.71 | 1.44 | 0.41 | 0.81 | 0.65 | 1.02 | 0.47 |
|  | 18 | Vogel U | 51 | 1.04 | 0.83 | 1.31 | 0.22 | 1.37 | 0.85 | 2.22 | 0.19 | 0.94 | 0.69 | 1.29 | 0.23 |
| rs1799801 | 19 | Povey JE | 30 | 0.91 | 0.75 | 1.11 | 0.33 | 1.41 | 0.89 | 2.22 | 0.21 | 0.77 | 0.59 | 0.99 | 0.39 |
|  | 19 | Oliveira C | 33 | 0.97 | 0.68 | 1.38 | 0.10 | 0.84 | 0.38 | 1.89 | 0.09 | 1.00 | 0.63 | 1.58 | 0.10 |
| rs1800419 | 20 | Fernandez LP | 29 | 0.99 | 0.73 | 1.34 | 0.14 | 0.99 | 0.59 | 1.66 | 0.19 | 1.00 | 0.63 | 1.60 | 0.10 |
|  | 20 | Jannot A-S | 58 | 0.83 | 0.57 | 1.21 | 0.09 | 1.04 | 0.55 | 1.98 | 0.12 | 0.56 | 0.30 | 1.07 | 0.07 |
| rs1800872 | 21 | Rizzato C | 26 | 1.05 | 0.86 | 1.28 | 0.30 | 1.06 | 0.61 | 1.86 | 0.16 | 1.07 | 0.84 | 1.37 | 0.35 |
|  | 21 | Schoof N | 37 | 0.74 | 0.52 | 1.06 | 0.11 | 0.48 | 0.20 | 1.16 | 0.10 | 0.78 | 0.50 | 1.20 | 0.13 |
| rs1800896 | 22 | Rizzato C | 26 | 0.91 | 0.76 | 1.09 | 0.39 | 0.78 | 0.57 | 1.08 | 0.56 | 0.96 | 0.73 | 1.26 | 0.31 |
|  | 22 | Schoof N | 37 | 1.13 | 0.83 | 1.53 | 0.12 | 0.92 | 0.54 | 1.56 | 0.19 | 1.45 | 0.89 | 2.36 | 0.08 |
| rs1805005 | 23 | Cordoba-Lanus E | 4 | 1.12 | 0.86 | 1.45 | 0.17 | 0.59 | 0.19 | 1.82 | 0.05 | 1.19 | 0.89 | 1.58 | 0.24 |
|  | 23 | Helsing P | 22 | 1.22 | 0.85 | 1.74 | 0.09 | 1.89 | 0.17 | 20.90 | 0.01 | 1.23 | 0.84 | 1.79 | 0.14 |
| rs1805006 | 24 | Cordoba-Lanus E | 4 | 3.10 | 1.01 | 9.55 | 0.01 | 0.95 | 0.06 | 15.18 | 0.01 | 3.13 | 1.01 | 9.67 | 0.01 |
|  | 24 | Helsing P | 22 | 0.17 | 0.06 | 0.51 | 0.03 | 0.93 | 0.06 | 14.93 | 0.01 | 0.17 | 0.06 | 0.50 | 0.06 |
| rs1805007 | 25 | Motorina AV | 3 | 5.83 | 2.65 | 12.80 | 0.01 | 3.71 | 0.23 | 59.87 | 0.00 | 6.36 | 2.83 | 14.27 | 0.01 |
|  | 25 | Cordoba-Lanus E | 4 | 2.71 | 1.63 | 4.50 | 0.03 | 1.90 | 0.17 | 20.99 | 0.01 | 2.71 | 1.61 | 4.55 | 0.06 |
|  | 25 | Ozola A | 15 | 0.45 | 0.25 | 0.81 | 0.06 | 0.57 | 0.05 | 6.32 | 0.01 | 0.46 | 0.25 | 0.83 | 0.10 |
|  | 25 | Helsing P | 22 | 0.56 | 0.42 | 0.74 | 0.22 | 0.25 | 0.09 | 0.68 | 0.12 | 0.56 | 0.41 | 0.77 | 0.30 |
| rs1805008 | 26 | Motorina AV | 3 | 3.73 | 0.23 | 59.89 | 0.00 | 3.73 | 0.23 | 60.19 | 0.00 | 3.73 | 0.23 | 60.19 | 0.00 |
|  | 26 | Cordoba-Lanus E | 4 | 3.10 | 1.32 | 7.26 | 0.01 | 0.93 | 0.06 | 14.90 | 0.01 | 3.15 | 1.34 | 7.41 | 0.02 |
|  | 26 | Ozola A | 15 | 0.67 | 0.44 | 1.02 | 0.09 | 0.85 | 0.19 | 3.83 | 0.02 | 0.84 | 0.55 | 1.29 | 0.13 |
|  | 26 | Helsing P | 22 | 0.84 | 0.63 | 1.11 | 0.16 | 0.93 | 0.30 | 2.91 | 0.04 | 0.81 | 0.59 | 1.11 | 0.24 |
| rs1805009 | 27 | Motorina AV | 3 | 3.64 | 0.23 | 58.55 | 0.00 | 3.63 | 0.23 | 58.60 | 0.00 | 3.66 | 0.23 | 59.09 | 0.00 |
|  | 27 | Helsing P | 22 | 0.55 | 0.27 | 1.13 | 0.03 | 0.92 | 0.06 | 14.82 | 0.01 | 0.54 | 0.26 | 1.12 | 0.06 |
| rs20417 | 28 | Gomez-Lira M | 19 | 1.33 | 1.01 | 1.75 | 0.14 | 1.62 | 0.83 | 3.19 | 0.09 | 1.35 | 0.96 | 1.89 | 0.17 |
|  | 28 | Cocos R | 45 | 1.07 | 0.74 | 1.56 | 0.09 | 0.99 | 0.41 | 2.39 | 0.07 | 1.11 | 0.71 | 1.74 | 0.10 |
| rs2227981 | 29 | Fathi F | 1 | 0.88 | 0.68 | 1.14 | 0.19 | 0.76 | 0.45 | 1.28 | 0.22 | 0.90 | 0.64 | 1.28 | 0.19 |
|  | 29 | Gomez GVB | 5 | 1.00 | 0.78 | 1.29 | 0.19 | 0.94 | 0.57 | 1.54 | 0.21 | 1.04 | 0.72 | 1.50 | 0.16 |
| rs2228000 | 30 | Figl A | 14 | 0.99 | 0.87 | 1.12 | 0.77 | 0.99 | 0.72 | 1.35 | 0.53 | 0.99 | 0.84 | 1.16 | 0.88 |
|  | 30 | Li C | 31 | 1.03 | 0.86 | 1.24 | 0.37 | 0.72 | 0.46 | 1.12 | 0.31 | 1.15 | 0.92 | 1.44 | 0.40 |
| rs2228001 | 31 | Figl A | 14 | 1.01 | 0.90 | 1.13 | 0.94 | 0.98 | 0.79 | 1.21 | 1.16 | 1.03 | 0.87 | 1.22 | 0.80 |
|  | 31 | Li C | 31 | 1.10 | 0.93 | 1.29 | 0.44 | 0.99 | 0.73 | 1.34 | 0.55 | 1.23 | 0.97 | 1.56 | 0.35 |
|  | 31 | Oliveira C | 33 | 0.77 | 0.55 | 1.09 | 0.12 | 0.41 | 0.19 | 0.91 | 0.14 | 0.87 | 0.55 | 1.38 | 0.11 |
| rs2228479 | 32 | Ozola A | 15 | 0.72 | 0.47 | 1.10 | 0.08 | 0.57 | 0.10 | 3.13 | 0.02 | 0.70 | 0.44 | 1.10 | 0.13 |
|  | 32 | Helsing P | 22 | 0.87 | 0.61 | 1.23 | 0.11 | 0.37 | 0.07 | 1.91 | 0.03 | 0.90 | 0.62 | 1.31 | 0.17 |
| rs2279744 | 33 | Ozola A | 15 | 0.84 | 0.63 | 1.11 | 0.17 | 0.76 | 0.41 | 1.43 | 0.15 | 0.81 | 0.56 | 1.17 | 0.19 |
|  | 33 | Nan H | 34 | 0.92 | 0.80 | 1.06 | 0.62 | 0.90 | 0.68 | 1.18 | 0.70 | 0.91 | 0.75 | 1.10 | 0.61 |
|  | 33 | Oliveira C | 35 | 1.04 | 0.80 | 1.36 | 0.17 | 0.82 | 0.46 | 1.46 | 0.17 | 1.18 | 0.81 | 1.73 | 0.14 |
|  | 33 | Capasso M | 48 | 0.95 | 0.73 | 1.24 | 0.18 | 0.62 | 0.35 | 1.09 | 0.20 | 1.10 | 0.78 | 1.55 | 0.18 |
|  | 33 | Wilkening S | 49 | 1.14 | 0.95 | 1.36 | 0.35 | 1.12 | 0.78 | 1.62 | 0.36 | 1.21 | 0.94 | 1.56 | 0.32 |
|  | 33 | Almquist LM | 54 | 0.87 | 0.76 | 1.01 | 0.67 | 0.84 | 0.62 | 1.14 | 0.63 | 0.84 | 0.70 | 1.02 | 0.69 |
| rs238406 | 34 | Hsu L-I | 7 | 0.71 | 0.48 | 1.06 | 0.09 | 0.74 | 0.39 | 1.41 | 0.14 | 0.49 | 0.23 | 1.03 | 0.07 |
|  | 34 | Povey JE | 30 | 0.98 | 0.82 | 1.18 | 0.38 | 0.97 | 0.71 | 1.32 | 0.54 | 0.98 | 0.74 | 1.31 | 0.27 |
|  | 34 | Vogel U | 51 | 0.84 | 0.68 | 1.05 | 0.28 | 0.68 | 0.47 | 0.98 | 0.48 | 0.92 | 0.65 | 1.31 | 0.19 |
| rs25487 | 35 | Hsu L-I | 7 | 0.86 | 0.57 | 1.32 | 0.07 | 0.87 | 0.37 | 2.05 | 0.07 | 0.83 | 0.48 | 1.43 | 0.08 |
|  | 35 | Figl A | 14 | 0.95 | 0.84 | 1.06 | 0.92 | 0.93 | 0.73 | 1.18 | 0.95 | 0.93 | 0.79 | 1.09 | 0.89 |
|  | 35 | Santonocito C | 24 | 0.29 | 0.20 | 0.44 | 0.15 | 0.05 | 0.01 | 0.22 | 0.23 | 0.32 | 0.19 | 0.54 | 0.14 |
|  | 35 | Povey JE | 30 | 1.00 | 0.83 | 1.21 | 0.35 | 0.99 | 0.70 | 1.42 | 0.41 | 1.01 | 0.77 | 1.31 | 0.32 |
|  | 35 | Li C | 32 | 1.02 | 0.86 | 1.20 | 0.43 | 0.95 | 0.68 | 1.34 | 0.45 | 1.05 | 0.84 | 1.32 | 0.41 |
| rs25489 | 36 | Hsu L-I | 7 | 1.85 | 0.88 | 3.87 | 0.02 | 1.37 | 0.15 | 12.43 | 0.01 | 1.76 | 0.81 | 3.83 | 0.03 |
|  | 36 | Figl A | 14 | 1.20 | 0.94 | 1.52 | 0.20 | 4.62 | 1.55 | 13.77 | 0.03 | 1.09 | 0.85 | 1.41 | 0.33 |
|  | 36 | Santonocito C | 24 | 0.34 | 0.21 | 0.56 | 0.10 | 0.06 | 0.01 | 0.48 | 0.11 | 0.39 | 0.22 | 0.68 | 0.12 |
| rs2910164 | 37 | Yamashita J | 44 | 1.42 | 0.85 | 2.35 | 0.04 | 0.69 | 0.07 | 6.83 | 0.01 | 2.13 | 1.04 | 4.34 | 0.03 |
|  | 37 | Sangalli A | 52 | 0.91 | 0.71 | 1.17 | 0.21 | 0.77 | 0.43 | 1.39 | 0.17 | 0.92 | 0.67 | 1.27 | 0.23 |
| rs3088440 | 38 | Maccioni L | 11 | 0.67 | 0.51 | 0.87 | 0.21 | 0.68 | 0.20 | 2.36 | 0.04 | 0.65 | 0.49 | 0.86 | 0.35 |
|  | 38 | Pjanova D | 56 | 0.67 | 0.38 | 1.17 | 0.05 | 0.62 | 0.04 | 9.93 | 0.01 | 0.64 | 0.35 | 1.16 | 0.08 |
| rs36084323 | 39 | Gomez GVB | 5 | 0.83 | 0.48 | 1.42 | 0.05 | 0.16 | 0.02 | 1.36 | 0.04 | 1.05 | 0.58 | 1.89 | 0.06 |
|  | 39 | Fathi F | 6 | 1.00 | 0.71 | 1.42 | 0.10 | 0.86 | 0.36 | 2.07 | 0.07 | 1.04 | 0.69 | 1.56 | 0.13 |
| rs3731249 | 40 | Tovar-Parra JD | 39 | 0.82 | 0.26 | 2.53 | 0.01 | 0.51 | 0.03 | 8.29 | 0.01 | 0.81 | 0.26 | 2.56 | 0.02 |
|  | 40 | Pjanova D | 56 | 0.23 | 0.06 | 0.86 | 0.02 | 0.62 | 0.04 | 9.93 | 0.01 | 0.22 | 0.06 | 0.84 | 0.03 |
| rs4516035 | 41 | Pena-Chilet M | 21 | 0.95 | 0.77 | 1.16 | 0.30 | 0.77 | 0.52 | 1.14 | 0.40 | 1.04 | 0.77 | 1.40 | 0.24 |
|  | 41 | Povey JE | 30 | 1.11 | 0.92 | 1.32 | 0.36 | 1.27 | 0.92 | 1.74 | 0.45 | 1.06 | 0.81 | 1.39 | 0.30 |
| rs475007 | 42 | Debniak T | 9 | 1.08 | 0.85 | 1.36 | 0.22 | 1.64 | 1.12 | 2.40 | 0.28 | 1.25 | 0.88 | 1.77 | 0.16 |
|  | 42 | Wang L-E | 47 | 0.92 | 0.81 | 1.05 | 0.71 | 0.89 | 0.71 | 1.12 | 1.01 | 0.90 | 0.74 | 1.11 | 0.55 |
| rs4911414 | 43 | Helsing P | 22 | 0.82 | 0.67 | 1.02 | 0.31 | 0.80 | 0.51 | 1.26 | 0.28 | 0.77 | 0.58 | 1.02 | 0.32 |
|  | 43 | Maccioni L | 23 | 0.86 | 0.74 | 1.00 | 0.58 | 0.98 | 0.69 | 1.39 | 0.42 | 0.78 | 0.65 | 0.95 | 0.68 |
| rs494379 | 44 | Debniak T | 9 | 1.16 | 0.87 | 1.53 | 0.14 | 11.72 | 6.72 | 20.43 | 0.07 | 2.64 | 1.94 | 3.58 | 0.14 |
|  | 44 | Wang L-E | 47 | 0.86 | 0.73 | 1.01 | 0.50 | 0.67 | 0.44 | 1.04 | 0.34 | 0.88 | 0.72 | 1.06 | 0.62 |
| rs498186 | 45 | Debniak T | 9 | 1.03 | 0.81 | 1.30 | 0.21 | 2.73 | 1.83 | 4.09 | 0.20 | 1.43 | 1.03 | 1.99 | 0.17 |
|  | 45 | Wang L-E | 47 | 0.97 | 0.85 | 1.11 | 0.68 | 1.03 | 0.81 | 1.31 | 0.88 | 0.92 | 0.75 | 1.13 | 0.57 |
| rs514921 | 46 | Debniak T | 9 | 0.94 | 0.72 | 1.24 | 0.17 | 11.46 | 6.58 | 19.97 | 0.07 | 2.18 | 1.61 | 2.96 | 0.16 |
|  | 46 | Wang L-E | 47 | 0.91 | 0.79 | 1.06 | 0.58 | 0.81 | 0.58 | 1.13 | 0.51 | 0.92 | 0.76 | 1.11 | 0.64 |
| rs731236 | 47 | Pena-Chilet M | 21 | 1.05 | 0.85 | 1.30 | 0.27 | 1.19 | 0.79 | 1.81 | 0.27 | 1.01 | 0.75 | 1.36 | 0.25 |
|  | 47 | Burns EM | 42 | 0.69 | 0.46 | 1.04 | 0.09 | 0.67 | 0.31 | 1.42 | 0.11 | 0.60 | 0.34 | 1.07 | 0.09 |
|  | 47 | Lesiak A | 46 | 0.55 | 0.39 | 0.77 | 0.14 | 0.62 | 0.35 | 1.11 | 0.19 | 0.40 | 0.24 | 0.66 | 0.14 |
|  | 47 | Li C | 55 | 1.28 | 1.12 | 1.48 | 0.55 | 1.24 | 0.95 | 1.61 | 0.68 | 1.48 | 1.21 | 1.81 | 0.45 |
|  | 47 | Aristizabal-Pachon A | 59 | 3.13 | 1.65 | 5.94 | 0.02 | 1.00 | 0.06 | 16.17 | 0.01 | 3.65 | 1.86 | 7.17 | 0.03 |
| rs7975232 | 48 | Morgado-Aguila C | 38 | 0.92 | 0.59 | 1.44 | 0.06 | 0.62 | 0.28 | 1.35 | 0.11 | 1.23 | 0.59 | 2.55 | 0.04 |
|  | 48 | Burns EM | 42 | 1.47 | 0.98 | 2.19 | 0.06 | 1.11 | 0.57 | 2.17 | 0.11 | 2.21 | 1.19 | 4.08 | 0.04 |
|  | 48 | Lesiak A | 46 | 0.79 | 0.56 | 1.09 | 0.13 | 1.08 | 0.63 | 1.85 | 0.17 | 0.55 | 0.33 | 0.90 | 0.12 |
| rs861539 | 49 | Figl A | 14 | 0.94 | 0.84 | 1.06 | 0.96 | 1.08 | 0.87 | 1.35 | 1.04 | 0.85 | 0.72 | 1.00 | 0.88 |
|  | 49 | Povey JE | 30 | 1.14 | 0.94 | 1.38 | 0.32 | 1.05 | 0.71 | 1.54 | 0.34 | 1.25 | 0.97 | 1.62 | 0.29 |
| rs885479 | 50 | Cordoba-Lanus E | 4 | 4.66 | 2.26 | 9.61 | 0.01 | 0.94 | 0.06 | 15.12 | 0.01 | 4.83 | 2.33 | 10.02 | 0.02 |
|  | 50 | Helsing P | 22 | 0.94 | 0.65 | 1.38 | 0.09 | 0.62 | 0.10 | 3.72 | 0.02 | 0.96 | 0.64 | 1.44 | 0.14 |
| rs1156882 | 51 | Fathi F | 1 | 1.65 | 1.06 | 2.57 | 0.05 | 1.54 | 0.40 | 6.04 | 0.02 | 1.73 | 1.06 | 2.81 | 0.07 |
| rs1129038 | 52 | Reis LB | 2 | 1.65 | 1.16 | 2.35 | 0.08 | 1.57 | 0.87 | 2.81 | 0.12 | 2.11 | 1.25 | 3.58 | 0.05 |
| rs1426654 | 53 | Reis LB | 2 | 6.02 | 2.06 | 17.56 | 0.01 | 0.89 | 0.06 | 14.36 | 0.01 | 8.43 | 2.47 | 28.79 | 0.01 |
| rs2228478 | 54 | Cordoba-Lanus E | 4 | 0.95 | 0.69 | 1.30 | 0.13 | 0.24 | 0.03 | 2.15 | 0.03 | 0.99 | 0.71 | 1.38 | 0.20 |
| rs2227982 | 55 | Gomez GVB | 5 | 1.00 | 0.57 | 1.77 | 0.04 | 0.50 | 0.05 | 5.51 | 0.01 | 1.10 | 0.61 | 1.99 | 0.06 |
| rs41386349 | 56 | Gomez GVB | 5 | 1.98 | 1.15 | 3.42 | 0.03 | 3.04 | 0.31 | 29.39 | 0.01 | 1.89 | 1.07 | 3.34 | 0.05 |
| rs10204525 | 57 | Fathi F | 6 | 0.87 | 0.60 | 1.26 | 0.09 | 0.84 | 0.32 | 2.23 | 0.06 | 0.85 | 0.56 | 1.31 | 0.13 |
| rs1051740 | 58 | Hsu L-I | 7 | 1.23 | 0.79 | 1.92 | 0.06 | 2.99 | 1.01 | 8.83 | 0.03 | 0.94 | 0.53 | 1.65 | 0.07 |
| rs1800566 | 59 | Hsu L-I | 7 | 0.72 | 0.49 | 1.07 | 0.09 | 0.58 | 0.28 | 1.18 | 0.12 | 0.67 | 0.36 | 1.23 | 0.08 |
| rs2234922 | 60 | Hsu L-I | 7 | 1.32 | 0.72 | 2.42 | 0.03 | 1.35 | 0.15 | 12.26 | 0.01 | 1.26 | 0.65 | 2.41 | 0.05 |
| rs609429 | 61 | Hsu L-I | 7 | 0.96 | 0.63 | 1.46 | 0.07 | 0.98 | 0.46 | 2.06 | 0.09 | 0.94 | 0.51 | 1.72 | 0.06 |
| rs1800975 | 62 | Applebaum KM | 8 | 1.13 | 1.01 | 1.25 | 1.01 | 1.11 | 0.93 | 1.31 | 1.61 | 1.19 | 1.02 | 1.39 | 0.82 |
| rs1051121 | 63 | Debniak T | 9 | 2.68 | 1.03 | 6.95 | 0.01 | 266.22 | 37.09 | 1910.75 | 0.00 | 48.73 | 21.32 | 111.40 | 0.01 |
| rs11225395 | 64 | Debniak T | 9 | 0.74 | 0.59 | 0.93 | 0.26 | 0.71 | 0.46 | 1.10 | 0.33 | 0.64 | 0.46 | 0.91 | 0.23 |
| rs11225426 | 65 | Debniak T | 9 | 1.22 | 0.78 | 1.93 | 0.05 | 123.17 | 30.32 | 500.35 | 0.01 | 7.99 | 5.43 | 11.77 | 0.06 |
| rs1729376 | 66 | Debniak T | 9 | 1.16 | 0.73 | 1.84 | 0.05 | 253.58 | 35.33 | 1820.07 | 0.00 | 8.28 | 5.61 | 12.22 | 0.06 |
| rs2071230 | 67 | Debniak T | 9 | 1.04 | 0.66 | 1.64 | 0.06 | 122.25 | 30.09 | 496.71 | 0.01 | 7.14 | 4.90 | 10.43 | 0.07 |
| rs2071231 | 68 | Debniak T | 9 | 0.98 | 0.39 | 2.42 | 0.01 | 283.23 | 39.47 | 2032.32 | 0.00 | 29.28 | 15.24 | 56.26 | 0.02 |
| rs3213460 | 69 | Debniak T | 9 | 0.97 | 0.69 | 1.37 | 0.10 | 44.51 | 18.06 | 109.71 | 0.02 | 3.97 | 2.88 | 5.47 | 0.11 |
| rs470215 | 70 | Debniak T | 9 | 0.95 | 0.74 | 1.21 | 0.21 | 3.92 | 2.55 | 6.02 | 0.16 | 1.50 | 1.09 | 2.07 | 0.17 |
| rs470358 | 71 | Debniak T | 9 | 1.00 | 0.78 | 1.27 | 0.21 | 2.01 | 1.34 | 2.99 | 0.23 | 1.25 | 0.88 | 1.78 | 0.16 |
| rs491152 | 72 | Debniak T | 9 | 1.07 | 0.07 | 17.15 | 0.00 | 291.00 | 40.56 | 2087.73 | 0.00 | 291.00 | 40.56 | 2087.73 | 0.00 |
| rs5031036 | 73 | Debniak T | 9 | 1.17 | 0.76 | 1.79 | 0.06 | 120.69 | 29.70 | 490.55 | 0.01 | 6.72 | 4.65 | 9.71 | 0.07 |
| rs71250626 | 74 | Debniak T | 9 | 0.82 | 0.63 | 1.08 | 0.19 | 9.92 | 5.75 | 17.10 | 0.08 | 1.81 | 1.33 | 2.48 | 0.17 |
| rs7945189 | 75 | Debniak T | 9 | 0.87 | 0.59 | 1.29 | 0.09 | 119.38 | 29.38 | 485.06 | 0.01 | 4.93 | 3.51 | 6.93 | 0.09 |
| rs996999 | 76 | Debniak T | 9 | 0.99 | 0.75 | 1.31 | 0.16 | 12.76 | 7.21 | 22.59 | 0.07 | 2.39 | 1.77 | 3.25 | 0.15 |
| ﻿rs2279744 | 77 | Thunell LK | 10 | 1.05 | 0.85 | 1.29 | 0.27 | 1.23 | 0.79 | 1.89 | 0.26 | 1.00 | 0.75 | 1.33 | 0.26 |
| rs1563828 | 78 | Thunell LK | 10 | 1.04 | 0.84 | 1.30 | 0.25 | 0.76 | 0.48 | 1.19 | 0.27 | 1.19 | 0.89 | 1.57 | 0.26 |
| rs1011970 | 79 | Maccioni L | 11 | 0.81 | 0.68 | 0.98 | 0.41 | 0.40 | 0.23 | 0.72 | 0.24 | 0.87 | 0.71 | 1.06 | 0.56 |
| rs1063192 | 80 | Maccioni L | 11 | 1.11 | 0.97 | 1.26 | 0.65 | 1.25 | 0.95 | 1.64 | 0.62 | 1.09 | 0.91 | 1.32 | 0.61 |
| rs10757257 | 81 | Maccioni L | 11 | 1.22 | 1.07 | 1.39 | 0.63 | 1.32 | 1.01 | 1.72 | 0.64 | 1.29 | 1.07 | 1.55 | 0.55 |
| rs10811629 | 82 | Maccioni L | 11 | 1.20 | 1.05 | 1.37 | 0.63 | 1.22 | 0.93 | 1.59 | 0.67 | 1.29 | 1.07 | 1.56 | 0.54 |
| rs10811640 | 83 | Maccioni L | 11 | 0.91 | 0.80 | 1.03 | 0.77 | 0.86 | 0.70 | 1.06 | 1.27 | 0.90 | 0.73 | 1.11 | 0.52 |
| rs13298881 | 84 | Maccioni L | 11 | 0.93 | 0.77 | 1.12 | 0.36 | 0.85 | 0.46 | 1.59 | 0.14 | 0.93 | 0.75 | 1.15 | 0.52 |
| rs1335510 | 85 | Maccioni L | 11 | 1.21 | 1.06 | 1.38 | 0.62 | 1.24 | 0.95 | 1.62 | 0.66 | 1.31 | 1.09 | 1.58 | 0.55 |
| rs1341866 | 86 | Maccioni L | 11 | 1.21 | 1.06 | 1.39 | 0.63 | 1.27 | 0.97 | 1.66 | 0.66 | 1.31 | 1.08 | 1.58 | 0.54 |
| rs2218220 | 87 | Maccioni L | 11 | 0.83 | 0.73 | 0.94 | 0.81 | 0.71 | 0.58 | 0.88 | 1.41 | 0.85 | 0.69 | 1.06 | 0.51 |
| rs2518719 | 88 | Maccioni L | 11 | 0.99 | 0.83 | 1.19 | 0.39 | 0.74 | 0.38 | 1.43 | 0.13 | 1.02 | 0.83 | 1.24 | 0.54 |
| rs2811708 | 89 | Maccioni L | 11 | 0.97 | 0.85 | 1.12 | 0.63 | 1.03 | 0.75 | 1.41 | 0.51 | 0.95 | 0.79 | 1.14 | 0.68 |
| rs2811710 | 90 | Maccioni L | 11 | 0.88 | 0.77 | 1.01 | 0.74 | 0.75 | 0.59 | 0.97 | 0.91 | 0.91 | 0.75 | 1.10 | 0.66 |
| rs3217992 | 91 | Maccioni L | 11 | 1.02 | 0.90 | 1.16 | 0.72 | 1.05 | 0.84 | 1.31 | 0.99 | 1.01 | 0.83 | 1.24 | 0.55 |
| rs3218009 | 92 | Maccioni L | 11 | 0.99 | 0.79 | 1.25 | 0.23 | 0.67 | 0.22 | 2.09 | 0.05 | 1.01 | 0.79 | 1.30 | 0.36 |
| rs3218020 | 93 | Maccioni L | 11 | 0.98 | 0.86 | 1.11 | 0.73 | 0.95 | 0.75 | 1.20 | 0.95 | 0.98 | 0.81 | 1.19 | 0.59 |
| rs3731239 | 94 | Maccioni L | 11 | 1.07 | 0.93 | 1.23 | 0.62 | 0.91 | 0.68 | 1.22 | 0.62 | 1.17 | 0.97 | 1.41 | 0.60 |
| rs3731257 | 95 | Maccioni L | 11 | 1.01 | 0.87 | 1.16 | 0.59 | 1.01 | 0.72 | 1.40 | 0.48 | 1.01 | 0.84 | 1.21 | 0.66 |
| rs4636294 | 96 | Maccioni L | 11 | 0.84 | 0.74 | 0.95 | 0.80 | 0.72 | 0.59 | 0.88 | 1.41 | 0.87 | 0.70 | 1.08 | 0.50 |
| rs545226 | 97 | Maccioni L | 11 | 1.00 | 0.88 | 1.14 | 0.73 | 0.96 | 0.78 | 1.19 | 1.15 | 1.03 | 0.84 | 1.27 | 0.51 |
| rs573687 | 98 | Maccioni L | 11 | 1.12 | 0.97 | 1.29 | 0.59 | 1.26 | 0.91 | 1.74 | 0.45 | 1.12 | 0.94 | 1.35 | 0.62 |
| rs7023329 | 99 | Maccioni L | 11 | 1.17 | 1.03 | 1.33 | 0.68 | 1.21 | 0.97 | 1.50 | 1.01 | 1.26 | 1.03 | 1.54 | 0.47 |
| rs751173 | 100 | Maccioni L | 11 | 0.86 | 0.75 | 0.97 | 0.79 | 0.79 | 0.63 | 0.99 | 1.16 | 0.83 | 0.68 | 1.02 | 0.60 |
| rs935053 | 101 | Maccioni L | 11 | 0.82 | 0.72 | 0.93 | 0.81 | 0.69 | 0.56 | 0.85 | 1.42 | 0.84 | 0.68 | 1.04 | 0.53 |
| rs1138272 | 102 | Ibarrola-Villava M | 12 | 0.92 | 0.58 | 1.45 | 0.06 | 1.25 | 0.43 | 3.64 | 0.04 | 0.86 | 0.51 | 1.43 | 0.09 |
| rs1799794 | 103 | Figl A | 14 | 1.09 | 0.95 | 1.25 | 0.63 | 1.14 | 0.78 | 1.67 | 0.33 | 1.11 | 0.94 | 1.30 | 0.80 |
| rs3213245 | 104 | Figl A | 14 | 1.06 | 0.95 | 1.19 | 0.91 | 1.09 | 0.88 | 1.35 | 1.07 | 1.08 | 0.92 | 1.27 | 0.78 |
| rs7141928 | 105 | Figl A | 14 | 0.99 | 0.88 | 1.10 | 0.96 | 1.04 | 0.84 | 1.27 | 1.19 | 0.95 | 0.80 | 1.12 | 0.80 |
| rs861530 | 106 | Figl A | 14 | 1.06 | 0.94 | 1.19 | 0.84 | 1.12 | 0.87 | 1.43 | 0.78 | 1.05 | 0.90 | 1.23 | 0.85 |
| rs258322 | 107 | Ozola A | 15 | 0.53 | 0.33 | 0.86 | 0.08 | 0.31 | 0.03 | 2.80 | 0.02 | 0.51 | 0.31 | 0.85 | 0.13 |
| rs4785763 | 108 | Ozola A | 15 | 0.74 | 0.56 | 0.97 | 0.20 | 0.64 | 0.37 | 1.09 | 0.23 | 0.69 | 0.47 | 1.01 | 0.18 |
| rs2228527 | 109 | Li Y-L | 18 | 0.65 | 0.55 | 0.78 | 0.49 | 0.49 | 0.32 | 0.76 | 0.40 | 0.62 | 0.50 | 0.77 | 0.58 |
| rs2228529 | 110 | Li Y-L | 18 | 0.63 | 0.53 | 0.74 | 0.51 | 0.52 | 0.35 | 0.78 | 0.45 | 0.58 | 0.47 | 0.72 | 0.61 |
| rs4134822 | 111 | Li Y-L | 18 | 1.11 | 0.79 | 1.55 | 0.10 | 1.00 | 0.06 | 16.02 | 0.01 | 1.12 | 0.79 | 1.58 | 0.17 |
| rs1155563 | 112 | Pena-Chilet M | 21 | 1.01 | 0.81 | 1.26 | 0.25 | 1.15 | 0.71 | 1.88 | 0.20 | 0.97 | 0.73 | 1.29 | 0.28 |
| rs11574143 | 113 | Pena-Chilet M | 21 | 1.02 | 0.72 | 1.44 | 0.10 | 1.61 | 0.10 | 25.80 | 0.01 | 1.01 | 0.70 | 1.47 | 0.16 |
| rs12512631 | 114 | Pena-Chilet M | 21 | 0.78 | 0.63 | 0.96 | 0.31 | 1.07 | 0.69 | 1.64 | 0.27 | 0.61 | 0.46 | 0.82 | 0.34 |
| rs1352844 | 115 | Pena-Chilet M | 21 | 0.98 | 0.70 | 1.38 | 0.11 | 0.70 | 0.19 | 2.67 | 0.04 | 1.01 | 0.70 | 1.46 | 0.16 |
| rs1352845 | 116 | Pena-Chilet M | 21 | 0.92 | 0.69 | 1.24 | 0.15 | 0.92 | 0.27 | 3.08 | 0.04 | 0.91 | 0.65 | 1.26 | 0.22 |
| rs188812 | 117 | Pena-Chilet M | 21 | 0.81 | 0.57 | 1.16 | 0.11 | 3.42 | 0.31 | 37.91 | 0.00 | 0.76 | 0.52 | 1.12 | 0.18 |
| rs222016 | 118 | Pena-Chilet M | 21 | 0.78 | 0.57 | 1.06 | 0.15 | 1.05 | 0.34 | 3.25 | 0.04 | 0.73 | 0.52 | 1.03 | 0.23 |
| rs222049 | 119 | Pena-Chilet M | 21 | 1.20 | 0.83 | 1.74 | 0.08 | 0.89 | 0.08 | 9.84 | 0.01 | 1.23 | 0.84 | 1.82 | 0.13 |
| rs3733359 | 120 | Pena-Chilet M | 21 | 0.90 | 0.51 | 1.60 | 0.04 | 1.92 | 0.12 | 30.74 | 0.00 | 0.90 | 0.50 | 1.61 | 0.07 |
| rs4237855 | 121 | Pena-Chilet M | 21 | 1.03 | 0.83 | 1.28 | 0.26 | 1.07 | 0.78 | 1.48 | 0.48 | 1.00 | 0.73 | 1.37 | 0.22 |
| rs4334089 | 122 | Pena-Chilet M | 21 | 1.17 | 0.93 | 1.46 | 0.22 | 1.22 | 0.71 | 2.07 | 0.16 | 1.21 | 0.90 | 1.62 | 0.23 |
| rs4588 | 123 | Pena-Chilet M | 21 | 1.00 | 0.80 | 1.24 | 0.26 | 1.21 | 0.74 | 1.97 | 0.19 | 0.94 | 0.71 | 1.24 | 0.29 |
| rs4760658 | 124 | Pena-Chilet M | 21 | 0.89 | 0.71 | 1.10 | 0.28 | 0.65 | 0.41 | 1.05 | 0.30 | 0.96 | 0.72 | 1.27 | 0.27 |
| rs7041 | 125 | Pena-Chilet M | 21 | 0.91 | 0.74 | 1.11 | 0.31 | 1.04 | 0.73 | 1.48 | 0.40 | 0.77 | 0.57 | 1.05 | 0.26 |
| rs705119 | 126 | Pena-Chilet M | 21 | 0.94 | 0.77 | 1.16 | 0.30 | 1.16 | 0.80 | 1.67 | 0.35 | 0.79 | 0.58 | 1.07 | 0.26 |
| rs7299460 | 127 | Pena-Chilet M | 21 | 1.10 | 0.89 | 1.37 | 0.25 | 1.17 | 0.73 | 1.88 | 0.21 | 1.12 | 0.84 | 1.50 | 0.25 |
| rs739837 | 128 | Pena-Chilet M | 21 | 0.97 | 0.79 | 1.19 | 0.30 | 0.93 | 0.66 | 1.31 | 0.45 | 0.99 | 0.71 | 1.38 | 0.20 |
| rs11547464 | 129 | Helsing P | 22 | 1.86 | 0.46 | 7.46 | 0.00 | 0.93 | 0.06 | 14.86 | 0.01 | 1.86 | 0.46 | 7.51 | 0.01 |
| rs1408799 | 130 | Helsing P | 22 | 1.06 | 0.84 | 1.34 | 0.22 | 1.33 | 0.68 | 2.63 | 0.10 | 1.04 | 0.78 | 1.38 | 0.27 |
| rs3212363 | 131 | Helsing P | 22 | 0.96 | 0.78 | 1.19 | 0.28 | 1.21 | 0.75 | 1.95 | 0.21 | 0.87 | 0.66 | 1.15 | 0.31 |
| rs1015363 | 132 | Maccioni L | 23 | 0.95 | 0.83 | 1.09 | 0.66 | 1.06 | 0.82 | 1.37 | 0.75 | 0.87 | 0.71 | 1.06 | 0.57 |
| rs1018503 | 133 | Maccioni L | 23 | 1.04 | 0.91 | 1.19 | 0.65 | 1.16 | 0.92 | 1.47 | 0.88 | 0.98 | 0.79 | 1.21 | 0.49 |
| rs2064348 | 134 | Maccioni L | 23 | 1.05 | 0.86 | 1.30 | 0.27 | 1.00 | 0.42 | 2.38 | 0.07 | 1.07 | 0.85 | 1.34 | 0.41 |
| rs2268084 | 135 | Maccioni L | 23 | 0.88 | 0.77 | 1.01 | 0.70 | 0.83 | 0.65 | 1.06 | 0.97 | 0.85 | 0.69 | 1.05 | 0.57 |
| rs2268089 | 136 | Maccioni L | 23 | 0.85 | 0.73 | 0.98 | 0.59 | 0.87 | 0.61 | 1.24 | 0.44 | 0.79 | 0.65 | 0.96 | 0.67 |
| rs2378026 | 137 | Maccioni L | 23 | 1.03 | 0.87 | 1.23 | 0.42 | 1.09 | 0.67 | 1.76 | 0.21 | 1.03 | 0.85 | 1.26 | 0.54 |
| rs3043392 | 138 | Maccioni L | 23 | 1.10 | 0.96 | 1.27 | 0.59 | 1.18 | 0.89 | 1.57 | 0.58 | 1.11 | 0.91 | 1.35 | 0.54 |
| rs4911145 | 139 | Maccioni L | 23 | 0.87 | 0.75 | 1.00 | 0.65 | 0.83 | 0.61 | 1.12 | 0.63 | 0.83 | 0.68 | 1.00 | 0.64 |
| rs4911161 | 140 | Maccioni L | 23 | 1.02 | 0.89 | 1.16 | 0.65 | 1.15 | 0.89 | 1.48 | 0.75 | 0.94 | 0.77 | 1.16 | 0.55 |
| rs4911442 | 141 | Maccioni L | 23 | 0.60 | 0.48 | 0.76 | 0.29 | 0.63 | 0.28 | 1.41 | 0.10 | 0.57 | 0.44 | 0.74 | 0.45 |
| rs6087542 | 142 | Maccioni L | 23 | 1.07 | 0.92 | 1.24 | 0.55 | 1.04 | 0.76 | 1.43 | 0.51 | 1.10 | 0.91 | 1.33 | 0.57 |
| rs6087626 | 143 | Maccioni L | 23 | 1.30 | 1.14 | 1.49 | 0.59 | 1.21 | 0.93 | 1.56 | 0.70 | 1.52 | 1.26 | 1.84 | 0.50 |
| rs6088618 | 144 | Maccioni L | 23 | 1.09 | 0.95 | 1.25 | 0.63 | 1.10 | 0.86 | 1.40 | 0.84 | 1.14 | 0.92 | 1.40 | 0.48 |
| rs6088619 | 145 | Maccioni L | 23 | 0.98 | 0.79 | 1.21 | 0.28 | 0.75 | 0.28 | 2.00 | 0.06 | 0.99 | 0.79 | 1.24 | 0.42 |
| rs6120513 | 146 | Maccioni L | 23 | 0.83 | 0.72 | 0.95 | 0.65 | 0.73 | 0.55 | 0.98 | 0.70 | 0.81 | 0.66 | 0.98 | 0.63 |
| rs6142044 | 147 | Maccioni L | 23 | 1.05 | 0.92 | 1.21 | 0.61 | 1.00 | 0.76 | 1.32 | 0.68 | 1.10 | 0.91 | 1.34 | 0.55 |
| rs6142047 | 148 | Maccioni L | 23 | 0.91 | 0.80 | 1.05 | 0.67 | 0.90 | 0.70 | 1.17 | 0.84 | 0.88 | 0.72 | 1.08 | 0.60 |
| rs761237 | 149 | Maccioni L | 23 | 1.07 | 0.86 | 1.33 | 0.26 | 1.12 | 0.46 | 2.76 | 0.06 | 1.08 | 0.85 | 1.36 | 0.39 |
| rs910871 | 150 | Maccioni L | 23 | 0.76 | 0.63 | 0.93 | 0.35 | 0.70 | 0.35 | 1.40 | 0.13 | 0.74 | 0.60 | 0.93 | 0.51 |
| rs1799782 | 151 | Santonocito C | 24 | 0.92 | 0.44 | 1.89 | 0.02 | 1.70 | 0.11 | 27.55 | 0.00 | 0.82 | 0.38 | 1.78 | 0.04 |
| rs3136025 | 152 | Santonocito C | 24 | 1.69 | 0.11 | 27.12 | 0.00 | 1.69 | 0.10 | 27.27 | 0.00 | 1.69 | 0.10 | 27.27 | 0.00 |
| rs3219466 | 153 | Santonocito C | 24 | 0.89 | 0.39 | 2.03 | 0.02 | 1.69 | 0.10 | 27.27 | 0.00 | 0.88 | 0.38 | 2.06 | 0.03 |
| rs3212929 | 154 | Gao R | 25 | 10.98 | 1.34 | 89.96 | 0.00 | 1.52 | 0.09 | 24.53 | 0.01 | 11.38 | 1.38 | 94.02 | 0.00 |
| rs3212948 | 155 | Gao R | 25 | 1.94 | 1.33 | 2.84 | 0.06 | 2.55 | 1.19 | 5.46 | 0.05 | 2.29 | 1.32 | 3.97 | 0.05 |
| rs3212950 | 156 | Gao R | 25 | 1.79 | 1.22 | 2.62 | 0.06 | 2.36 | 1.09 | 5.07 | 0.06 | 2.05 | 1.19 | 3.53 | 0.05 |
| rs1143623 | 157 | Rizzato C | 26 | 1.06 | 0.87 | 1.28 | 0.32 | 1.21 | 0.77 | 1.92 | 0.22 | 1.03 | 0.81 | 1.32 | 0.36 |
| rs1143627 | 158 | Rizzato C | 26 | 1.15 | 0.95 | 1.39 | 0.31 | 1.21 | 0.81 | 1.81 | 0.29 | 1.18 | 0.92 | 1.53 | 0.31 |
| rs16944 | 159 | Rizzato C | 26 | 1.15 | 0.95 | 1.38 | 0.33 | 1.25 | 0.85 | 1.84 | 0.31 | 1.17 | 0.91 | 1.50 | 0.33 |
| rs17561 | 160 | Rizzato C | 26 | 1.08 | 0.89 | 1.30 | 0.33 | 1.12 | 0.73 | 1.72 | 0.26 | 1.10 | 0.86 | 1.40 | 0.34 |
| rs1800629 | 161 | Rizzato C | 26 | 0.75 | 0.58 | 0.95 | 0.23 | 0.38 | 0.17 | 0.88 | 0.13 | 0.78 | 0.59 | 1.02 | 0.32 |
| rs1800797 | 162 | Rizzato C | 26 | 1.13 | 0.95 | 1.35 | 0.37 | 0.94 | 0.68 | 1.30 | 0.50 | 1.35 | 1.04 | 1.74 | 0.29 |
| rs2069762 | 163 | Rizzato C | 26 | 0.99 | 0.82 | 1.19 | 0.36 | 1.03 | 0.70 | 1.51 | 0.34 | 0.97 | 0.76 | 1.25 | 0.36 |
| rs2070874 | 164 | Rizzato C | 26 | 0.95 | 0.75 | 1.20 | 0.23 | 0.73 | 0.34 | 1.55 | 0.11 | 0.97 | 0.74 | 1.27 | 0.32 |
| rs2243250 | 165 | Rizzato C | 26 | 0.98 | 0.78 | 1.24 | 0.23 | 0.71 | 0.34 | 1.45 | 0.12 | 1.03 | 0.79 | 1.34 | 0.31 |
| rs2430561 | 166 | Rizzato C | 26 | 1.05 | 0.88 | 1.26 | 0.37 | 1.20 | 0.89 | 1.62 | 0.52 | 0.97 | 0.74 | 1.27 | 0.31 |
| rs3024505 | 167 | Rizzato C | 26 | 1.27 | 1.00 | 1.62 | 0.19 | 1.50 | 0.67 | 3.37 | 0.07 | 1.30 | 0.99 | 1.71 | 0.26 |
| rs3212227 | 168 | Rizzato C | 26 | 0.95 | 0.77 | 1.19 | 0.27 | 1.06 | 0.57 | 1.95 | 0.13 | 0.93 | 0.72 | 1.20 | 0.35 |
| rs4073 | 169 | Rizzato C | 26 | 1.14 | 0.96 | 1.36 | 0.37 | 1.14 | 0.85 | 1.54 | 0.54 | 1.22 | 0.93 | 1.59 | 0.28 |
| rs419598 | 170 | Rizzato C | 26 | 1.02 | 0.83 | 1.27 | 0.26 | 0.60 | 0.34 | 1.07 | 0.20 | 1.16 | 0.89 | 1.50 | 0.30 |
| rs4848306 | 171 | Rizzato C | 26 | 0.90 | 0.75 | 1.07 | 0.42 | 0.88 | 0.65 | 1.20 | 0.57 | 0.85 | 0.65 | 1.11 | 0.33 |
| rs10202360 | 172 | Rizzato C | 27 | 1.06 | 0.85 | 1.32 | 0.25 | 1.22 | 0.60 | 2.51 | 0.09 | 1.05 | 0.82 | 1.36 | 0.34 |
| rs1042571 | 173 | Rizzato C | 27 | 0.93 | 0.74 | 1.18 | 0.23 | 0.56 | 0.23 | 1.35 | 0.09 | 0.97 | 0.74 | 1.26 | 0.32 |
| rs1050540 | 174 | Rizzato C | 27 | 0.95 | 0.79 | 1.13 | 0.39 | 1.01 | 0.70 | 1.45 | 0.39 | 0.90 | 0.70 | 1.15 | 0.38 |
| rs1050541 | 175 | Rizzato C | 27 | 1.01 | 0.85 | 1.20 | 0.41 | 1.08 | 0.82 | 1.43 | 0.62 | 0.95 | 0.72 | 1.25 | 0.30 |
| rs12602273 | 176 | Rizzato C | 27 | 1.11 | 0.80 | 1.54 | 0.11 | 0.66 | 0.11 | 3.98 | 0.02 | 1.15 | 0.81 | 1.62 | 0.17 |
| rs12951053 | 177 | Rizzato C | 27 | 1.10 | 0.81 | 1.50 | 0.12 | 2.35 | 0.60 | 9.13 | 0.02 | 1.06 | 0.76 | 1.47 | 0.20 |
| rs13002622 | 178 | Rizzato C | 27 | 1.08 | 0.90 | 1.30 | 0.34 | 1.06 | 0.69 | 1.63 | 0.28 | 1.12 | 0.88 | 1.43 | 0.35 |
| rs1641510 | 179 | Rizzato C | 27 | 1.07 | 0.90 | 1.27 | 0.39 | 1.05 | 0.77 | 1.42 | 0.54 | 1.12 | 0.87 | 1.45 | 0.32 |
| rs1642763 | 180 | Rizzato C | 27 | 0.89 | 0.71 | 1.10 | 0.28 | 1.05 | 0.59 | 1.87 | 0.15 | 0.84 | 0.65 | 1.08 | 0.37 |
| rs1866146 | 181 | Rizzato C | 27 | 0.95 | 0.80 | 1.13 | 0.40 | 0.87 | 0.60 | 1.25 | 0.42 | 0.96 | 0.75 | 1.24 | 0.35 |
| rs2287499 | 182 | Rizzato C | 27 | 1.19 | 0.92 | 1.55 | 0.16 | 2.41 | 0.84 | 6.89 | 0.03 | 1.15 | 0.86 | 1.54 | 0.25 |
| rs28932474 | 183 | Rizzato C | 27 | 1.12 | 0.75 | 1.66 | 0.07 | 0.99 | 0.06 | 15.82 | 0.01 | 1.12 | 0.75 | 1.69 | 0.12 |
| rs2909430 | 184 | Rizzato C | 27 | 0.99 | 0.77 | 1.29 | 0.18 | 0.66 | 0.29 | 1.49 | 0.10 | 1.05 | 0.78 | 1.40 | 0.26 |
| rs7566506 | 185 | Rizzato C | 27 | 1.13 | 0.82 | 1.57 | 0.11 | 1.65 | 0.39 | 6.93 | 0.02 | 1.11 | 0.79 | 1.58 | 0.17 |
| rs8073498 | 186 | Rizzato C | 27 | 0.89 | 0.75 | 1.06 | 0.42 | 0.80 | 0.57 | 1.12 | 0.51 | 0.89 | 0.69 | 1.15 | 0.37 |
| rs9895829 | 187 | Rizzato C | 27 | 1.00 | 0.77 | 1.29 | 0.18 | 0.60 | 0.25 | 1.47 | 0.09 | 1.06 | 0.79 | 1.41 | 0.26 |
| rs26722 | 188 | Guedj M | 28 | 0.14 | 0.06 | 0.31 | 0.07 | 1.11 | 0.19 | 6.65 | 0.02 | 0.08 | 0.03 | 0.23 | 0.12 |
| rs4778138 | 189 | Guedj M | 28 | 1.25 | 1.08 | 1.45 | 0.50 | 1.20 | 0.79 | 1.82 | 0.28 | 1.32 | 1.11 | 1.57 | 0.64 |
| rs4778241 | 190 | Guedj M | 28 | 1.06 | 0.92 | 1.22 | 0.59 | 1.03 | 0.72 | 1.48 | 0.39 | 1.08 | 0.91 | 1.29 | 0.69 |
| rs7495174 | 191 | Guedj M | 28 | 1.21 | 1.01 | 1.46 | 0.33 | 1.17 | 0.62 | 2.19 | 0.12 | 1.24 | 1.01 | 1.53 | 0.48 |
| rs1042602 | 192 | Fernandez LP | 29 | 1.03 | 0.76 | 1.39 | 0.13 | 1.04 | 0.62 | 1.74 | 0.19 | 1.04 | 0.65 | 1.67 | 0.10 |
| rs1052165 | 193 | Fernandez LP | 29 | 1.18 | 0.83 | 1.68 | 0.09 | 1.32 | 0.53 | 3.27 | 0.06 | 1.22 | 0.79 | 1.87 | 0.11 |
| rs1052206 | 194 | Fernandez LP | 29 | 0.87 | 0.58 | 1.29 | 0.08 | 0.80 | 0.22 | 2.88 | 0.03 | 0.85 | 0.54 | 1.35 | 0.11 |
| rs11791497 | 195 | Fernandez LP | 29 | 0.83 | 0.43 | 1.57 | 0.03 | 0.54 | 0.03 | 8.65 | 0.01 | 0.84 | 0.42 | 1.66 | 0.05 |
| rs12379024 | 196 | Fernandez LP | 29 | 0.86 | 0.63 | 1.16 | 0.15 | 0.79 | 0.47 | 1.34 | 0.21 | 0.81 | 0.51 | 1.29 | 0.11 |
| rs12592307 | 197 | Fernandez LP | 29 | 0.99 | 0.69 | 1.43 | 0.09 | 1.07 | 0.39 | 2.93 | 0.05 | 1.01 | 0.65 | 1.56 | 0.12 |
| rs1800404 | 198 | Fernandez LP | 29 | 1.06 | 0.76 | 1.47 | 0.11 | 1.13 | 0.52 | 2.48 | 0.08 | 1.05 | 0.69 | 1.61 | 0.12 |
| rs1800411 | 199 | Fernandez LP | 29 | 0.92 | 0.68 | 1.25 | 0.14 | 0.88 | 0.50 | 1.57 | 0.16 | 0.92 | 0.59 | 1.43 | 0.12 |
| rs2000553 | 200 | Fernandez LP | 29 | 0.92 | 0.67 | 1.25 | 0.13 | 0.88 | 0.47 | 1.64 | 0.14 | 0.89 | 0.58 | 1.38 | 0.13 |
| rs2069391 | 201 | Fernandez LP | 29 | 1.66 | 0.87 | 3.16 | 0.02 | 1.07 | 0.10 | 11.91 | 0.01 | 1.81 | 0.91 | 3.59 | 0.04 |
| rs2733836 | 202 | Fernandez LP | 29 | 0.89 | 0.66 | 1.21 | 0.14 | 0.86 | 0.52 | 1.43 | 0.21 | 0.87 | 0.54 | 1.40 | 0.10 |
| rs35388 | 203 | Fernandez LP | 29 | 2.98 | 2.18 | 4.06 | 0.07 | 9.27 | 5.19 | 16.57 | 0.06 | 1.54 | 0.97 | 2.45 | 0.08 |
| rs35401 | 204 | Fernandez LP | 29 | 0.84 | 0.61 | 1.15 | 0.13 | 1.12 | 0.59 | 2.12 | 0.12 | 0.69 | 0.45 | 1.06 | 0.14 |
| rs35414 | 205 | Fernandez LP | 29 | 1.40 | 1.03 | 1.90 | 0.11 | 1.43 | 0.78 | 2.62 | 0.12 | 1.79 | 1.12 | 2.84 | 0.07 |
| rs3793976 | 206 | Fernandez LP | 29 | 0.92 | 0.58 | 1.48 | 0.06 | 0.80 | 0.13 | 4.85 | 0.02 | 0.89 | 0.53 | 1.48 | 0.09 |
| rs42868 | 207 | Fernandez LP | 29 | 1.17 | 0.77 | 1.78 | 0.06 | 1.34 | 0.26 | 7.02 | 0.02 | 1.19 | 0.74 | 1.91 | 0.09 |
| rs621313 | 208 | Fernandez LP | 29 | 0.85 | 0.63 | 1.16 | 0.14 | 0.80 | 0.46 | 1.40 | 0.18 | 0.81 | 0.52 | 1.28 | 0.12 |
| rs768617 | 209 | Fernandez LP | 29 | 11.05 | 6.63 | 18.41 | 0.02 | 33.33 | 4.55 | 244.31 | 0.01 | 14.51 | 8.23 | 25.58 | 0.02 |
| rs819164 | 210 | Fernandez LP | 29 | 1.57 | 0.93 | 2.65 | 0.04 | 2.15 | 0.24 | 19.43 | 0.01 | 1.64 | 0.93 | 2.87 | 0.06 |
| rs1052555 | 211 | Povey JE | 30 | 1.07 | 0.88 | 1.31 | 0.30 | 1.14 | 0.74 | 1.75 | 0.26 | 1.08 | 0.84 | 1.39 | 0.33 |
| rs3136820 | 212 | Li C | 32 | 1.15 | 0.98 | 1.35 | 0.45 | 1.08 | 0.83 | 1.41 | 0.71 | 1.30 | 1.01 | 1.67 | 0.31 |
| rs947894 | 213 | Oliveira C | 33 | 1.23 | 0.88 | 1.72 | 0.10 | 1.60 | 0.81 | 3.15 | 0.09 | 1.19 | 0.74 | 1.90 | 0.09 |
| rs2279115 | 214 | Oliveira C | 35 | 0.68 | 0.52 | 0.90 | 0.19 | 0.60 | 0.38 | 0.94 | 0.33 | 0.64 | 0.41 | 0.99 | 0.14 |
| rs4645878 | 215 | Oliveira C | 35 | 3.57 | 2.47 | 5.15 | 0.06 | 1.38 | 0.26 | 7.15 | 0.02 | 5.22 | 3.46 | 7.89 | 0.06 |
| rs2066827 | 216 | Francisco G | 36 | 1.69 | 1.23 | 2.32 | 0.09 | 2.40 | 1.10 | 5.26 | 0.06 | 1.89 | 1.24 | 2.88 | 0.09 |
| rs697221 | 217 | Francisco G | 36 | 0.95 | 0.68 | 1.34 | 0.11 | 1.06 | 0.38 | 2.99 | 0.05 | 0.92 | 0.62 | 1.38 | 0.14 |
| rs1800890 | 218 | Schoof N | 37 | 1.28 | 0.94 | 1.75 | 0.11 | 1.12 | 0.62 | 2.03 | 0.14 | 1.57 | 1.00 | 2.48 | 0.09 |
| rs6676671 | 219 | Schoof N | 37 | 1.22 | 0.89 | 1.67 | 0.11 | 1.03 | 0.56 | 1.88 | 0.14 | 1.47 | 0.93 | 2.31 | 0.09 |
| rs104894094 | 220 | Tovar-Parra JD | 39 | 0.25 | 0.02 | 2.81 | 0.00 | 0.51 | 0.03 | 8.29 | 0.01 | 0.25 | 0.02 | 2.80 | 0.01 |
| rs104894095 | 221 | Tovar-Parra JD | 39 | 0.51 | 0.03 | 8.24 | 0.00 | 0.51 | 0.03 | 8.29 | 0.01 | 0.51 | 0.03 | 8.29 | 0.00 |
| rs104894097 | 222 | Tovar-Parra JD | 39 | 0.68 | 0.15 | 3.07 | 0.01 | 0.51 | 0.03 | 8.29 | 0.01 | 0.68 | 0.15 | 3.09 | 0.01 |
| rs104894098 | 223 | Tovar-Parra JD | 39 | 0.51 | 0.07 | 3.65 | 0.00 | 0.51 | 0.03 | 8.29 | 0.01 | 0.51 | 0.07 | 3.66 | 0.01 |
| rs1801131 | 224 | Lesiak A | 46 | 0.73 | 0.51 | 1.06 | 0.11 | 0.26 | 0.07 | 0.94 | 0.07 | 0.75 | 0.47 | 1.20 | 0.12 |
| rs1801133 | 225 | Lesiak A | 46 | 0.48 | 0.31 | 0.74 | 0.10 | 0.77 | 0.34 | 1.75 | 0.09 | 0.37 | 0.22 | 0.62 | 0.13 |
| rs1799750 | 226 | Wang L-E | 47 | 1.00 | 0.87 | 1.14 | 0.68 | 1.03 | 0.82 | 1.29 | 0.98 | 0.97 | 0.78 | 1.19 | 0.50 |
| rs473509 | 227 | Wang L-E | 47 | 1.10 | 0.96 | 1.26 | 0.64 | 1.18 | 0.92 | 1.51 | 0.77 | 1.11 | 0.91 | 1.35 | 0.52 |
| rs1052559 | 228 | Vogel U | 51 | 1.15 | 0.92 | 1.44 | 0.22 | 1.19 | 0.76 | 1.87 | 0.23 | 1.21 | 0.88 | 1.67 | 0.20 |
| rs1970764 | 229 | Vogel U | 51 | 0.95 | 0.71 | 1.27 | 0.15 | 0.70 | 0.28 | 1.77 | 0.07 | 0.98 | 0.70 | 1.37 | 0.20 |
| rs3177700 | 230 | Vogel U | 51 | 0.85 | 0.68 | 1.07 | 0.25 | 0.91 | 0.58 | 1.44 | 0.26 | 0.77 | 0.55 | 1.06 | 0.24 |
| rs6966 | 231 | Vogel U | 51 | 1.02 | 0.73 | 1.44 | 0.10 | 0.42 | 0.11 | 1.64 | 0.05 | 1.11 | 0.76 | 1.61 | 0.15 |
| rs967591 | 232 | Vogel U | 51 | 0.91 | 0.68 | 1.21 | 0.15 | 0.89 | 0.39 | 2.05 | 0.08 | 0.89 | 0.64 | 1.25 | 0.20 |
| rs486907 | 233 | Sangalli A | 52 | 1.19 | 0.95 | 1.50 | 0.21 | 1.29 | 0.82 | 2.02 | 0.22 | 1.24 | 0.90 | 1.72 | 0.19 |
| rs1800401 | 234 | Jannot A-S | 58 | 2.82 | 1.27 | 6.28 | 0.01 | 2.19 | 0.20 | 24.56 | 0.01 | 2.72 | 1.18 | 6.28 | 0.02 |
| rs1800407 | 235 | Jannot A-S | 58 | 1.02 | 0.52 | 2.00 | 0.03 | 3.32 | 0.34 | 32.46 | 0.01 | 0.83 | 0.40 | 1.72 | 0.05 |
| rs10069690 | 236 | Llorca-Cardenosa MJ | 13 | 1.08 | 0.90 | 1.29 | 0.35 | 0.93 | 0.53 | 1.65 | 0.16 | 1.11 | 0.90 | 1.37 | 0.47 |
| rs2242652 | 237 | Llorca-Cardenosa MJ | 13 | 1.07 | 0.86 | 1.33 | 0.25 | 0.97 | 0.56 | 1.67 | 0.17 | 1.11 | 0.86 | 1.45 | 0.30 |
| rs2736100 | 238 | Llorca-Cardenosa MJ | 13 | 0.91 | 0.76 | 1.09 | 0.39 | 0.89 | 0.66 | 1.20 | 0.61 | 0.88 | 0.66 | 1.17 | 0.29 |
| rs2736118 | 239 | Llorca-Cardenosa MJ | 13 | 1.06 | 0.85 | 1.31 | 0.25 | 0.97 | 0.56 | 1.69 | 0.17 | 1.09 | 0.84 | 1.42 | 0.30 |
| rs2853672 | 240 | Llorca-Cardenosa MJ | 13 | 1.01 | 0.88 | 1.16 | 0.62 | 1.10 | 0.86 | 1.41 | 0.83 | 0.95 | 0.77 | 1.18 | 0.49 |
| rs2853676 | 241 | Llorca-Cardenosa MJ | 13 | 1.02 | 0.84 | 1.24 | 0.31 | 1.09 | 0.68 | 1.73 | 0.23 | 1.01 | 0.78 | 1.30 | 0.33 |
| rs2853677 | 242 | Llorca-Cardenosa MJ | 13 | 1.13 | 0.99 | 1.30 | 0.60 | 1.28 | 1.00 | 1.63 | 0.78 | 1.11 | 0.89 | 1.37 | 0.46 |
| rs2853690 | 243 | Llorca-Cardenosa MJ | 13 | 1.08 | 0.90 | 1.29 | 0.35 | 0.93 | 0.53 | 1.65 | 0.16 | 1.11 | 0.90 | 1.37 | 0.47 |
| rs2981096 | 244 | Llorca-Cardenosa MJ | 13 | 0.91 | 0.54 | 1.53 | 0.05 | 0.59 | 0.04 | 9.48 | 0.01 | 0.88 | 0.52 | 1.50 | 0.08 |
| rs401681 | 245 | Llorca-Cardenosa MJ | 13 | 1.23 | 1.07 | 1.41 | 0.59 | 1.31 | 1.03 | 1.66 | 0.82 | 1.34 | 1.07 | 1.67 | 0.39 |
| rs4246742 | 246 | Llorca-Cardenosa MJ | 13 | 1.08 | 0.81 | 1.44 | 0.15 | 1.29 | 0.49 | 3.42 | 0.05 | 1.07 | 0.78 | 1.46 | 0.22 |
| rs4975605 | 247 | Llorca-Cardenosa MJ | 13 | 1.11 | 0.97 | 1.27 | 0.60 | 1.20 | 0.96 | 1.50 | 0.94 | 1.09 | 0.88 | 1.36 | 0.43 |
| rs1049255 | 248 | Yuan T-A | 16 | 0.63 | 0.46 | 0.88 | 0.15 | 0.51 | 0.28 | 0.92 | 0.22 | 0.59 | 0.37 | 0.95 | 0.12 |
| rs10951982 | 249 | Yuan T-A | 16 | 0.35 | 0.24 | 0.50 | 0.16 | 0.52 | 0.18 | 1.51 | 0.07 | 0.11 | 0.06 | 0.20 | 0.20 |
| rs2536512 | 250 | Yuan T-A | 16 | 1.58 | 1.10 | 2.26 | 0.07 | 11.19 | 2.49 | 50.24 | 0.01 | 1.45 | 0.89 | 2.36 | 0.08 |
| rs4673 | 251 | Yuan T-A | 16 | 0.76 | 0.55 | 1.07 | 0.13 | 1.49 | 0.57 | 3.89 | 0.05 | 0.55 | 0.34 | 0.86 | 0.14 |
| rs8031 | 252 | Yuan T-A | 16 | 1.58 | 1.14 | 2.20 | 0.09 | 6.77 | 2.87 | 15.96 | 0.03 | 1.20 | 0.71 | 2.04 | 0.07 |
| rs1625895 | 253 | Mukhammadiyeva GF | 17 | 0.48 | 0.20 | 1.13 | 0.02 | 0.24 | 0.01 | 3.98 | 0.01 | 0.50 | 0.19 | 1.33 | 0.03 |
| rs17878362 | 254 | Mukhammadiyeva GF | 17 | 0.57 | 0.25 | 1.28 | 0.02 | 0.24 | 0.01 | 3.98 | 0.01 | 0.59 | 0.23 | 1.51 | 0.03 |
| rs10741657 | 255 | Pena-Chilet M | 20 | 0.99 | 0.80 | 1.22 | 0.27 | 1.15 | 0.77 | 1.72 | 0.29 | 0.90 | 0.67 | 1.22 | 0.26 |
| rs12203592 | 256 | Pena-Chilet M | 20 | 1.04 | 0.79 | 1.35 | 0.17 | 0.16 | 0.04 | 0.69 | 0.10 | 1.21 | 0.90 | 1.63 | 0.22 |
| rs12785878 | 257 | Pena-Chilet M | 20 | 1.19 | 0.97 | 1.47 | 0.25 | 1.13 | 0.74 | 1.73 | 0.27 | 1.31 | 0.98 | 1.75 | 0.24 |
| rs13016963 | 258 | Pena-Chilet M | 20 | 1.07 | 0.87 | 1.30 | 0.29 | 1.15 | 0.79 | 1.68 | 0.33 | 1.05 | 0.78 | 1.41 | 0.25 |
| rs1485993 | 259 | Pena-Chilet M | 20 | 1.04 | 0.85 | 1.28 | 0.29 | 1.05 | 0.73 | 1.52 | 0.37 | 1.06 | 0.78 | 1.45 | 0.22 |
| rs1801516 | 260 | Pena-Chilet M | 20 | 1.23 | 0.91 | 1.65 | 0.12 | 1.79 | 0.73 | 4.38 | 0.05 | 1.19 | 0.85 | 1.67 | 0.18 |
| rs3219090 | 261 | Pena-Chilet M | 20 | 1.29 | 1.04 | 1.60 | 0.23 | 1.52 | 0.95 | 2.42 | 0.19 | 1.31 | 0.99 | 1.75 | 0.24 |
| rs45430 | 262 | Pena-Chilet M | 20 | 0.95 | 0.77 | 1.17 | 0.30 | 0.87 | 0.59 | 1.30 | 0.36 | 0.97 | 0.73 | 1.30 | 0.26 |
| rs7944926 | 263 | Pena-Chilet M | 20 | 1.10 | 0.89 | 1.36 | 0.26 | 1.18 | 0.77 | 1.80 | 0.26 | 1.11 | 0.83 | 1.48 | 0.25 |
| rs1800795 | 264 | Slawinska M | 40 | 1.37 | 1.04 | 1.81 | 0.14 | 1.61 | 0.99 | 2.62 | 0.18 | 1.36 | 0.93 | 1.98 | 0.13 |
| rs2293152 | 265 | Slawinska M | 40 | 1.35 | 1.03 | 1.77 | 0.14 | 3.25 | 1.89 | 5.59 | 0.11 | 0.91 | 0.62 | 1.34 | 0.15 |
| rs4796793 | 266 | Slawinska M | 40 | 1.58 | 1.20 | 2.10 | 0.12 | 2.69 | 1.54 | 4.72 | 0.11 | 1.39 | 0.95 | 2.02 | 0.13 |
| rs3200401 | 267 | Orlandi E | 41 | 0.96 | 0.74 | 1.25 | 0.18 | 1.29 | 0.68 | 2.45 | 0.11 | 0.89 | 0.65 | 1.22 | 0.23 |
| rs619586 | 268 | Orlandi E | 41 | 0.77 | 0.41 | 1.45 | 0.04 | 1.15 | 0.07 | 18.43 | 0.01 | 0.77 | 0.41 | 1.45 | 0.06 |
| rs1063045 | 269 | Meyer P | 50 | 1.06 | 0.89 | 1.25 | 0.41 | 0.98 | 0.68 | 1.42 | 0.38 | 1.11 | 0.89 | 1.39 | 0.42 |
| rs867185 | 270 | Meyer P | 50 | 1.15 | 0.97 | 1.35 | 0.42 | 1.14 | 0.82 | 1.57 | 0.46 | 1.22 | 0.97 | 1.53 | 0.39 |
| rs9995 | 271 | Meyer P | 50 | 0.99 | 0.84 | 1.18 | 0.42 | 1.00 | 0.68 | 1.46 | 0.35 | 0.99 | 0.79 | 1.23 | 0.44 |
| rs2297518 | 272 | Li C | 57 | 1.12 | 0.91 | 1.38 | 0.27 | 1.67 | 0.85 | 3.27 | 0.09 | 1.09 | 0.86 | 1.38 | 0.37 |
| rs2682826 | 273 | Li C | 57 | 1.05 | 0.88 | 1.26 | 0.38 | 1.07 | 0.70 | 1.64 | 0.28 | 1.07 | 0.85 | 1.34 | 0.42 |
| rs2779249 | 274 | Li C | 57 | 1.02 | 0.86 | 1.21 | 0.40 | 0.83 | 0.57 | 1.21 | 0.41 | 1.11 | 0.88 | 1.39 | 0.41 |
| Overall |  |  |  | 1.01 | 1.00 | 1.02 |  | 1.16 | 1.13 | 1.18 |  | 1.06 | 1.05 | 1.08 |  |

ID, the code of SNP; Code, the code of article; 95% CI, confidence interval; L, the low of 95% CI; H, the high of 95% CI; W (%), weight (%)
