# Supplementary material for: The relationship between single nucleotide polymorphisms and skin cancer susceptibility: A systematic review and network meta-analysis
Source: Front Oncol. 2023 Feb 15;13:1094309. doi: 10.3389/fonc.2023.1094309 (PMC9975575; doi:10.3389/fonc.2023.1094309)
Supplement: Supplementary file 2 [file Table_2.docx]

Table 2. The alleles comparisons of enrolled studies in the allele model

| Gene | SNP | Author, Year | Comparisons | Case | | Control | | OR | cOR |
| --- | --- | --- | --- | --- | --- | --- | --- | --- | --- |
|  |  |  |  | A | B | A | B |  |  |
| AFG3L1P | rs4785763 | Ozola A, 2019 | A vs. C | 298 | 212 | 269 | 141 | **0.74*** | - |
| BAX | rs4645878 | Oliveira C, 2014 | G vs. A | 361 | 39 | 527 | 203 | **3.57*** | - |
| CDK10 | rs258322 | Ozola A, 2019 | A vs. G | 450 | 60 | 379 | 27 | **0.53*** | - |
| CDKN1B | rs2066827 | Francisco G, 2013 | Val vs. Gly | 273 | 95 | 218 | 128 | **1.69*** | - |
| CDKN2A | rs3088440 | Maccioni L, 2013(1) | C vs. T | 1428 | 118 | 2148 | 118 | **0.66*** | 0.67 |
|  |  | Pjanova D,2007 |  | 225 | 25 | 378 | 28 | 0.67 |  |
|  | rs3731249 | Tovar-Parra JD, 2020 | G vs. A | 165 | 5 | 324 | 8 | 0.81 | 0.46 |
|  |  | Pjanova D,2007 |  | 242 | 8 | 403 | 3 | **0.23*** |  |
| CDKN2B | rs1011970 | Maccioni L, 2013(1) | G vs. T | 1288 | 252 | 1977 | 315 | **0.81*** | - |
| CLPTM1L | rs401681 | Llorca-Cardenosa MJ, 2014 | C vs. T | 787 | 657 | 945 | 967 | **1.23*** | - |
| CYBA | rs1049255 | Yuan T-A, 2018 | C vs. T | 171 | 159 | 175 | 103 | **0.63*** | - |
| EIF2S2 | rs6120513 | Maccioni L, 2013(2) | C vs. A | 891 | 541 | 1253 | 629 | **0.83*** | - |
| ERCC1 | rs11615 | Gao R, 2013 | A vs. G | 208 | 104 | 117 | 99 | **1.69*** | 1.12 |
|  |  | Povey JE, 2007 |  | 680 | 334 | 590 | 288 | 0.99 |  |
|  | rs3212929 | Gao R, 2013 | G vs. T | 287 | 1 | 183 | 7 | **10.98*** | - |
|  | rs3212948 | Gao R, 2013 | G vs. C | 202 | 86 | 104 | 86 | **1.94*** | - |
|  | rs3212950 | Gao R, 2013 | G vs. C | 201 | 87 | 107 | 83 | **1.79*** | - |
| ERCC2 | rs13181 | Applebaum KM, 2007 | A vs. C | 2182 | 1814 | 1075 | 955 | 1.07 | 0.96 |
|  |  | Figl A, 2010 |  | 1595 | 953 | 1553 | 819 | **0.88*** |  |
|  |  | Povey JE, 2007 |  | 684 | 330 | 684 | 330 | 1.00 |  |
|  |  | Li C, 2006(1) |  | 735 | 469 | 780 | 426 | 0.86 |  |
| ERCC2 | rs1799793 | Applebaum KM, 2007 | G vs. A | 2046 | 1724 | 1029 | 939 | 1.08 | 0.99 |
|  |  | Figl A, 2010 |  | 1626 | 922 | 1626 | 922 | 1.00 |  |
|  |  | Li Y-L, 2017 |  | 933 | 387 | 992 | 332 | **0.81*** |  |
|  |  | Li C, 2006(1) |  | 774 | 430 | 805 | 401 | 0.90 |  |
|  |  | Vogel U, 2005 |  | 421 | 215 | 419 | 223 | 1.04 |  |
| ERCC6 | rs2228527 | Li Y-L, 2017 | A vs. G | 930 | 390 | 1040 | 284 | **0.65*** | - |
|  | rs2228529 | Li Y-L, 2017 | A vs. G | 910 | 414 | 1031 | 293 | **0.62*** | - |
| GSTP1 | rs1695 | Hsu L-I, 2015 | W vs. M | 114 | 26 | 345 | 75 | 0.95 | **0.79*** |
|  |  | Ibarrola-Villava M, 2012 |  | 749 | 375 | 490 | 186 | **0.76*** |  |
| HERC2 | rs1129038 | Reis LB, 2020 | A vs. G | 147 | 93 | 132 | 138 | **1.65*** | - |
| IL10 | rs3024505 | Rizzato C, 2011(1) | C vs. T | 881 | 143 | 857 | 177 | **1.27*** | - |
| IL-6 | rs1800795 | Slawinska M, 2019 | G vs. C | 260 | 136 | 283 | 203 | **1.37*** | - |
| LOC107987026 | rs1335510 | Maccioni L, 2013(1) | T vs. G | 998 | 546 | 1380 | 914 | **1.21*** | - |
|  | rs2218220 | Maccioni L, 2013(1) | T vs. C | 713 | 835 | 1169 | 1135 | **0.83*** | - |
|  | rs4636294 | Maccioni L, 2013(1) | G vs. A | 713 | 833 | 1165 | 1141 | **0.84*** | - |
|  | rs751173 | Maccioni L, 2013(1) | T vs. C | 806 | 742 | 1289 | 1015 | **0.86*** | - |
| LURAP1L | rs768617 | Fernandez LP, 2008 | C vs. T | 244 | 18 | 270 | 220 | **11.05*** | - |
| MC1R | rs1805006 | Cordoba-Lanus E, 2014 | C vs. A | 960 | 4 | 1005 | 13 | **3.10*** | 0.64 |
|  |  | Helsing P, 2012 |  | 755 | 21 | 830 | 4 | **0.17*** |  |
|  | rs1805007 | Motorina AV, 2018 | C vs. T | 649 | 11 | 162 | 16 | **5.83*** | 0.87 |
|  |  | Cordoba-Lanus E, 2014 |  | 939 | 21 | 958 | 58 | **2.71*** |  |
|  |  | Ozola A, 2019 |  | 469 | 41 | 429 | 17 | **0.45*** |  |
|  |  | Helsing P, 2012 |  | 626 | 148 | 737 | 97 | **0.56*** |  |
|  | rs1805008 | Motorina AV, 2018 | C vs. T | 656 | 1 | 176 | 1 | 3.73 | 0.89 |
|  |  | Cordoba-Lanus E, 2014 |  | 939 | 7 | 995 | 23 | **3.10*** |  |
|  |  | Ozola A, 2019 |  | 444 | 66 | 400 | 40 | 0.67 |  |
|  |  | Helsing P, 2012 |  | 661 | 113 | 728 | 104 | 0.84 |  |
|  | rs885479 | Cordoba-Lanus E, 2014 | G vs. A | 951 | 9 | 975 | 43 | **4.66*** | **1.46*** |
|  |  | Helsing P, 2012 |  | 719 | 57 | 776 | 58 | 0.94 |  |
| MMP1 | rs1051121 | Debniak T, 2011 | Wild-type Allele vs. Mutant Allele | 558 | 6 | 521 | 15 | **2.68*** | - |
| MMP8 | rs11225395 | Debniak T, 2011 | A vs. G | 324 | 268 | 360 | 220 | **0.74*** | - |
| MTAP | rs10757257 | Maccioni L, 2013(1) | G vs. A | 1000 | 548 | 1381 | 921 | **1.22*** | - |
|  | rs10811629 | Maccioni L, 2013(1) | A vs. G | 988 | 560 | 1369 | 927 | **1.19*** | - |
|  | rs1341866 | Maccioni L, 2013(1) | T vs. C | 993 | 555 | 1372 | 930 | **1.21*** | - |
|  | rs7023329 | Maccioni L, 2013(1) | A vs. G | 837 | 711 | 1156 | 1150 | **1.17*** | - |
| near MTAP | rs935053 | Maccioni L, 2013(1) | A vs. G | 717 | 829 | 1187 | 1119 | **0.82*** | - |
| MTHFR | rs1801133 | Lesiak A, 2011 | C vs. T | 213 | 71 | 245 | 39 | **0.48*** | - |
| NCOA6 | rs4911442 | Maccioni L, 2013(2) | A vs. G | 1294 | 172 | 1817 | 145 | **0.60*** | - |
|  | rs6087626 | Maccioni L, 2013(2) | C vs. T | 1094 | 574 | 1165 | 797 | **1.30*** | - |
|  | rs910871 | Maccioni L, 2013(2) | A vs. C | 1253 | 217 | 1731 | 229 | **0.76*** | - |
| near ASIP | rs4911414 | Helsing P, 2012 | G vs. T | 506 | 266 | 582 | 252 | 0.82 | **0.85*** |
|  |  | Maccioni L, 2013(2) |  | 1026 | 438 | 1431 | 523 | **0.86*** |  |
| OCA2 | rs4778138 | Guedj M, 2008 | T vs. C | 1595 | 345 | 2145 | 581 | **1.25*** | - |
|  | rs7495174 | Guedj M, 2008 | T vs. C | 1776 | 200 | 2423 | 331 | **1.21*** | - |
|  | rs1800401 | Jannot A-S, 2005 | C vs. T | 217 | 9 | 188 | 22 | **2.82*** | - |
| P53 | rs1042522 | Thunell LK, 2014 | G vs. C | 376 | 138 | 1194 | 404 | 0.92 | 1.04 |
|  |  | Ozola A, 2019 |  | 361 | 149 | 286 | 150 | 1.27 |  |
|  |  | Rizzato C, 2011(2) |  | 770 | 266 | 772 | 270 | 1.01 |  |
|  |  | Povey JE, 2007 |  | 801 | 275 | 616 | 234 | 1.11 |  |
|  |  | Oliveira C, 2013 |  | 222 | 70 | 199 | 93 | **1.48*** |  |
|  |  | Nan H, 2009 |  | 1137 | 413 | 1273 | 393 | **0.85*** |  |
|  |  | Oliveira C, 2014 |  | 296 | 104 | 349 | 209 | **1.70*** |  |
|  |  | Francisco G, 2013 |  | 248 | 130 | 260 | 156 | 1.14 |  |
|  |  | Capasso M, 2010 |  | 282 | 206 | 342 | 240 | 0.96 |  |
|  |  | Almquist LM, 2011 |  | 2217 | 703 | 1166 | 368 | 1.00 |  |
| PARP1 | rs1136410 | Santonocito C, 2012 | Val vs. Ala | 259 | 75 | 177 | 21 | **0.41*** | 0.97 |
|  |  | Li C, 2006(2) |  | 1021 | 183 | 999 | 207 | 1.16 |  |
|  | rs3219090 | Pena-Chilet M, 2013(1) | Wild-type Allele vs. Mutant Allele | 694 | 264 | 431 | 211 | **1.29*** | - |
| PDCD1 | rs11568821 | Fathi F, 2019 | G vs. A | 390 | 30 | 568 | 72 | **1.65*** | - |
|  | rs41386349 | Gomez GVB, 2018 | C vs. T | 479 | 21 | 460 | 40 | **1.98*** | - |
| PTGS2 | rs20417 | Gomez-Lira M, 2014 | G vs. C | 545 | 139 | 363 | 123 | **1.33*** | 1.23 |
|  |  | Cocos R, 2012 |  | 276 | 72 | 250 | 70 | 1.07 |  |
| RAC1 | rs10951982 | Yuan T-A, 2018 | G vs. A | 173 | 155 | 194 | 60 | **0.35*** | - |
| RALY | rs2268089 | Maccioni L, 2013(2) | C vs. T | 1030 | 438 | 1439 | 517 | **0.84*** | - |
|  | rs4911145 | Maccioni L, 2013(2) | G vs. A | 930 | 534 | 1311 | 651 | **0.86*** | - |
| SLC45A2 | rs16891982 | Reis LB, 2020 | G vs. C | 222 | 18 | 218 | 52 | **2.94*** | **2.74*** |
|  |  | Guedj M, 2008 |  | 1855 | 75 | 2566 | 286 | **2.76*** |  |
|  |  | Fernandez LP, 2008 |  | 244 | 18 | 414 | 76 | **2.49*** |  |
|  | rs1426654 | Reis LB, 2020 | A vs. G | 236 | 4 | 245 | 25 | **6.02*** | - |
|  | rs26722 | Guedj M, 2008 | G vs. A | 1899 | 37 | 2611 | 7 | **0.14*** | - |
|  | rs35388 | Fernandez LP, 2008 | Wild-type Allele vs. Mutant Allele | 160 | 102 | 169 | 321 | **2.98*** | - |
|  | rs35414 | Fernandez LP, 2008 | Wild-type Allele vs. Mutant Allele | 160 | 102 | 259 | 231 | **1.40*** | - |
| SOD2 | rs8031 | Yuan T-A, 2018 | A vs. T | 201 | 125 | 134 | 132 | **1.58*** | - |
| SOD3 | rs2536512 | Yuan T-A, 2018 | G vs. A | 239 | 91 | 145 | 87 | **1.58*** | - |
| STAT3 | rs2293152 | Slawinska M, 2019 | G vs. C | 251 | 145 | 273 | 213 | **1.35*** | - |
|  | rs4796793 | Slawinska M, 2019 | C vs. G | 279 | 117 | 292 | 194 | **1.58*** | - |
| TNF | rs1800629 | Rizzato C, 2011(1) | G vs. A | 844 | 168 | 897 | 133 | **0.74*** | - |
| VDB | rs12512631 | Pena-Chilet M, 2013(2) | Wild-type Allele vs. Mutant Allele | 659 | 383 | 423 | 191 | **0.78*** | - |
| VDR | rs2228570 | Pena-Chilet M, 2013(2) | Wild-type Allele vs. Mutant Allele | 659 | 341 | 410 | 208 | 0.98 | **0.79*** |
|  |  | Lesiak A, 2011 |  | 125 | 159 | 208 | 76 | **0.29*** |  |
|  |  | Li C, 2008 |  | 1001 | 609 | 1084 | 598 | 0.91 |  |
|  |  | Aristizabal-Pachon A, 2022 |  | 132 | 108 | 170 | 70 | **0.50*** |  |
|  | rs1544410 | Morgado-Aguila C, 2020 | G vs. A | 101 | 61 | 84 | 62 | 1.22 | **1.19*** |
|  |  | Burns EM, 2017 |  | 104 | 90 | 128 | 72 | **0.65*** |  |
|  |  | Lesiak A, 2011 |  | 175 | 109 | 243 | 273 | **1.80*** |  |
|  |  | Li C, 2008 |  | 976 | 634 | 957 | 725 | **1.17*** |  |
|  | rs731236 | Pena-Chilet M, 2013(2) | T vs. C | 620 | 376 | 359 | 229 | 1.05 | **1.11*** |
|  |  | Burns EM, 2017 |  | 110 | 84 | 131 | 69 | 0.69 |  |
|  |  | Lesiak A, 2011 |  | 146 | 138 | 187 | 97 | **0.55*** |  |
|  |  | Li C, 2008 |  | 1015 | 595 | 960 | 722 | **1.28*** |  |
|  |  | Aristizabal-Pachon A, 2022 |  | 226 | 14 | 201 | 39 | **3.13*** |  |
| XPA | rs1800975 | Applebaum KM, 2007 | G vs. A | 2280 | 1726 | 1120 | 954 | **1.13*** | - |
| XRCC1 | rs25487 | Hsu L-I, 2015 | G vs. A | 96 | 42 | 299 | 113 | 0.86 | **0.92*** |
|  |  | Figl A, 2010 |  | 1616 | 926 | 1537 | 833 | 0.95 |  |
|  |  | Santonocito C, 2012 |  | 174 | 160 | 156 | 42 | **0.29*** |  |
|  |  | Povey JE, 2007 |  | 628 | 386 | 541 | 333 | 1.00 |  |
|  |  | Li C, 2006(2) |  | 781 | 423 | 778 | 428 | 1.02 |  |
|  | rs25489 | Hsu L-I, 2015 | G vs. A | 129 | 9 | 365 | 47 | 1.85 | 0.97 |
|  |  | Figl A, 2010 |  | 2403 | 137 | 2213 | 151 | 1.20 |  |
|  |  | Santonocito C, 2012 |  | 238 | 96 | 174 | 24 | **0.34*** |  |

cOR, combined OR; “*” indicates statistical difference (P<0.05).
