# Supplementary material for: The relationship between single nucleotide polymorphisms and skin cancer susceptibility: A systematic review and network meta-analysis
Source: Front Oncol. 2023 Feb 15;13:1094309. doi: 10.3389/fonc.2023.1094309 (PMC9975575; doi:10.3389/fonc.2023.1094309)
Supplement: Supplementary file 3 [file Table_3.docx]

Table 3. The alleles comparisons of enrolled studies in the dominant model

| Gene | SNP | Author, Year | Comparisons | Case | | Control | | OR | cOR |
| --- | --- | --- | --- | --- | --- | --- | --- | --- | --- |
|  |  |  |  | AA+AB | BB | AA+AB | BB |  |  |
| BCL2 | rs2279115 | Oliveira C, 2014 | CC+CA vs. AA | 136 | 64 | 156 | 44 | **0.60*** | - |
| CDKN2B | rs1011970 | Maccioni L, 2013(1) | GG+GT vs. TT | 739 | 31 | 1127 | 19 | **0.40*** | - |
| CYBA | rs1049255 | Yuan T-A, 2018 | CC+CT vs. TT | 124 | 41 | 119 | 20 | **0.51*** | - |
| EIF2S2 | rs6120513 | Maccioni L, 2013(2) | CC+CA vs. AA | 612 | 104 | 837 | 104 | **0.73*** | - |
| EPHX1 | rs1051740 | Hsu L-I, 2015 | CC+CT vs. TT | 61 | 4 | 158 | 31 | **2.99*** | - |
| ERCC1 | rs11615 | Gao R, 2013 | AA+AG vs. GG | 139 | 17 | 86 | 23 | **2.19*** | **1.41*** |
|  |  | Povey JE, 2007 | AA+AG vs. GG | 442 | 65 | 371 | 68 | 1.25 |  |
|  | rs3212948 | Gao R, 2013 | GG+GC vs. CC | 131 | 13 | 75 | 19 | **2.55*** | - |
|  | rs3212950 | Gao R, 2013 | GG+GC vs. CC | 131 | 13 | 77 | 18 | **2.36*** | - |
| ERCC2 | rs238406 | Hsu L-I, 2015 | CC+CA vs. AA | 50 | 17 | 164 | 41 | 0.74 | 0.82 |
|  |  | Povey JE, 2007 | CC+CA vs. AA | 396 | 111 | 347 | 94 | 0.97 |  |
|  |  | Vogel U, 2005 | CC+CA vs. AA | 229 | 90 | 254 | 68 | **0.68*** |  |
| ERCC6 | rs2228527 | Li Y-L, 2017 | AA+AG vs. GG | 598 | 62 | 630 | 32 | **0.49*** | - |
|  | rs2228529 | Li Y-L, 2017 | AA+AG vs. GG | 591 | 71 | 623 | 39 | **0.52*** | - |
| IRF4 | rs12203592 | Pena-Chilet M, 2013(1) | Wt+Ht vs. Ho mut | 519 | 19 | 343 | 2 | **0.16*** | - |
| LOC107987026 | rs2218220 | Maccioni L, 2013(1) | TT+TC vs. CC | 546 | 228 | 888 | 264 | **0.71*** | - |
|  | rs2811710 | Maccioni L, 2013(1) | CC +CT vs. TT | 641 | 132 | 992 | 154 | **0.75*** | - |
|  | rs4636294 | Maccioni L, 2013(1) | GG+GA vs. AA | 544 | 229 | 885 | 268 | **0.72*** | - |
|  | rs751173 | Maccioni L, 2013(1) | TT+TC vs. CC | 595 | 179 | 931 | 221 | **0.79*** | - |
| LURAP1L-AS1 | rs768617 | Fernandez LP, 2008 | CC+CT vs. TT | 130 | 1 | 195 | 50 | **33.33*** | - |
| MC1R | rs1805007 | Motorina AV, 2018 | CC+CT vs. TT | 330 | 1 | 89 | 1 | 3.71 | **0.42*** |
|  |  | Cordoba-Lanus E, 2014 | CC+CT vs. TT | 480 | 1 | 506 | 2 | 1.90 |  |
|  |  | Ozola A, 2019 | CC+CT vs. TT | 254 | 2 | 223 | 1 | 0.57 |  |
|  |  | Helsing P, 2012 | CC+CT vs. TT | 369 | 18 | 412 | 5 | **0.25*** |  |
| MMP1 | rs475007 | Debniak T, 2011 | AA+AT vs. TT | 239 | 56 | 224 | 86 | **1.64*** | 1.05 |
|  |  | Wang L-E, 2011 | AA+AT vs. TT | 677 | 191 | 682 | 171 | 0.89 |  |
|  | rs1144393 | Debniak T, 2011 | AA+AG vs. GG | 243 | 50 | 227 | 92 | **1.97*** | **1.48*** |
|  |  | Wang L-E, 2011 | AA+AG vs. GG | 748 | 115 | 713 | 141 | 1.29 |  |
|  | rs494379 | Debniak T, 2011 | TT+TC vs. CC | 283 | 15 | 256 | 159 | **11.72*** | **2.62*** |
|  |  | Wang L-E, 2011 | TT+TC vs. CC | 813 | 53 | 819 | 36 | 0.67 |  |
|  | rs498186 | Debniak T, 2011 | TT+TG vs. GG | 253 | 41 | 235 | 104 | **2.73*** | **1.35*** |
|  |  | Wang L-E, 2011 | TT+TG vs. GG | 697 | 165 | 685 | 167 | 1.03 |  |
|  | rs514921 | Debniak T, 2011 | AA+AG vs. GG | 277 | 15 | 261 | 162 | **11.46*** | **2.14*** |
|  |  | Wang L-E, 2011 | AA+AG vs. GG | 784 | 84 | 786 | 68 | 0.81 |  |
|  | rs1051121 | Debniak T, 2011 | Wt+Ht vs. Ho mut | 282 | 1 | 268 | 253 | **266.22*** | - |
|  | rs11225426 | Debniak T, 2011 | Wt+Ht vs. Ho mut | 289 | 2 | 271 | 231 | **123.17*** | - |
|  | rs1729376 | Debniak T, 2011 | Wt+Ht vs. Ho mut | 293 | 1 | 275 | 238 | **253.58*** | - |
|  | rs2071230 | Debniak T, 2011 | Wt+Ht vs. Ho mut | 284 | 2 | 266 | 229 | **122.25*** | - |
|  | rs2071231 | Debniak T, 2011 | Wt+Ht vs. Ho mut | 293 | 1 | 270 | 261 | **283.23*** | - |
|  | rs3213460 | Debniak T, 2011 | Wt+Ht vs. Ho mut | 289 | 5 | 274 | 211 | **44.51*** | - |
|  | rs470215 | Debniak T, 2011 | Wt+Ht vs. Ho mut | 256 | 32 | 237 | 116 | **3.92*** | - |
|  | rs470358 | Debniak T, 2011 | Wt+Ht vs. Ho mut | 233 | 47 | 215 | 87 | **2.01*** | - |
|  | rs491152 | Debniak T, 2011 | Wt+Ht vs. Ho mut | 291 | 1 | 272 | 272 | **291.00*** | - |
|  | rs5031036 | Debniak T, 2011 | Wt+Ht vs. Ho mut | 289 | 2 | 261 | 218 | **120.69*** | - |
|  | rs71250626 | Debniak T, 2011 | Wt+Ht vs. Ho mut | 263 | 16 | 242 | 146 | **9.92*** | - |
|  | rs7945189 | Debniak T, 2011 | Wt+Ht vs. Ho mut | 288 | 2 | 269 | 223 | **119.38*** | - |
|  | rs996999 | Debniak T, 2011 | Wt+Ht vs. Ho mut | 281 | 14 | 258 | 164 | **12.76*** | - |
| MTAP | rs10757257 | Maccioni L, 2013(1) | GG+GA vs. AA | 680 | 94 | 974 | 177 | **1.31*** | - |
| MTHFR | rs1801131 | Lesiak A, 2011 | AA +AC vs. CC | 131 | 11 | 139 | 3 | **0.26*** | - |
| near MTAP | rs935053 | Maccioni L, 2013(1) | AA+AG vs. GG | 545 | 228 | 894 | 259 | **0.69*** | - |
| SLC45A2 | rs16891982 | Reis LB, 2020 | GG+GC vs. CC | 119 | 1 | 125 | 10 | **9.52*** | **3.72*** |
|  |  | Guedj M, 2008 | GG+GC vs. CC | 960 | 5 | 1406 | 20 | **2.73*** |  |
|  |  | Fernandez LP, 2008 | GG+GC vs. CC | 130 | 1 | 238 | 7 | 3.82 |  |
|  | rs35388 | Fernandez LP, 2008 | Wt+Ht vs. Ho mut | 115 | 16 | 107 | 138 | **9.27*** | - |
| SOD3 | rs2536512 | Yuan T-A, 2018 | GG+GA vs. AA | 163 | 2 | 102 | 14 | **11.19*** | - |
| SOD2 | rs8031 | Yuan T-A, 2018 | AA+AT vs. TT | 156 | 7 | 102 | 31 | **6.77*** | - |
| STAT3 | rs2293152 | Slawinska M, 2019 | GG+GC vs. CC | 178 | 20 | 178 | 65 | **3.25*** | - |
|  | rs4796793 | Slawinska M, 2019 | CC+CG vs. GG | 179 | 19 | 189 | 54 | **2.69*** | - |
| TERT | rs2853677 | Llorca-Cardenosa MJ, 2014 | TT+TC vs. CC | 583 | 133 | 731 | 213 | 1.28 | - |
| TERT-CLPTM1L | rs401681 | Llorca-Cardenosa MJ, 2014 | CC+CT vs. TT | 580 | 142 | 724 | 232 | 1.31 | - |
| TNF | rs1800629 | Rizzato C, 2011(1) | GG+GA vs. AA | 486 | 20 | 507 | 8 | 0.38 | - |
| VDR | rs2228570 | Pena-Chilet M, 2013(2) | CC+CT vs. TT | 442 | 58 | 270 | 39 | 1.10 | 0.92 |
|  |  | Lesiak A, 2011 | CC+CT vs. TT | 97 | 45 | 125 | 17 | **0.29*** |  |
|  |  | Li C, 2008 | CC+CT vs. TT | 714 | 91 | 740 | 101 | 1.07 |  |
|  |  | Aristizabal-Pachon A, 2022 | CC+CT vs. TT | 109 | 11 | 105 | 15 | 1.42 |  |
| XPC | rs2228001 | Figl A, 2010 | AA+AC vs. CC | 1057 | 216 | 988 | 197 | 0.98 | 0.94 |
|  |  | Li C, 2006(1) | AA+AC vs. CC | 504 | 98 | 506 | 97 | 0.99 |  |
|  |  | Oliveira C, 2013 | AA +AC vs. CC | 124 | 22 | 136 | 10 | **0.41*** |  |
| XRCC1 | rs25487 | Hsu L-I, 2015 | GG+GA vs. AA | 61 | 8 | 185 | 21 | 0.87 | **0.85*** |
|  |  | Figl A, 2010 | GG+GA vs. AA | 1103 | 168 | 1038 | 147 | 0.93 |  |
|  |  | Santonocito C, 2012 | GG+GA vs. AA | 120 | 47 | 97 | 2 | **0.05*** |  |
|  |  | Povey JE, 2007 | GG+GA vs. AA | 430 | 77 | 371 | 66 | 0.99 |  |
|  |  | Li C, 2006(2) | GG+GA vs. AA | 525 | 77 | 529 | 74 | 0.95 |  |
|  | rs25489 | Hsu L-I, 2015 | GG+GA vs. AA | 69 | 1 | 202 | 4 | 1.37 | 0.93 |
|  |  | Figl A, 2010 | GG+GA vs. AA | 1266 | 4 | 1165 | 17 | **4.62*** |  |
|  |  | Santonocito C, 2012 | GG+GA vs. AA | 144 | 23 | 98 | 1 | **0.06*** |  |

cOR, combined OR; Wt, wildtype; Ht, heterozygous; Ho mut, mutated homozygous; “*” indicates statistical difference (P<0.05).
