# Supplementary material for: The relationship between single nucleotide polymorphisms and skin cancer susceptibility: A systematic review and network meta-analysis
Source: Front Oncol. 2023 Feb 15;13:1094309. doi: 10.3389/fonc.2023.1094309 (PMC9975575; doi:10.3389/fonc.2023.1094309)
Supplement: Supplementary file 4 [file Table_4.docx]

Table 4. The direct and indirect evidence of each compairson in the subgroup one

| Comparison | Direct | Indirect | Diff | Z | p-value |
| --- | --- | --- | --- | --- | --- |
| rs1544410 vs rs2228570 | 0.05 | 0.00 | 0.05 | 0.26 | 0.79 |
| rs1544410 vs rs731236 | -0.03 | -0.04 | 0.01 | 0.04 | 0.97 |
| rs2228570 vs rs731236 | -0.08 | -0.25 | 0.17 | 0.41 | 0.68 |

Table 4. The direct and indirect evidence of each compairson in the subgroup two

| Comparison | Direct | Indirect | Diff | Z | p-value |
| --- | --- | --- | --- | --- | --- |
| rs1042522 vs rs1136410 | . | -0.81 | . | . | . |
| rs1042522 vs rs11615 | 0.36 | . | . | . | . |
| rs1042522 vs rs13181 | 0.34 | 1.08 | -0.74 | -3.30 | **0.00*** |
| rs1042522 vs rs1695 | . | -0.21 | . | . | . |
| rs1042522 vs rs1799793 | . | 0.46 | . | . | . |
| rs1042522 vs rs1805006 | . | -3.11 | . | . | . |
| rs1042522 vs rs1805007 | -1.55 | -0.27 | -1.28 | -3.83 | **0.00*** |
| rs1042522 vs rs1805008 | -1.02 | -2.48 | 1.46 | 3.83 | **0.00*** |
| rs1042522 vs rs25487 | 0.58 | -0.18 | 0.76 | 3.30 | **0.00*** |
| rs1042522 vs rs25489 | . | -1.45 | . | . | . |
| rs1042522 vs rs4911414 | . | -0.25 | . | . | . |
| rs1042522 vs rs885479 | . | -2.10 | . | . | . |
| rs1136410 vs rs11615 | . | 1.17 | . | . | . |
| rs1136410 vs rs13181 | . | 1.29 | . | . | . |
| rs1136410 vs rs1695 | . | 0.60 | . | . | . |
| rs1136410 vs rs1799793 | . | 1.28 | . | . | . |
| rs1136410 vs rs1805006 | . | -2.30 | . | . | . |
| rs1136410 vs rs1805007 | . | -0.33 | . | . | . |
| rs1136410 vs rs1805008 | . | -0.48 | . | . | . |
| rs1136410 vs rs25487 | 1.12 | 4.79 | -3.67 | -8.50 < | **0.00*** |
| rs1136410 vs rs25489 | 0.33 | -1.21 | 1.54 | 6.87 < | **0.00*** |
| rs1136410 vs rs4911414 | . | 0.57 | . | . | . |
| rs1136410 vs rs885479 | . | -1.29 | . | . | . |
| rs11615 vs rs13181 | -0.02 | 0.68 | -0.70 | -3.30 | **0.00*** |
| rs11615 vs rs1695 | . | -0.57 | . | . | . |
| rs11615 vs rs1799793 | . | 0.11 | . | . | . |
| rs11615 vs rs1805006 | . | -3.47 | . | . | . |
| rs11615 vs rs1805007 | . | -1.50 | . | . | . |
| rs11615 vs rs1805008 | . | -1.65 | . | . | . |
| rs11615 vs rs25487 | 0.22 | -0.50 | 0.73 | 3.30 | **0.00*** |
| rs11615 vs rs25489 | . | -1.81 | . | . | . |
| rs11615 vs rs4911414 | . | -0.61 | . | . | . |
| rs11615 vs rs885479 | . | -2.46 | . | . | . |
| rs13181 vs rs1695 | . | -0.69 | . | . | . |
| rs13181 vs rs1799793 | -0.03 | 0.68 | -0.71 | -3.30 | **0.00*** |
| rs13181 vs rs1805006 | . | -3.59 | . | . | . |
| rs13181 vs rs1805007 | . | -1.62 | . | . | . |
| rs13181 vs rs1805008 | . | -1.77 | . | . | . |
| rs13181 vs rs25487 | 0.04 | -0.39 | 0.43 | 3.36 | **0.00*** |
| rs13181 vs rs25489 | -2.35 | -0.95 | -1.40 | -7.88 | **0.00*** |
| rs13181 vs rs4911414 | . | -0.73 | . | . | . |
| rs13181 vs rs885479 | . | -2.58 | . | . | . |
| rs1695 vs rs1799793 | . | 0.68 | . | . | . |
| rs1695 vs rs1805006 | . | -2.89 | . | . | . |
| rs1695 vs rs1805007 | . | -0.93 | . | . | . |
| rs1695 vs rs1805008 | . | -1.08 | . | . | . |
| rs1695 vs rs25487 | 0.65 | 0.83 | -0.17 | -0.18 | 0.86 |
| rs1695 vs rs25489 | -1.18 | -1.29 | 0.10 | 0.18 | 0.86 |
| rs1695 vs rs4911414 | . | -0.03 | . | . | . |
| rs1695 vs rs885479 | . | -1.89 | . | . | . |
| rs1799793 vs rs1805006 | . | -3.57 | . | . | . |
| rs1799793 vs rs1805007 | . | -1.61 | . | . | . |
| rs1799793 vs rs1805008 | . | -1.76 | . | . | . |
| rs1799793 vs rs25487 | 0.01 | -0.06 | 0.07 | 0.68 | 0.50 |
| rs1799793 vs rs25489 | -2.30 | -0.97 | -1.32 | -7.30 < | **0.00*** |
| rs1799793 vs rs4911414 | . | -0.71 | . | . | . |
| rs1799793 vs rs885479 | . | -2.57 | . | . | . |
| rs1805007 vs rs1805006 | -2.07 | 3.42 | -5.49 | -3.34 | **0.00*** |
| rs1805008 vs rs1805006 | -1.65 | -5.26 | 3.60 | 3.37 | **0.00*** |
| rs25487 vs rs1805006 | . | -3.56 | . | . | . |
| rs25489 vs rs1805006 | . | -1.66 | . | . | . |
| rs4911414 vs rs1805006 | -2.94 | -2.35 | -0.59 | -0.92 | 0.36 |
| rs885479 vs rs1805006 | -1.01 | . | . | . | . |
| rs1805007 vs rs1805008 | -0.16 | 2.25 | -2.41 | -1.28 | 0.20 |
| rs1805007 vs rs25487 | . | 1.59 | . | . | . |
| rs1805007 vs rs25489 | . | -0.31 | . | . | . |
| rs1805007 vs rs4911414 | 0.80 | 1.65 | -0.85 | -2.37 | **0.02*** |
| rs1805007 vs rs885479 | -1.06 | 1.54 | -2.60 | -3.27 | **0.00*** |
| rs1805008 vs rs25487 | . | 1.74 | . | . | . |
| rs1805008 vs rs25489 | . | -0.16 | . | . | . |
| rs1805008 vs rs4911414 | 1.12 | 0.51 | 0.61 | 1.74 | 0.08 |
| rs1805008 vs rs885479 | -0.67 | -2.71 | 2.04 | 3.37 | **0.00*** |
| rs25487 vs rs25489 | -1.92 | -1.73 | -0.19 | -0.54 | 0.59 |
| rs25487 vs rs4911414 | . | -0.70 | . | . | . |
| rs25487 vs rs885479 | . | -2.56 | . | . | . |
| rs25489 vs rs4911414 | . | 1.21 | . | . | . |
| rs25489 vs rs885479 | . | -0.65 | . | . | . |
| rs4911414 vs rs885479 | -1.89 | -1.47 | -0.42 | -0.78 | 0.4331 |

“*” indicates statistical difference (P<0.05).
