# Supplementary material for: The relationship between single nucleotide polymorphisms and skin cancer susceptibility: A systematic review and network meta-analysis
Source: Front Oncol. 2023 Feb 15;13:1094309. doi: 10.3389/fonc.2023.1094309 (PMC9975575; doi:10.3389/fonc.2023.1094309)
Supplement: Supplementary file 5 [file Table_5.docx]

Table 5. The direct and indirect evidence of each compairson in the subgroup one

| comparison | direct | indirect | Diff | z | p-value |
| --- | --- | --- | --- | --- | --- |
| rs11225426 vs rs1051121 | -0.67 | . | . | . | . |
| rs1144393 vs rs1051121 | -4.06 | 7.35 | -11.41 | -1.70 | 0.09 |
| rs1729376 vs rs1051121 | 0.04 | . | . | . | . |
| rs2071230 vs rs1051121 | -0.69 | . | . | . | . |
| rs2071231 vs rs1051121 | 0.04 | . | . | . | . |
| rs3213460 vs rs1051121 | -1.58 | . | . | . | . |
| rs470215 vs rs1051121 | -3.56 | . | . | . | . |
| rs470358 vs rs1051121 | -4.04 | . | . | . | . |
| rs475007 vs rs1051121 | -4.19 | -3.81 | -0.38 | -0.06 | 0.96 |
| rs491152 vs rs1051121 | 0.03 | . | . | . | . |
| rs494379 vs rs1051121 | -2.70 | -2.77 | 0.07 | 0.01 | 0.99 |
| rs498186 vs rs1051121 | -3.82 | -7.66 | 3.84 | 0.60 | 0.55 |
| rs5031036 vs rs1051121 | -0.67 | . | . | . | . |
| rs514921 vs rs1051121 | -2.73 | -8.80 | 6.07 | 1.31 | 0.19 |
| rs71250626 vs rs1051121 | -2.84 | . | . | . | . |
| rs7945189 vs rs1051121 | -0.67 | . | . | . | . |
| rs996999 vs rs1051121 | -2.64 | . | . | . | . |
| rs11225426 vs rs1144393 | 3.39 | -2.84 | 6.23 | 1.70 | 0.09 |
| rs11225426 vs rs1729376 | -0.71 | . | . | . | . |
| rs11225426 vs rs2071230 | 0.02 | . | . | . | . |
| rs11225426 vs rs2071231 | -0.71 | . | . | . | . |
| rs11225426 vs rs3213460 | 0.92 | . | . | . | . |
| rs11225426 vs rs470215 | 2.89 | . | . | . | . |
| rs11225426 vs rs470358 | 3.37 | . | . | . | . |
| rs11225426 vs rs475007 | 3.52 | 3.31 | 0.21 | 0.06 | 0.96 |
| rs11225426 vs rs491152 | -0.70 | . | . | . | . |
| rs11225426 vs rs494379 | 2.04 | 2.07 | -0.04 | -0.01 | 0.99 |
| rs11225426 vs rs498186 | 3.15 | 5.26 | -2.11 | -0.60 | 0.55 |
| rs11225426 vs rs5031036 | 0.00 | . | . | . | . |
| rs11225426 vs rs514921 | 2.06 | 5.49 | -3.43 | -1.31 | 0.19 |
| rs11225426 vs rs71250626 | 2.17 | . | . | . | . |
| rs11225426 vs rs7945189 | 0.00 | . | . | . | . |
| rs11225426 vs rs996999 | 1.97 | . | . | . | . |
| rs1144393 vs rs1729376 | -4.10 | 7.31 | -11.41 | -1.70 | 0.09 |
| rs1144393 vs rs2071230 | -3.37 | 2.86 | -6.24 | -1.70 | 0.09 |
| rs1144393 vs rs2071231 | -4.10 | 7.31 | -11.41 | -1.70 | 0.09 |
| rs1144393 vs rs3213460 | -2.48 | 0.66 | -3.13 | -1.70 | 0.09 |
| rs1144393 vs rs470215 | -0.50 | 0.89 | -1.39 | -1.70 | 0.09 |
| rs1144393 vs rs470358 | -0.02 | 1.27 | -1.29 | -1.70 | 0.09 |
| rs1144393 vs rs475007 | 0.40 | . | . | . | . |
| rs1144393 vs rs491152 | -4.09 | 7.31 | -11.41 | -1.70 | 0.09 |
| rs1144393 vs rs494379 | -1.05 | -6.43 | 5.38 | 1.46 | 0.15 |
| rs1144393 vs rs498186 | 0.15 | . | . | . | . |
| rs1144393 vs rs5031036 | -3.39 | 2.84 | -6.23 | -1.70 | 0.09 |
| rs1144393 vs rs514921 | -0.72 | -2.01 | 1.30 | 0.48 | 0.63 |
| rs1144393 vs rs71250626 | -1.22 | 0.49 | -1.71 | -1.70 | 0.09 |
| rs1144393 vs rs7945189 | -3.39 | 2.85 | -6.23 | -1.70 | 0.09 |
| rs1144393 vs rs996999 | -1.42 | 0.39 | -1.80 | -1.70 | 0.09 |
| rs1729376 vs rs2071230 | 0.72 | . | . | . | . |
| rs1729376 vs rs2071231 | 0.00 | . | . | . | . |
| rs1729376 vs rs3213460 | 1.62 | . | . | . | . |
| rs1729376 vs rs470215 | 3.60 | . | . | . | . |
| rs1729376 vs rs470358 | 4.08 | . | . | . | . |
| rs1729376 vs rs475007 | 4.23 | 3.84 | 0.38 | 0.06 | 0.96 |
| rs1729376 vs rs491152 | 0.01 | . | . | . | . |
| rs1729376 vs rs494379 | 2.74 | 2.81 | -0.07 | -0.01 | 0.99 |
| rs1729376 vs rs498186 | 3.86 | 7.70 | -3.84 | -0.60 | 0.55 |
| rs1729376 vs rs5031036 | 0.71 | . | . | . | . |
| rs1729376 vs rs514921 | 2.76 | 8.84 | -6.07 | -1.31 | 0.19 |
| rs1729376 vs rs71250626 | 2.88 | . | . | . | . |
| rs1729376 vs rs7945189 | 0.71 | . | . | . | . |
| rs1729376 vs rs996999 | 2.68 | . | . | . | . |
| rs2071230 vs rs2071231 | -0.72 | . | . | . | . |
| rs2071230 vs rs3213460 | 0.90 | . | . | . | . |
| rs2071230 vs rs470215 | 2.88 | . | . | . | . |
| rs2071230 vs rs470358 | 3.35 | . | . | . | . |
| rs2071230 vs rs475007 | 3.50 | 3.29 | 0.21 | 0.06 | 0.96 |
| rs2071230 vs rs491152 | -0.72 | . | . | . | . |
| rs2071230 vs rs494379 | 2.02 | 2.06 | -0.04 | -0.01 | 0.99 |
| rs2071230 vs rs498186 | 3.14 | 5.24 | -2.11 | -0.60 | 0.55 |
| rs2071230 vs rs5031036 | -0.02 | . | . | . | . |
| rs2071230 vs rs514921 | 2.04 | 5.47 | -3.43 | -1.31 | 0.19 |
| rs2071230 vs rs71250626 | 2.16 | . | . | . | . |
| rs2071230 vs rs7945189 | -0.01 | . | . | . | . |
| rs2071230 vs rs996999 | 1.96 | . | . | . | . |
| rs2071231 vs rs3213460 | 1.62 | . | . | . | . |
| rs2071231 vs rs470215 | 3.60 | . | . | . | . |
| rs2071231 vs rs470358 | 4.08 | . | . | . | . |
| rs2071231 vs rs475007 | 4.23 | 3.84 | 0.38 | 0.06 | 0.96 |
| rs2071231 vs rs491152 | 0.01 | . | . | . | . |
| rs2071231 vs rs494379 | 2.74 | 2.81 | -0.07 | -0.01 | 0.99 |
| rs2071231 vs rs498186 | 3.86 | 7.70 | -3.84 | -0.60 | 0.55 |
| rs2071231 vs rs5031036 | 0.71 | . | . | . | . |
| rs2071231 vs rs514921 | 2.76 | 8.84 | -6.07 | -1.31 | 0.19 |
| rs2071231 vs rs71250626 | 2.88 | . | . | . | . |
| rs2071231 vs rs7945189 | 0.71 | . | . | . | . |
| rs2071231 vs rs996999 | 2.68 | . | . | . | . |
| rs3213460 vs rs470215 | 1.98 | . | . | . | . |
| rs3213460 vs rs470358 | 2.46 | . | . | . | . |
| rs3213460 vs rs475007 | 2.61 | 2.50 | 0.11 | 0.06 | 0.96 |
| rs3213460 vs rs491152 | -1.62 | . | . | . | . |
| rs3213460 vs rs494379 | 1.12 | 1.14 | -0.02 | -0.01 | 0.99 |
| rs3213460 vs rs498186 | 2.24 | 3.30 | -1.06 | -0.60 | 0.55 |
| rs3213460 vs rs5031036 | -0.92 | . | . | . | . |
| rs3213460 vs rs514921 | 1.14 | 2.99 | -1.84 | -1.31 | 0.19 |
| rs3213460 vs rs71250626 | 1.26 | . | . | . | . |
| rs3213460 vs rs7945189 | -0.91 | . | . | . | . |
| rs3213460 vs rs996999 | 1.06 | . | . | . | . |
| rs470215 vs rs470358 | 0.48 | . | . | . | . |
| rs470215 vs rs475007 | 0.63 | 0.58 | 0.05 | 0.06 | 0.96 |
| rs470215 vs rs491152 | -3.59 | . | . | . | . |
| rs470215 vs rs494379 | -0.86 | -0.85 | -0.01 | -0.01 | 0.99 |
| rs470215 vs rs498186 | 0.26 | 0.74 | -0.48 | -0.60 | 0.55 |
| rs470215 vs rs5031036 | -2.89 | . | . | . | . |
| rs470215 vs rs514921 | -0.84 | 0.12 | -0.96 | -1.31 | 0.19 |
| rs470215 vs rs71250626 | -0.72 | . | . | . | . |
| rs470215 vs rs7945189 | -2.89 | . | . | . | . |
| rs470215 vs rs996999 | -0.92 | . | . | . | . |
| rs470358 vs rs475007 | 0.15 | 0.11 | 0.04 | 0.06 | 0.96 |
| rs470358 vs rs491152 | -4.07 | . | . | . | . |
| rs470358 vs rs494379 | -1.34 | -1.33 | -0.01 | -0.01 | 0.99 |
| rs470358 vs rs498186 | -0.22 | 0.23 | -0.45 | -0.60 | 0.55 |
| rs470358 vs rs5031036 | -3.37 | . | . | . | . |
| rs470358 vs rs514921 | -1.32 | -0.41 | -0.90 | -1.31 | 0.19 |
| rs470358 vs rs71250626 | -1.20 | . | . | . | . |
| rs470358 vs rs7945189 | -3.37 | . | . | . | . |
| rs470358 vs rs996999 | -1.40 | . | . | . | . |
| rs475007 vs rs491152 | -4.22 | -3.84 | -0.38 | -0.06 | 0.96 |
| rs475007 vs rs494379 | -1.47 | -1.63 | 0.16 | 0.04 | 0.97 |
| rs475007 vs rs498186 | -0.26 | . | . | . | . |
| rs475007 vs rs5031036 | -3.52 | -3.31 | -0.21 | -0.06 | 0.96 |
| rs475007 vs rs514921 | -1.15 | 1.58 | -2.73 | -0.93 | 0.35 |
| rs475007 vs rs71250626 | -1.35 | -1.29 | -0.06 | -0.06 | 0.96 |
| rs475007 vs rs7945189 | -3.52 | -3.31 | -0.21 | -0.06 | 0.96 |
| rs475007 vs rs996999 | -1.55 | -1.49 | -0.06 | -0.06 | 0.96 |
| rs491152 vs rs494379 | 2.74 | 2.80 | -0.07 | -0.01 | 0.99 |
| rs491152 vs rs498186 | 3.85 | 7.69 | -3.84 | -0.60 | 0.55 |
| rs491152 vs rs5031036 | 0.70 | . | . | . | . |
| rs491152 vs rs514921 | 2.76 | 8.83 | -6.07 | -1.31 | 0.19 |
| rs491152 vs rs71250626 | 2.87 | . | . | . | . |
| rs491152 vs rs7945189 | 0.70 | . | . | . | . |
| rs491152 vs rs996999 | 2.67 | . | . | . | . |
| rs494379 vs rs498186 | 1.23 | -1.71 | 2.93 | 0.53 | 0.60 |
| rs494379 vs rs5031036 | -2.04 | -2.07 | 0.04 | 0.01 | 0.99 |
| rs494379 vs rs514921 | 0.34 | 11.52 | -11.18 | -1.05 | 0.29 |
| rs494379 vs rs71250626 | 0.14 | 0.13 | 0.01 | 0.01 | 0.99 |
| rs494379 vs rs7945189 | -2.03 | -2.07 | 0.04 | 0.01 | 0.99 |
| rs494379 vs rs996999 | -0.06 | -0.07 | 0.01 | 0.01 | 0.99 |
| rs498186 vs rs5031036 | -3.15 | -5.26 | 2.11 | 0.60 | 0.55 |
| rs498186 vs rs514921 | -0.90 | 4.47 | -5.38 | -1.50 | 0.13 |
| rs498186 vs rs71250626 | -0.98 | -1.57 | 0.59 | 0.60 | 0.55 |
| rs498186 vs rs7945189 | -3.15 | -5.26 | 2.11 | 0.60 | 0.55 |
| rs498186 vs rs996999 | -1.18 | -1.80 | 0.62 | 0.60 | 0.55 |
| rs5031036 vs rs514921 | 2.06 | 5.49 | -3.43 | -1.31 | 0.19 |
| rs5031036 vs rs71250626 | 2.17 | . | . | . | . |
| rs5031036 vs rs7945189 | 0.00 | . | . | . | . |
| rs5031036 vs rs996999 | 1.97 | . | . | . | . |
| rs514921 vs rs71250626 | 0.12 | -1.00 | 1.12 | 1.31 | 0.19 |
| rs514921 vs rs7945189 | -2.05 | -5.48 | 3.43 | 1.31 | 0.19 |
| rs514921 vs rs996999 | -0.08 | -1.25 | 1.17 | 1.31 | 0.19 |
| rs71250626 vs rs7945189 | -2.17 | . | . | . | . |
| rs71250626 vs rs996999 | -0.20 | . | . | . | . |
| rs7945189 vs rs996999 | 1.97 | . | . | . | . |

Table 2. The direct and indirect evidence of each compairson in the subgroup two

| comparison | direct | indirect | Diff | z | p-value |
| --- | --- | --- | --- | --- | --- |
| rs1051740 vs rs11615 | . | 0.78 | . | . | . |
| rs1051740 vs rs2228001 | . | 1.29 | . | . | . |
| rs1051740 vs rs238406 | 1.65 | -0.87 | 2.51 | 1.20 | 0.23 |
| rs1051740 vs rs25487 | 0.69 | 1.86 | -1.17 | -0.83 | 0.41 |
| rs1051740 vs rs25489 | -1.51 | -0.39 | -1.12 | -0.83 | 0.40 |
| rs1051740 vs rs3212948 | . | 0.57 | . | . | . |
| rs1051740 vs rs3212950 | . | 0.57 | . | . | . |
| rs11615 vs rs2228001 | . | 0.51 | . | . | . |
| rs11615 vs rs238406 | 0.65 | 2.10 | -1.45 | -1.20 | 0.23 |
| rs11615 vs rs25487 | 0.20 | -1.04 | 1.24 | 1.20 | 0.23 |
| rs11615 vs rs25489 | . | -1.49 | . | . | . |
| rs11615 vs rs3212948 | -0.21 | . | . | . | . |
| rs11615 vs rs3212950 | -0.21 | . | . | . | . |
| rs2228001 vs rs238406 | . | 0.16 | . | . | . |
| rs2228001 vs rs25487 | -0.29 | -5.49 | 5.20 | 4.98 | **0.00*** |
| rs2228001 vs rs25489 | -4.17 | -1.24 | -2.93 | -4.98 | **0.00*** |
| rs2228001 vs rs3212948 | . | -0.72 | . | . | . |
| rs2228001 vs rs3212950 | . | -0.72 | . | . | . |
| rs238406 vs rs25487 | -0.50 | -1.89 | 1.38 | 0.83 | 0.40 |
| rs238406 vs rs25489 | -3.16 | -2.09 | -1.07 | -0.98 | 0.33 |
| rs238406 vs rs3212948 | . | -0.88 | . | . | . |
| rs238406 vs rs3212950 | . | -0.88 | . | . | . |
| rs25487 vs rs25489 | -1.63 | -6.33 | 4.69 | 1.20 | 0.23 |
| rs25487 vs rs3212948 | . | -0.37 | . | . | . |
| rs25487 vs rs3212950 | . | -0.37 | . | . | . |
| rs25489 vs rs3212948 | . | 1.28 | . | . | . |
| rs25489 vs rs3212950 | . | 1.28 | . | . | . |
| rs3212950 vs rs3212948 | 0.00 | . | . | . | . |

“*” indicates statistical difference (P<0.05).
