# Supplementary material for: The relationship between single nucleotide polymorphisms and skin cancer susceptibility: A systematic review and network meta-analysis
Source: Front Oncol. 2023 Feb 15;13:1094309. doi: 10.3389/fonc.2023.1094309 (PMC9975575; doi:10.3389/fonc.2023.1094309)
Supplement: Supplementary file 6 [file Image_1.pdf]

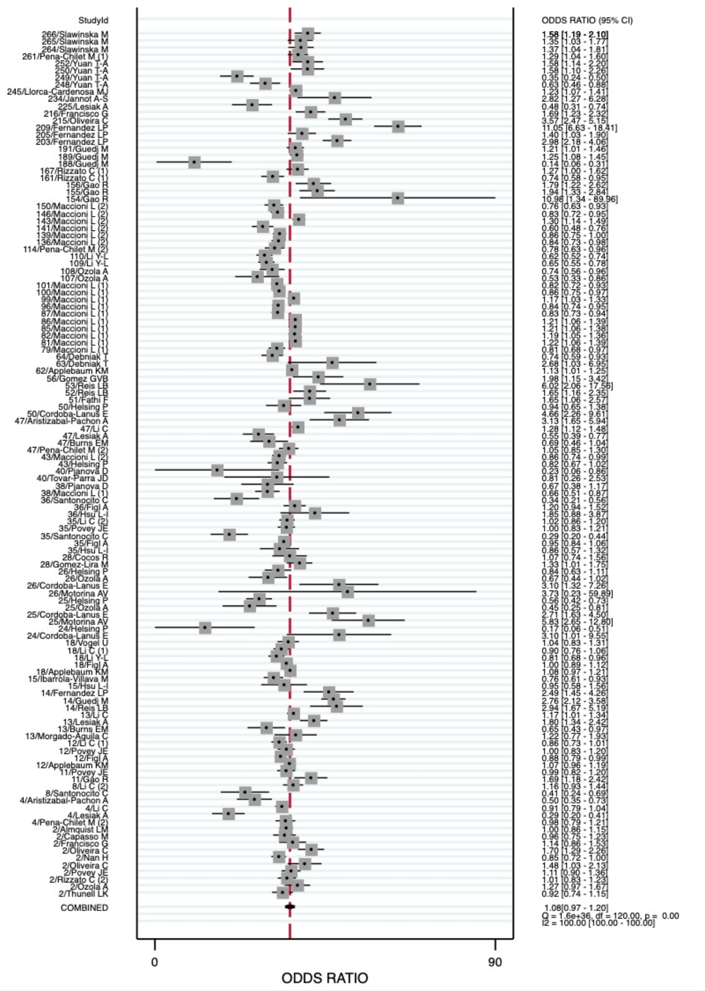

a. The Allele model (A vs. B)

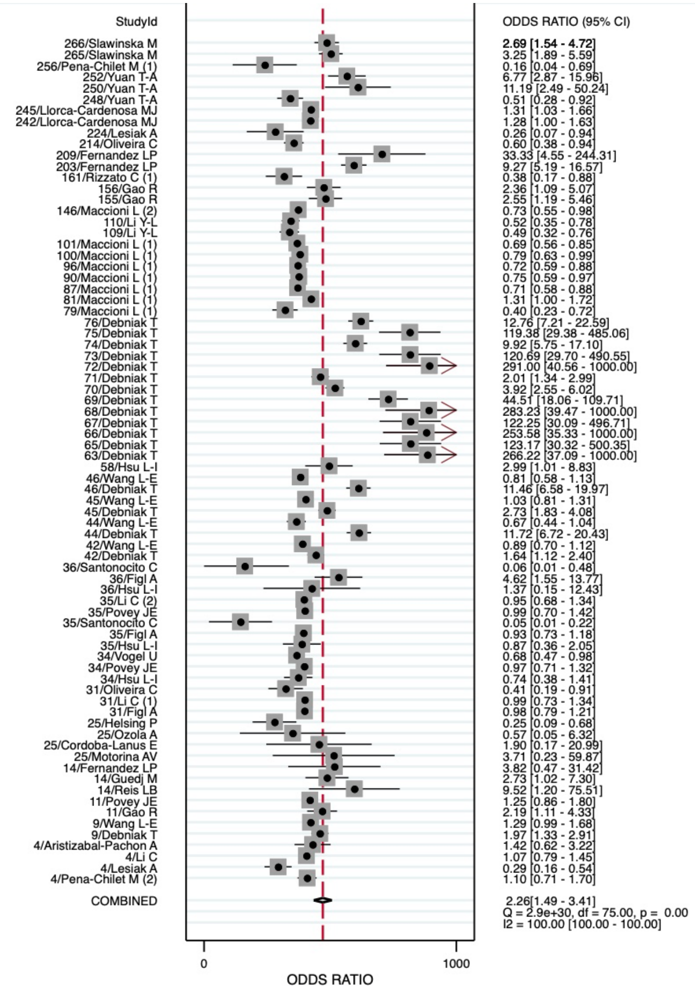

b. The Dominant model (AA+AB vs. BB)

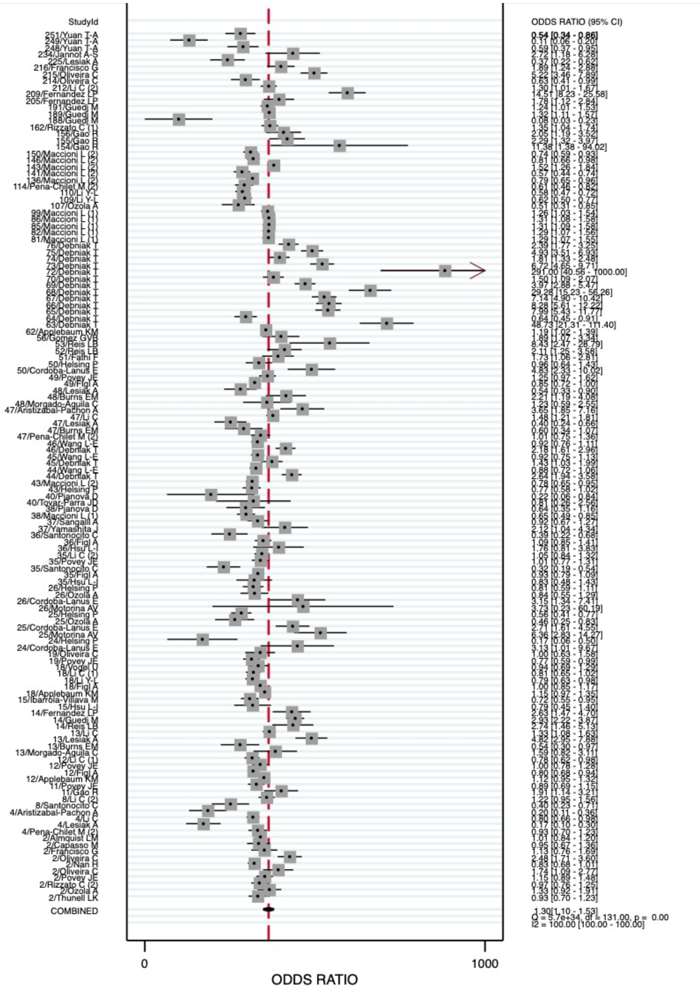

c. The Recessive model (AA vs. AB+BB)

Figure 1. Odds ratio forest plot. The dots represent the value of the diagnostic odds ratio for each study, the length of the horizontal line represents the width of the confidence interval, the left end of the horizontal line is the lowest value of the confidence interval, the right end is the highest value, and the value in brackets is the diagnostic odds ratio 95 % confidence intervals, and the diamonds are the pooled effect sizes of the diagnostic odds ratios.
