# Supplementary material for: The relationship between single nucleotide polymorphisms and skin cancer susceptibility: A systematic review and network meta-analysis
Source: Front Oncol. 2023 Feb 15;13:1094309. doi: 10.3389/fonc.2023.1094309 (PMC9975575; doi:10.3389/fonc.2023.1094309)
Supplement: Supplementary file 7 [file Image_2.pdf]

Figure 2. The percentage of direct and indirect evidence in the subgroup two of the allele model

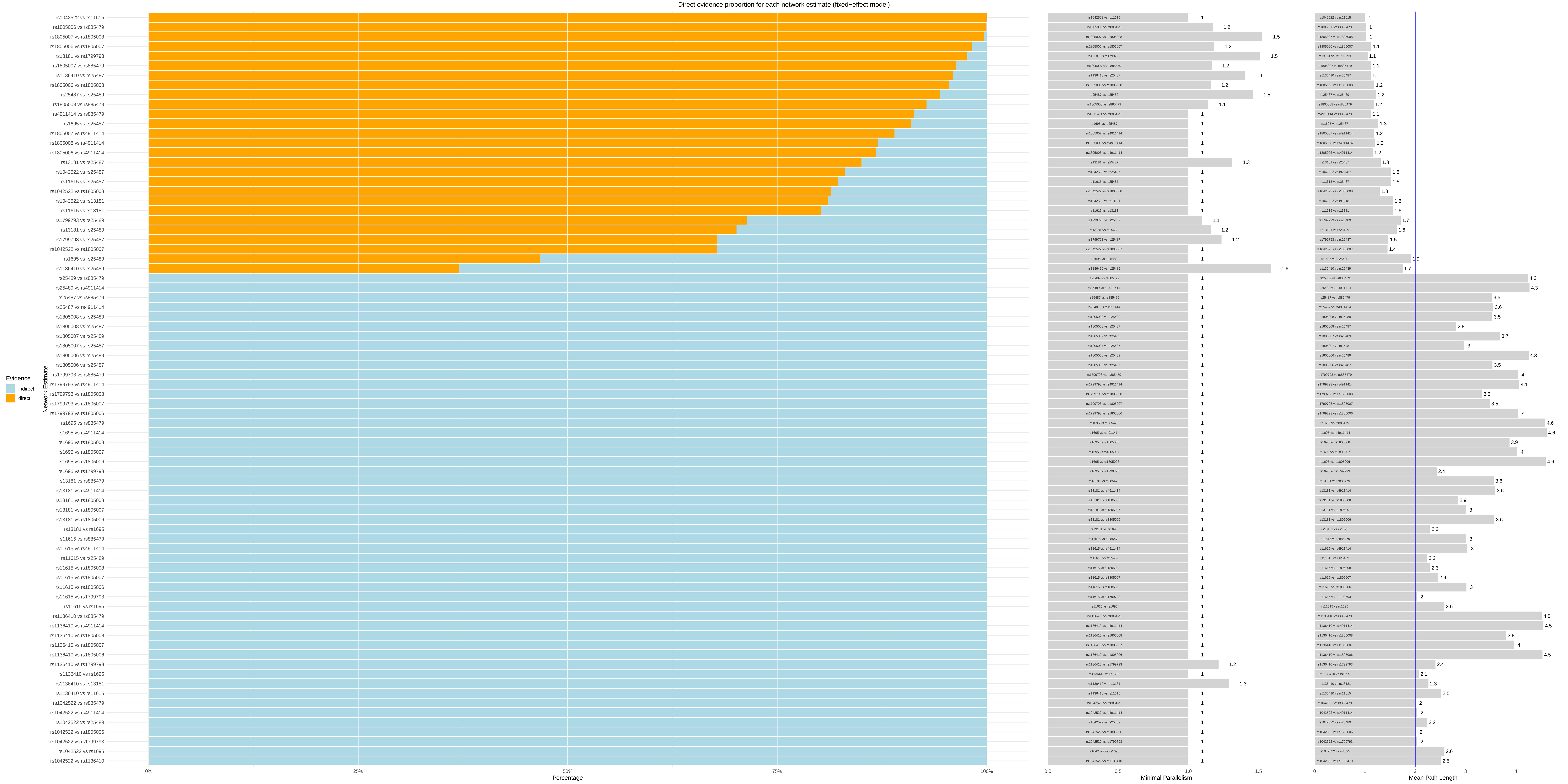

Direct evidence proportion for each network estimate (fixed−effect model)
rs1042522 vs rs11615
rs1805006 vs rs885479
rs1805007 vs rs1805008
rs1805006 vs rs1805007
rs13181 vs rs1799793
rs1805007 vs rs885479
rs1136410 vs rs25487
rs1805006 vs rs1805008
rs25487 vs rs25489
rs1805008 vs rs885479
rs4911414 vs rs885479
rs1695 vs rs25487
rs1805007 vs rs4911414
rs1805008 vs rs4911414
rs1805006 vs rs4911414
rs13181 vs rs25487
rs1042522 vs rs25487
rs11615 vs rs25487
rs1042522 vs rs1805008
rs1042522 vs rs13181
rs11615 vs rs13181
rs1799793 vs rs25489
rs13181 vs rs25489
rs1799793 vs rs25487
rs1042522 vs rs1805007
rs1695 vs rs25489
rs1136410 vs rs25489
rs25489 vs rs885479
rs25489 vs rs4911414
rs25487 vs rs885479
rs25487 vs rs4911414
rs1805008 vs rs25489
rs1805008 vs rs25487
rs1805007 vs rs25489
rs1805007 vs rs25487
rs1805006 vs rs25489
rs1805006 vs rs25487
rs1799793 vs rs885479
rs1799793 vs rs4911414
rs1799793 vs rs1805008
rs1799793 vs rs1805007
rs1799793 vs rs1805006
rs1695 vs rs885479
rs1695 vs rs4911414
rs1695 vs rs1805008
rs1695 vs rs1805007
rs1695 vs rs1805006
rs1695 vs rs1799793
rs13181 vs rs885479
rs13181 vs rs4911414
rs13181 vs rs1805008
rs13181 vs rs1805007
rs13181 vs rs1805006
rs13181 vs rs1695
rs11615 vs rs885479
rs11615 vs rs4911414
rs11615 vs rs25489
rs11615 vs rs1805008
rs11615 vs rs1805007
rs11615 vs rs1805006
rs11615 vs rs1799793
rs11615 vs rs1695
rs1136410 vs rs885479
rs1136410 vs rs4911414
rs1136410 vs rs1805008
rs1136410 vs rs1805007
rs1136410 vs rs1805006
rs1136410 vs rs1799793
rs1136410 vs rs1695
rs1136410 vs rs13181
rs1136410 vs rs11615
rs1042522 vs rs885479
rs1042522 vs rs4911414
rs1042522 vs rs25489
rs1042522 vs rs1805006
rs1042522 vs rs1799793
rs1042522 vs rs1695
rs1042522 vs rs1136410
Network Estimate
Evidence
indirect
direct
0%
25%
50%
75%
100%
Percentage
1 1.2 1.5 1.2 1.5 1.2 1.4 1.2 1.5 1.1 1 1 1 1 1 1.3 1 1 1 1 1 1.1 1.2 1.2 1 1 1.6 1 1 1 1 1 1 1 1 1 1 1 1 1 1 1 1 1 1 1 1 1 1 1 1 1 1 1 1 1 1 1 1 1 1 1 1 1 1 1 1 1.2 1 1.3 1 1 1 1 1 1 1 1
0.0
0.5
1.0
1.5
Minimal Parallelism
1 1 1 1.1 1.1 1.1 1.1 1.2 1.2 1.2 1.1 1.3 1.2 1.2 1.2 1.3 1.5 1.5 1.3 1.6 1.6 1.7 1.6 1.5 1.4 1.9 1.7 4.2 4.3 3.5 3.6 3.5 2.8 3.7 3 4.3 3.5 4 4.1 3.3 3.5 4 4.6 4.6 3.9 4 4.6 2.4 3.6 3.6 2.9 3 3.6 2.3 3 3 2.2 2.3 2.4 3 2 2.6 4.5 4.5 3.8 4 4.5 2.4 2.1 2.3 2.5 2 2 2.2 2 2 2.6 2.5
0
1
2
3
4
Mean Path Length
