# Supplementary material for: The relationship between single nucleotide polymorphisms and skin cancer susceptibility: A systematic review and network meta-analysis
Source: Front Oncol. 2023 Feb 15;13:1094309. doi: 10.3389/fonc.2023.1094309 (PMC9975575; doi:10.3389/fonc.2023.1094309)

Figure 3. The direct evidence proportion in the subgroup one of the dominant model

Direct evidence proportion for each network estimate (fixed−effect model)

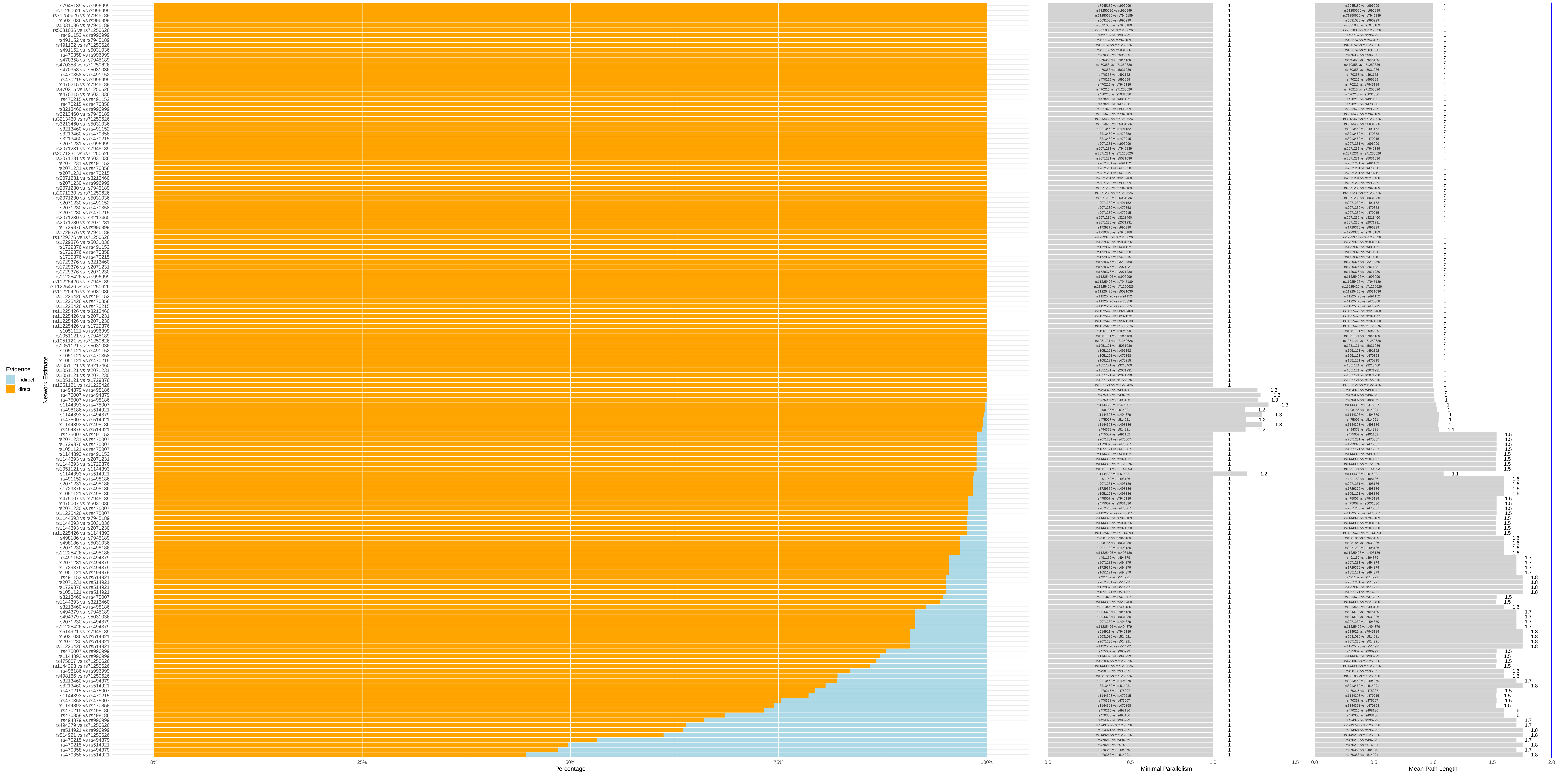

Supplement: Supplementary file 8 [file Image_3.pdf]
