# Supplementary material for: The relationship between single nucleotide polymorphisms and skin cancer susceptibility: A systematic review and network meta-analysis
Source: Front Oncol. 2023 Feb 15;13:1094309. doi: 10.3389/fonc.2023.1094309 (PMC9975575; doi:10.3389/fonc.2023.1094309)
Supplement: Supplementary file 9 [file Image_4.pdf]

Figure 4. The direct evidence proportion in the subgroup two of the dominant model

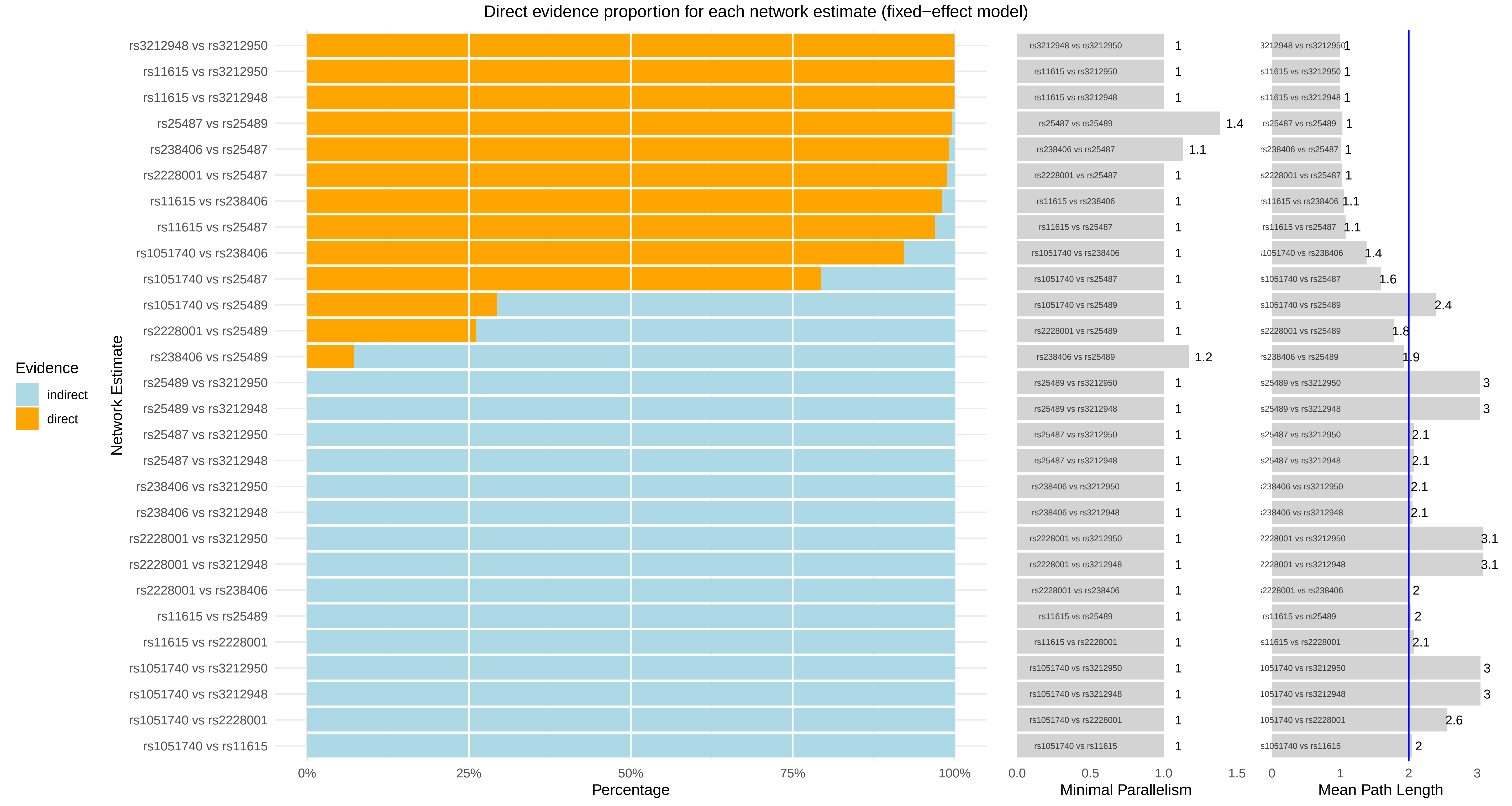

Direct evidence proportion for each network estimate (fixed−effect model)
Evidence
indirect
direct
Network Estimate
rs3212948 vs rs3212950
rs11615 vs rs3212950
rs11615 vs rs3212948
rs25487 vs rs25489
rs238406 vs rs25487
rs2228001 vs rs25487
rs11615 vs rs238406
rs11615 vs rs25487
rs1051740 vs rs238406
rs1051740 vs rs25487
rs1051740 vs rs25489
rs2228001 vs rs25489
rs238406 vs rs25489
rs25489 vs rs3212950
rs25489 vs rs3212948
rs25487 vs rs3212950
rs25487 vs rs3212948
rs238406 vs rs3212950
rs238406 vs rs3212948
rs2228001 vs rs3212950
rs2228001 vs rs3212948
rs2228001 vs rs238406
rs11615 vs rs25489
rs11615 vs rs2228001
rs1051740 vs rs3212950
rs1051740 vs rs3212948
rs1051740 vs rs2228001
rs1051740 vs rs11615
0%
25%
50%
75%
100%
Percentage
1
1
1
1.4
1.1
1
1
1
1
1
1
1
1.2
1
1
1
1
1
1
1
1
1
1
1
1
1
1
1
0.0
0.5
1.0
1.5
Minimal Parallelism
1
1
1
1
1
1
1.1
1.1
1.4
1.6
2.4
1.8
1.9
3
3
2.1
2.1
2.1
2.1
3.1
3.1
2
2
2.1
3
3
2.6
2
0
1
2
3
Mean Path Length
