# Supplementary material for: The relationship between single nucleotide polymorphisms and skin cancer susceptibility: A systematic review and network meta-analysis
Source: Front Oncol. 2023 Feb 15;13:1094309. doi: 10.3389/fonc.2023.1094309 (PMC9975575; doi:10.3389/fonc.2023.1094309)
Supplement: Supplementary file 11 [file Presentation_1.pdf]

Search strategy

1. Pubmed (Results: 2315 articles)

((case) OR (control)) OR (case-control) AND ((((((((((Nucleotide Polymorphism[Title/Abstract]) OR Nucleotide Polymorphisms[Title/Abstract]) OR Polymorphisms[Title/Abstract]) OR Single Nucleotide Polymorphisms[Title/Abstract]) OR SNP[Title/Abstract]) OR Single Nucleotide Polymorphism[Title/Abstract])) OR (Single Nucleotide[Title/Abstract])) OR (Polymorphism[Title/Abstract])) OR (SNPs[Title/Abstract])) AND (((((((((((((((((('Skin Cancer'[Title/Abstract]) OR (melanoma[Title/Abstract])) OR (Non-Melanoma[Title/Abstract])) OR (Nonmelanoma[Title/Abstract])) OR ('basal cell carcinoma'[Title/Abstract])) OR ('basal-cell carcinoma'[Title/Abstract])) OR ('basal-cell cancer'[Title/Abstract])) OR ('squamous-cell carcinoma'[Title/Abstract])) OR ('squamous cell carcinoma'[Title/Abstract])) OR ('squamous-cell skin cancer'[Title/Abstract])) OR ('basal-cell skin cancer'[Title/Abstract])) OR ('squamous-cell cancer'[Title/Abstract])) OR ('basal cell cancer'[Title/Abstract])) OR ('basal cell skin cancer'[Title/Abstract])) OR ('squamous cell cancer'[Title/Abstract])) OR ('basal cell skin cancer'[Title/Abstract])) OR ('Cutaneous Melanoma'[Title/Abstract])) OR ('Malignant Melanoma'[Title/Abstract])))

2. Web of Science (Results: 776 articles)

"((((TS=(Nucleotide Polymorphism*)) OR TS=(Single Nucleotide Polymorphism*)) OR TS=(SNP*)) OR TS=(Single Nucleotide)) AND (((((((TS=('Skin Cancer')) OR TS=(*melanoma)) OR TS=(Nonmelanoma)) OR TS=('basal*cell carcinoma')) OR TS=('basal*cell cancer')) OR TS=('squamous*cell carcinoma')) OR TS=('squamous*cell skin cancer')) AND (((ALL=(case)) OR ALL=(control)) OR ALL=(case-control))"

3. Embase (Results: 484 articles)

#1. 'skin cancer':ti,ab,kw OR melanoma:ti,ab,kw OR 'non melanoma skin cancer':ti,ab,kw OR 'skin carcinoma':ti,ab,kw OR 'basal cell carcinoma':ti,ab,kw OR 'squamous cell carcinoma':ti,ab,kw

#2. 'nucleotide polymorphism*':ti,ab,kw OR 'single nucleotide polymorphism':ti,ab,kw OR 'single nucleotide':ti,ab,kw OR snp:ti,ab,kw

#3. 'case control study'

#1 AND #2 AND #3
