# Supplementary material for: The relationship between single nucleotide polymorphisms and skin cancer susceptibility: A systematic review and network meta-analysis
Source: Front Oncol. 2023 Feb 15;13:1094309. doi: 10.3389/fonc.2023.1094309 (PMC9975575; doi:10.3389/fonc.2023.1094309)
Supplement: Supplementary file 12 [file Presentation_2.pdf]

1. Inclusion and Exclusion Criteria

Inclusion criteria were as follows: (1) study type is case-control trial (including Nested case-control study); (2) case group is the patients diagnosed with SC, CM, NM, SCC, and/or BCC; (3) control group is the non-such cancer healthy population; (4) genotypic detection method is PCR or DNA sequencing, such as PCR-based restriction fragment length polymorphism (PCR-RFLP), TaqMan PCR, Real-Time PCR (RT-PCR), Kompetitive allele specific PCR (KASP PCR), PCR with sequence-specific primers (SSP-PCR), Taqman, Cycle Sequencing Kit, and SNaPshot Sequencing Kit.

Articles were excluded based on the criteria: (1) cohort study, case reports, reviews, meeting abstracts, or comments; (2) uveal melanomas, non-skin squamous cell carcinoma (such as esophageal, neck, oral squamous cell carcinoma); (3) irrelevant or family cancer case group or unhealthy population control group; (4) genotypic detection method is not PCR or DNA sequencing (in particular, using microarray detection Genome-Wide Association Studies (GWAS); (5) lack of available genotype frequency; (6) duplicated articles or data.

It is important to point out that, for the fifth exclusion criterion, due to the same population (cases were melanoma patients at the Melanoma and

Sarcoma Surgery Unit of the Istituto Nazionale Tumori, Milan, from May 2006 to June 2007. And controls were healthy donors from the Immunohematology and Transfusion Medicine Department, Fondazione IRCCS Istituto Nazionale Tumori), the same SNP (rs2910164), and the same conclusion (rs2910164 is a risk factor for melanoma), but the case/control number of study of Sangalli A[1] (304/314) is larger than that of Gomez-Lira M[2] (224/264). So we excluded Gomez-Lira M 's study, named ‘Association of microRNA 146a polymorphism rs2910164 and the risk of melanoma in an Italian population’. In the same manner, we excluded the 'Genetic Variants of the Vitamin D Receptor Gene Alter Risk of Cutaneous Melanoma' article[3] and only kept 'Haplotype and genotypes of the VDR gene and cutaneous melanoma risk in non-Hispanic whites in Texas: A case-control study' article[4].

Reference

1. Sangalli, A. *et al.* Sex-specific effect of RNASEL rs486907 and miR-146a rs2910164 polymorphisms’ interaction as a susceptibility factor for melanoma skin cancer. *Melanoma Res* **27**, 309–314 (2017).
2. Gomez-Lira, M. *et al.* Association of microRNA 146a polymorphism rs2910164 and the risk of melanoma in an Italian population. *Experimental Dermatology* **24**, 794–795 (2015).
3. Li, C. *et al.* Genetic Variants of the Vitamin D Receptor Gene Alter Risk

of Cutaneous Melanoma. *J Invest Dermatol* **127**, 276–280 (2007).

4. Li, C. *et al.* Haplotype and genotypes of the VDR gene and cutaneous melanoma risk in non-Hispanic whites in Texas: A case–control study. *International Journal of Cancer* **122**, 2077–2084 (2008).

2. Involved packages

1) packages employed in the R studio

```
install.packages("meta")
install.packages("netmeta")
```

2) packages used in the StataSE

```
net install midas.pkg, from(http://fmwww.bc.edu/RePEc/bocode/m/)
net install st0411.pkg, from(http://www.stata-journal.com/software/sj15-4/)
net install st0410.pkg, from(http://www.stata-journal.com/software/sj15-4/)
```
